# Targeted metabolomics of postmortem human cardiac tissue using the Biocrates MxP Quant 500 kit

**DOI:** 10.1371/journal.pone.0356269

**Published:** 2026-08-19

**Authors:** Oscar Emil Puntervold, Pia Johansson Heinsvig, Mads Gustav Skytte, Kristine Boisen Olsen, Jytte Banner, Jacob Tfelt-Hansen, Kristian Linnet, Marie Mardal

**Affiliations:** 1 Department of Forensic Medicine, University of Copenhagen, Copenhagen, Denmark; 2 Department of Cardiology, Rigshospitalet, Copenhagen University Hospital, Copenhagen, Denmark; 3 Department of Pharmacy, UiT, The Arctic University of Norway, Tromsø, Norway; Chang Gung University, TAIWAN

## Abstract

**Aim:**

This proof-of-concept study aimed to evaluate the feasibility and analytical performance of the Biocrates MxP® Quant 500 kit, originally developed for biofluids, to postmortem human cardiac tissue obtained from forensic autopsies, evaluating its potential as a standardized, cost-effective alternative to complex, resource-intensive metabolomics workflows.

**Methods:**

Left ventricular tissue samples were collected from 40 forensic autopsy cases, comprising 10 decedents with type 2 diabetes, 20 decedents with ischemic heart disease without type 2 diabetes, and 10 control cases without cardiac pathology. Cases were selected to represent the range of myocardial conditions commonly encountered in forensic practice, enabling assessment of analytical feasibility across heterogeneous postmortem cardiac tissue. Samples were analyzed using the MxP® Quant 500 kit following the standard protocol and using liquid chromatography–tandem mass spectrometry and flow injection analysis methods, measuring and quantifying a total of 630 endogenous metabolites across diverse classes.

**Results:**

Out of the 630 metabolites, 463 (74%) were within the quantifiable range. Lipid-related metabolites were notably well represented, with sphingomyelins (100% retained), phosphatidylcholines (93% retained), triacylglycerols (82% retained), and fatty acids (83% retained) showing the highest retention. Other metabolite classes such as acylcarnitines (45% retained) demonstrated greater variability, with some measurements falling below the limit of detection (e.g., 47% of acylcarnitines below this limit) or exceeding the upper limit of quantification (e.g., 35% of amino acids above this limit). Univariate analyses showed nominal group differences among specific metabolite subclasses (unadjusted p < 0.05). However, no metabolites remained statistically significant after correcting for false discovery rate. Multivariate analysis using PERMANOVA or PCA showed no strong global separation.

**Conclusion:**

The Biocrates MxP® Quant 500 kit demonstrated technical feasibility for postmortem cardiac tissue analysis, enabling quantification of a broad range of metabolites, particularly lipids. While variability was observed across certain metabolite classes, the approach provides a promising basis for standardized metabolomic investigations in forensic and cardiovascular research.

## Introduction

Postmortem cardiac tissue represents a valuable but analytically challenging matrix for metabolomics research. Autopsy samples may provide a unique snapshot of metabolic alterations prior to and at the time of death, but their analysis and interpretation are complicated by the postmortem degradation, in combination with incomplete information regarding the circumstances and timing of death. Although postmortem interval (PMI) is frequently used as a temporal marker, it is only a crude proxy for degradation, as factors such as temperature, humidity and environmental exposure may substantially affect tissue degradation [1,2]. Despite these challenges, postmortem metabolomic profiling is increasingly utilized in forensic and biomedical research, particularly for identifying disease-related biochemical alterations and biomarkers [3–5]. Given the potential value of postmortem cardiac tissue, standardized targeted metabolomics platforms should be evaluated in this matrix to determine whether they can generate quantifiable and biologically interpretable data under forensic autopsy conditions.

Metabolomics, the holistic analysis of small molecules involved in cellular metabolism, has emerged as a promising approach for studying biochemical mechanisms in postmortem material [3,5–7]. Metabolomics approaches are divided into targeted and untargeted strategies. Untargeted metabolomics aims to broadly profile metabolites without prior selection, enabling the discovery of novel biomarkers and unexpected metabolic alterations [7]. However, these workflows are technically demanding, produce highly complex datasets with many unidentified features, and are particularly challenging to standardize due to variability in sample preparation, analytical platforms, and data processing pipelines [8]. Targeted metabolomics, in contrast, quantifies a predefined set of metabolites with high reproducibility, allowing the validation of known biomarkers [7,9]. Nevertheless, like untargeted approaches, targeted methods also require substantial setup in the laboratory. Developing a robust targeted assay involves time-consuming optimization and access to authentic standards to ensure accurate metabolite identification and quantification [9]. This need for specialized resources can limit accessibility and standardization across laboratories.

Commercial kits provide a practical solution to streamline and standardize targeted metabolomics workflows. The Biocrates MxP® Quant 500 kit (Biocrates Life Sciences, Innsbruck, Austria) is widely used to quantify metabolites and lipids across key metabolic pathways using liquid chromatography–tandem mass spectrometry (LC-MS/MS) and flow injection analysis–tandem mass spectrometry (FIA-MS/MS). Although originally validated for human plasma, serum, and urine, this kit has also been applied to matrices such as feces, breast milk, liver, bone marrow, and postmortem brain tissue [10–13]. Its standardized protocol facilitates implementation across laboratories and improves comparability of results, but whether it is applicable to postmortem human cardiac tissue has not been established.

A biologically relevant setting in which to explore this feasibility is sudden cardiac death (SCD), for which autopsy in a hospital or forensic setting is strongly recommended [14–18]. Ischemic heart disease (IHD) is the leading cause of SCD, which typically occurs as a consequence of severe atherosclerotic stenosis or acute coronary occlusion with thrombosis [15,19,20]. Determining the cause of death in SCD remains challenging, particularly when macroscopic and microscopic findings are inconclusive or absent. In cases lacking an occlusive thrombus or significant coronary stenosis, establishing a diagnosis of ischemia-induced arrhythmia is often impossible. In this context, metabolomic profiling of postmortem cardiac tissue may provide additional biochemical insights into the myocardial metabolism in fatal cardiac conditions.

Type 2 diabetes (T2D) is a well-known risk factor for developing cardiovascular disease, especially IHD. Although less well characterized, T2D may also lead to diabetic cardiomyopathy, which can in turn lead to fatal cardiac dysfunction even in the absence of significant coronary artery disease [21–26]. As the global prevalence of T2D continues to rise, its contribution to cardiac deaths is becoming increasingly important in forensic investigations [27,28].

Both IHD and T2D are known to induce significant metabolic alterations in the myocardium. IHD is primarily characterized by ischemia-induced shifts in energy metabolism, including impaired oxidative phosphorylation and increased reliance on glycolysis [7,29]. In contrast, T2D is typically characterized by chronic metabolic remodeling, impaired glucose uptake, and increased fatty acid oxidation [22,30,31]. These alterations ultimately contribute to mitochondrial dysfunction, oxidative stress, and energy inflexibility, which can be detected through metabolomic profiling [4].

In this proof-of-concept study, we evaluated the feasibility of using the Biocrates MxP® Quant 500 kit to analyze human cardiac tissue obtained during routine forensic autopsies. To assess whether biologically relevant variation could be captured within the kit’s quantifiable range under realistic forensic conditions, we included cases of IHD, T2D, and controls without cardiac pathology, representing a spectrum of myocardial conditions commonly encountered in forensic practice. Our primary objective was to determine whether this targeted metabolomic approach can generate quantifiable metabolomic data from postmortem cardiac tissue under common forensic conditions, while group-based biological interpretations were considered exploratory.

## Materials and methods

### Study population

Forensic autopsies were performed in accordance with Danish legislation and followed international guidelines [15]. During autopsy, biopsies are routinely collected from most organs, which can later be used for ancillary purposes, such as toxicological, microscopic, or genetic analyses.

We included 40 male decedents aged 40–50 years, who underwent forensic autopsy at the Department of Forensic Medicine, University of Copenhagen, between 2018 and 2023. This age interval was chosen as, in Denmark, cardiac tissue is not routinely collected for storage at −70°C for individuals aged > 50 years, but we still wanted to include individuals with overt cardiovascular disease. To minimize sex-related metabolic variability and ensure a more homogeneous sample set, only male cases were included.

All forensic autopsies within the target age range were screened. Data from police reports, autopsy records, histological examinations, toxicological screenings (including postmortem HbA1c analysis), and general practitioner records were reviewed to determine case eligibility. Diagnoses of T2D were confirmed based on at least one of the following criteria: HbA1c > 48 mmol/mol [32], a history of being prescribed glucose-lowering agents (ATC-A10), or a medical history of T2D based on information from the decedent’s general practitioner. IHD was defined by the presence of ≥75% coronary artery stenosis observed macroscopically during the autopsy or histopathological findings consistent with chronic or acute myocardial ischemia [33]. Control individuals were selected from cases of sudden death due to accidental trauma, including traffic accidents (involving pedestrians, cyclists, motorcyclists, or car drivers) or falls from height, where death was considered instant and not disease-related.

Exclusion criteria were evidence of putrefaction (e.g., greenish discoloration of the abdominal skin), cardiac trauma, a history of substance abuse, and body mass index (BMI) > 35 or < 20. To minimize the potential confounding effects of a prolonged PMI, cases with the shortest PMI (ranging from 3 to 9 days) were selected and categorized into three groups: decedents with confirmed T2D whose causes of death included both IHD and accidental trauma (n = 10); decedents with confirmed acute or chronic IHD without evidence of T2D (n = 20); and a control group consisting of decedents without T2D or IHD who died from accidental trauma (n = 10). It should be noted that the recorded PMI is subject to uncertainty depending on the circumstances surrounding the death, and for this reason cases with visible signs of putrefaction were excluded as a pragmatic measure to limit bias from advanced postmortem degradation.

Cases were identified and data were accessed for research purposes in June–July 2024. During case identification, one author had access to identifiable information within a secure forensic database solely to determine eligibility. Following case selection, tissue samples and associated data were anonymized by authorized personnel at the Department of Forensic Medicine prior to release for analysis. The identification key was deleted before data analysis. The authors did not have access to directly identifiable information during or after data analysis. The baseline characteristics available for analysis did not permit identification of individual decedents. This included age, PMI, BMI, heart weight, left ventricular wall thickness, and coronary artery stenosis measured at autopsy. The stenosis of the arteries (left anterior descending artery, LAD, right coronary artery, RCA, and left circumflex artery, Cx) were categorized into mild (0–25%), moderate (26–50%), moderately severe (51–75%) or severe (>75%).

### Tissue sampling

Cardiac tissue samples were collected during routine forensic autopsy from the anterior wall of the left ventricle at the mid-ventricular level (2×1 cm transverse sample). The samples were placed in cryotubes and stored at −70°C immediately after the autopsy and later thawed and homogenized at a 1:3 ratio with isopropanol. This single extraction with 100% isopropanol was selected based on the procedure reported by Andresen et al. [10] who evaluated different extraction media and tissue-to-solvent ratios for human tissue analyzed with the same kit. The tissue was homogenized using a gentleMACS Octo Dissociator (Miltenyi Biotec, Bergisch Gladbach, Germany) with a 50–second program applying up to 4000 rpm. The instrumentation is routinely used for postmortem muscle and brain tissue homogenization prior to quantitative analysis in forensic case work with the same tissue-to-solvent ratio, although different extraction media optimized for a different group of analytes [10,34,35]. Approximately 500 mg of tissue was processed per case, and homogenate supernatants were stored at −70°C until further analysis.

### Chemicals and reagents

All reagents and solvents were of LC-MS grade unless otherwise specified. MilliQ® water was obtained from a local MilliQ water system. Ethanol, methanol, acetonitrile, isopropanol, and ammonium acetate were purchased from Merck Life Science (Darmstadt, Germany). Formic acid was sourced from Honeywell Fluka™ (Charlotte, NC, USA), phenyl isothiocyanate (PITC) from Sigma-Aldrich (St. Louis, MO, USA), and pyridine (purity > 99%) from Thermo Scientific (Waltham, MA, USA).

### Targeted metabolomics

For targeted metabolomics with the Biocrates MxP® Quant 500 kit, samples were prepared and analyzed in accordance with the manufacturer’s instructions. Ten microliters of the tissue extract was used for the sample preparation. The kits were analyzed on a Waters TQ-XS coupled with a Waters I-class UPLC (Waters Corp., Milford, MA, USA), with two liquid chromatography–tandem mass spectrometry (LC-MS/MS) and two flow-injection analysis (FIA)-MS/MS methods per sample [36]. Further information on sample preparation and instrumentation is available in the S1 File, Materials and methods, Supplementary Information.

### Data acquisition and processing

Data acquisition was performed using MassLynx version 4.2 (Waters Corporation, Massachusetts, USA). Raw data were then processed with Biocrates’ cloud-based WebIDQ software for quantification, quality control assessment, and batch normalization in accordance with the kit specifications. All values were normalized to the median value of a quality control sample analyzed in four replicates throughout the run. For downstream analysis, missing values were imputed based on the limit of detection (LOD). Specifically, concentrations reported as below the LOD were imputed as LOD/2. When no valid LOD value was available for a given metabolite, the value was imputed as half of the lowest observed concentration for that metabolite across all samples. After preprocessing, the data were log2-transformed and exported as an  .xlsx file. The dataset was extracted with and without a filter for metabolites quantified in at least 75% of samples in at least one study group to evaluate which metabolites were retained. The results presented are thus based on metabolites that were measured in at least 75% of samples in one study group. Metabolite groups were adapted from Biocrates.

### Statistical analysis

As only 54% of metabolites met the assumption of normality, as assessed by the Shapiro–Wilk test, group comparisons of metabolites and continuous baseline variables were conducted using the non-parametric Kruskal–Wallis test. Categorical baseline variables were compared using Fisher’s exact or chi-square tests, as appropriate. A p-value < 0.05 was considered statistically significant. To correct for multiple testing, p-values were adjusted using the Benjamini–Hochberg procedure to control the false discovery rate (FDR), applied across all retained metabolites. Effect sizes were estimated using epsilon squared (ε²), with ε² values > 0.14 considered as large effect sizes. For metabolites showing significant differences using the Kruskal–Wallis test, post hoc pairwise comparisons were performed using Dunn’s test to identify which specific groups differed. To assess differences in overall metabolite profiles between groups, a permutational multivariate analysis of variance (PERMANOVA) was performed using Bray–Curtis dissimilarities. The test was conducted using 999 permutations. Homogeneity of multivariate dispersion was assessed using permutation tests (PERMDISP), as differences in dispersion may influence PERMANOVA results.

To investigate the influence of PMI on metabolite profiles, metabolite concentrations were standardized as z-scores across samples to account for differences in concentration scales. Z-scores were then averaged within metabolite classes to generate class-level scores for each sample. Associations between metabolite class scores and PMI were assessed using Spearman’s rank correlation, with FDR adjusted p-values as described above.

Data visualization included boxplots to illustrate group-wise differences in metabolite concentrations, volcano plots to represent both statistical significance (–log_10_ p-value) and effect size (log_2_ fold change), and multivariate methods to explore global patterns in the data. Principal component analysis (PCA) was used as an unsupervised method for assessing natural variance and clustering across groups using autoscaled metabolite concentrations.

All statistical analyses were performed in R (version 4.3.1; R Foundation for Statistical Computing, Vienna, Austria). The full R script and anonymized metabolomics datasets used for the analysis are available on GitHub (https://github.com/opdjmw/MxP-Quant-500-postmortem-analysis).

### Ethics statement

This study was a proof-of-concept and methodology study using postmortem cardiac tissue collected during routine forensic autopsies. The study was reviewed and approved by the Danish Research Ethics Committee (De Videnskabsetiske Komitéer, Region Hovedstaden; journal no. H-23019754). All tissue samples and associated data were fully anonymized prior to analysis.

## Results

### Study population

Five characteristics of the study population were reported: age, body mass index (BMI), PMI, heart weight, and coronary artery stenosis, as shown in Table 1. The mean age was comparable across groups (IHD 45 years, controls 45.2 years, and T2D 47 years, p = 0.130). PMI was slightly longer in controls (mean 5.8 days) than in the disease groups (IHD 4.2 days and T2D 4.4 days), however overall similar between the groups (p = 0.109). BMI was similar across groups, ranging from 29.6 to 30.7 kg/m² (p = 0.863). Although not significant, heart weight tended to be highest in the T2D group (506 g), followed by the IHD group (464 g) and the controls (424 g) (p = 0.075). Left ventricular wall thickness did not differ substantially among the groups (IHD 12.5 mm, T2D 12.1 mm, and controls 12.1 mm, p = 0.661).

**Table 1:** Baseline characteristics of the study population.

Variable	Controls (n = 10)	T2D (n = 10)	IHD (n = 20)	p-value
Age	45.2 ± 3	47 ± 2	45 ± 2.5	0.130
PMI	5.8 ± 2.2	4.4 ± 1.3	4.2 ± 1.4	0.109
BMI	29.7 ± 3.8	30.7 ± 4.2	29.6 ± 4.8	0.863
Heart weight [g]	423.7 ± 62.6	506.3 ± 101	464.2 ± 63.4	0.075
LV wall thickness [mm]	12.1 ± 1.4	12.1 ± 3.6	12.5 ± 2.6	0.661
LAD stenosis category, n	Mild: 9; Moderate: 1	Mild: 3; Moderate: 2; Moderately severe: 4; Severe: 1	Mild: 2; Moderate: 1; Moderately severe: 10; Severe: 7	<0.001
RCA stenosis category	Mild: 10	Mild: 5;Moderate: 2; Moderately severe: 1; Severe: 2	Mild: 8;Moderate: 4; Moderately severe: 5;Severe: 3	0.081
Cx stenosis category	Mild: 10	Mild: 6;Moderate: 1; Moderately severe: 2; Severe: 1	Mild: 12;Moderate: 3; Moderately severe: 5	0.196

Variables are given as mean ± SD*.* PMI: postmortem interval, BMI: body mass index, LV, left ventricular, LAD, left anterior descending artery, RCA, right coronary artery, Cx, left circumflex artery

Most IHD cases displayed at least moderate stenosis of the LAD, the majority of controls had mild stenosis, while T2D cases showed an intermediate pattern, with a distribution spanning mild to severe stenosis (p < 0.001). For RCA and Cx, all controls had mild stenosis, while IHD and T2D groups showed broader distributions, although these differences were less pronounced and not statistically significant (RCA p = 0.081; Cx p = 0.196).

### Evaluation of metabolite detection in postmortem cardiac tissue

**Fig 1:**
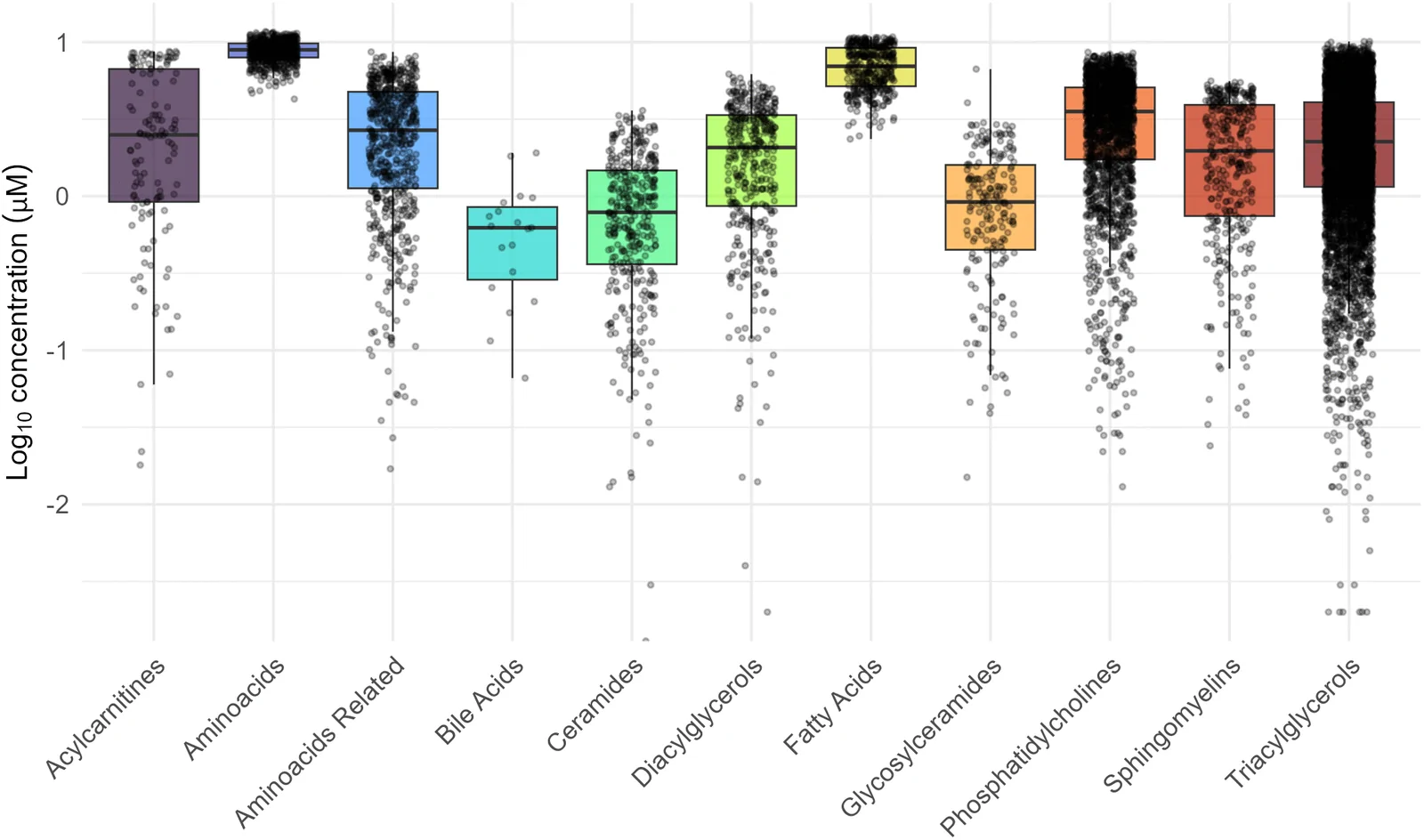
Distribution of log10 concentration (µM) by metabolite class for classes with eight or more metabolites. Each box represents the interquartile range (IQR), with the median as a horizontal line; whiskers indicate 1.5 × IQR. Individual data points are overlaid.

For downstream analyses, we retained only those metabolites that were detected in at least 75% of the samples within at least one study group. Out of the 630 metabolites measured, 463 (74%) met this criterion, as detailed in Table 2 and a more detailed analytical summary of all 630 metabolites is presented in S1 Table. The results show that metabolite retention varies across metabolite classes. For example, alkaloids, cresols, sphingomyelins, vitamins and cofactors, and nucleobases-related showed complete retention, while dihydroceramides (12.5%) and cholesterol esters (13.6%) had the lowest detection rates. Fig 1 illustrates the concentration ranges by metabolite class. Some metabolites showed large variations. For example, methionine sulfoxide ranged from <LOD to> ULOQ, taurine measurements were > ULOQ in all samples, and CE 17:0 was < LOD in all samples. Acylcarnitines, dihydroceramides, and hormones generally displayed low concentrations, with a high proportion of values below the LOD. In contrast, amino acids and fatty acids showed signal saturation, with > 30% of values above the ULOQ. Despite these extremes, key metabolites were retained in classes such as amino acids (80%), amino acid-related (83.3%), and fatty acids (83.3%). S2 Table presents the concentration ranges of the retained metabolites as a reference.

**Table 2:** Detected metabolite classes.

Class	<LOD, %	>ULOQ, %	Retained, %
Acylcarnitines (n = 40)	46.9	2.6	45.0
Amino acids (n = 20)	0.0	34.5	80.0
Amino acid-related (n = 30)	13.4	0.2	83.3
Bile acids (n = 14)	14.1	0.0	85.7
Biogenic amines (n = 9)	36.7	0.0	66.7
Ceramides (n = 28)	84.7	0.2	96.4
Cholesterol esters (n = 22)	59.5	0.1	13.6
Diacylglycerols (n = 44)	72.5	0.0	40.9
Dihydroceramides (n = 8)	9.8	37.5	12.5
Fatty acids (n = 12)	45.3	1.9	83.3
Glycosylceramides (n = 35)	6.3	1.7	51.4
Phosphatidylcholines (n = 88)	0.0	0.0	93.2
Sphingomyelins (n = 14)	21.0	0.1	100
Triacylglycerols (n = 242)	46.9	2.6	82.2

Alkaloids (n = 1), biogenic amines (n = 6), carboxylic acids (n = 4), cholesterol esters (n = 3), dihydroceramides (n = 1), hormones (n = 2), indoles and derivatives (n = 3), nucleobases-related (n = 1), and vitamins and cofactors (n = 1) were not included due to having fewer than eight detected metabolites per class and can be found in S2 Table. LOD: limit of detection, ULOQ: upper limit of quantification

### Postmortem interval

Spearman correlation analysis of metabolite class scores and PMI showed moderate positive correlations for several classes, including biogenic amines (ρ = 0.43, p = 0.006), fatty acids (ρ = 0.43, p = 0.006), glycosylceramides (ρ = 0.42, p = 0.008), and amino acid-related metabolites (ρ = 0.39, p = 0.013). However, none of these associations remained statistically significant after FDR correction. The remaining metabolite classes showed weak or no correlations with PMI (S3 Table).

### Unsupervised analysis

**Fig 2:**
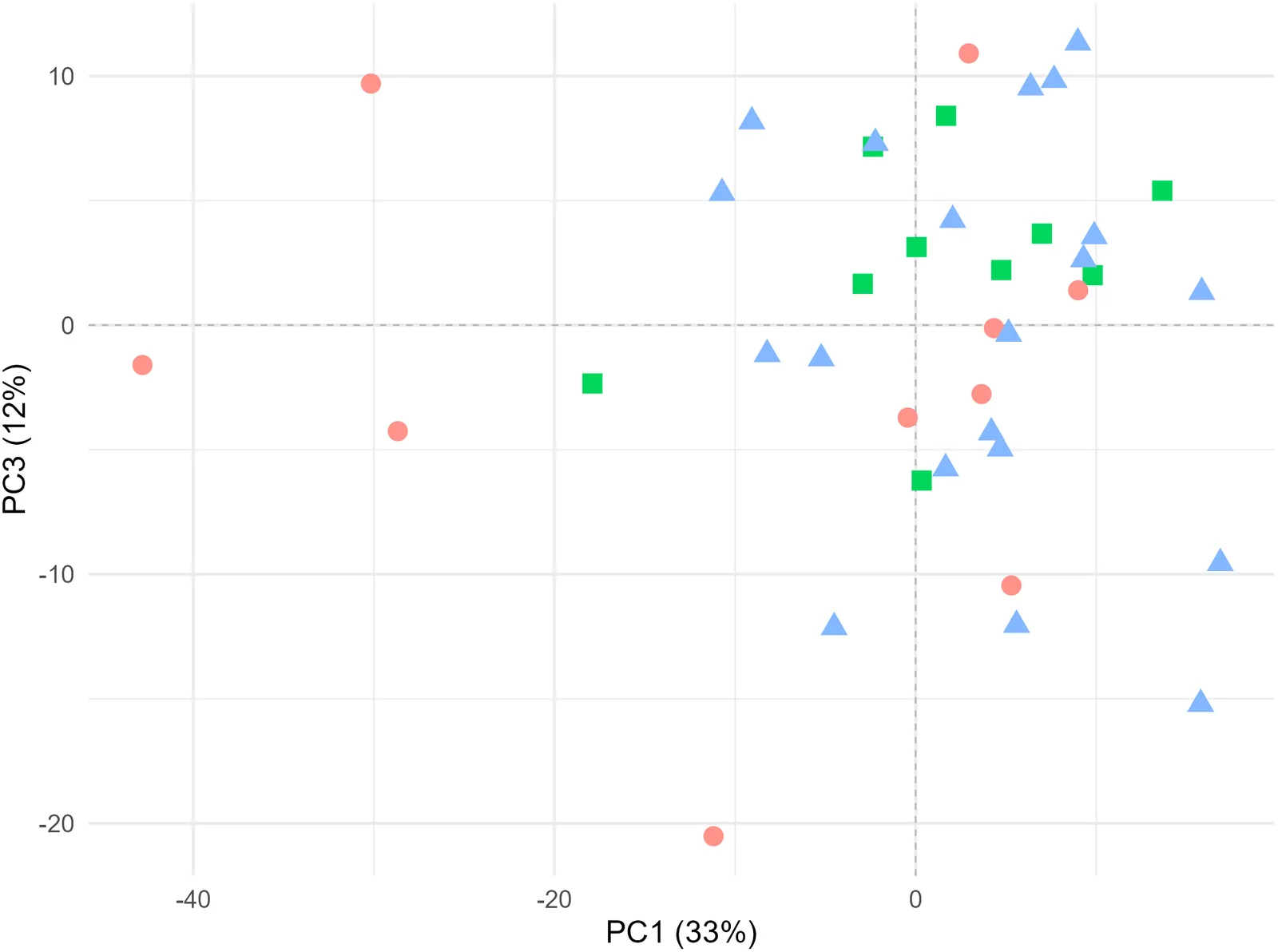
Principal component analysis (PCA) showing unsupervised clustering of individuals based on metabolite profiles. Each point represents one individual, color-coded by group: T2D (○, red), IHD (Δ, blue), and Control (□, green). PC1 and PC3 are visualized, revealing partial overlaps and marginal clustering between groups.

PCA was performed to explore the overall variance in metabolite profiles. PC1 accounted for 33% of the variance, while PC2 and PC3 each accounted for 12%. Eigenvalues and variance for PC1–10 are listed in S4 Table. In the score plot (Fig 2), partial separation of the groups was observed, particularly along PC1, although substantial overlap remained across all groups. The T2D group displayed a greater variance than the IHD and control groups, suggesting higher within-group heterogeneity. When samples were annotated according to PMI ranges of <4 days, 4–7 days, and ≥7 days, no distinct clustering was observed (S1 Fig). Overall, PCA did not provide clear separation by either groups or PMI, suggesting that the overall metabolic variation is not strongly driven by these factors. The metabolites contributing most strongly to PC1–3 included triacylglycerols (TG 18:1_34:2 on PC1), fatty acids (FA 20:3 on PC2), and phosphatidylcholines (PC 40:6 on PC3).

### Inter-class variance in metabolite profiles

After performing the Kruskal–Wallis test on the 463 retained metabolites, 19 metabolites showed nominally significant differences (unadjusted p < 0.05) between the groups, as seen in Table 3. However, no metabolites remained statistically significant after FDR correction. The adjusted p-values were high (above 1, reported as p = 1.00), reflecting the distribution of p-values across all tested metabolites and the large number of comparisons. Despite the lack of statistical significance, several metabolites showed a relatively large effect sizes, such as acylcarnitines C3 (ε² = 0.24), C2 (ε² = 0.17), and phosphatidylcholine PC O-34:3 (ε² = 0.21). If we look at the analytical performance of the metabolites with unadjusted p < 0.05 in the Kruskal-Wallis test (Table 3), the triglycerides and PC O-30:1 had at least one failed QC (S1 Table), either for the coefficient of variation or accuracy, measured at three levels with at least three QCs at each level. At least one failed QC for one parameter is indicated with an asterisk in Table 3. The remaining ten metabolites passed all set parameters, with the medium-level QC coefficient of variation below 12% (N = 4).

**Table 3:** Kruskal–Wallis test results (for metabolites with p < 0.05).

Metabolite (abbreviation, metabolite class)	$\epsilon^{2}$	p-value	adjusted p-value
C3, Acylcarnitine	0.255	0.003	1.00
PC O-34:3, Phosphatidylcholine	0.213	0.007	1.00
C2, Acylcarnitine	0.172	0.015	1.00
GABA, Biogenic amine	0.161	0.019	1.00
TG 22:6_32:1, Triacylglycerol*	0.154	0.021	1.00
PC O-36:4, Phosphatidylcholine	0.152	0.022	1.00
TG 20:5_34:1, Triacylglycerol*	0.140	0.028	1.00
C0, Acylcarnitine	0.134	0.031	1.00
TG 17:2_36:2*,* Triacylglycerol*	0.129	0.034	1.00
TG 18:1_28:1, Triacylglycerol*	0.128	0.034	1.00
SM 34:2, Sphingomyelin	0.128	0.035	1.00
TG 18:1_30:2, Triacylglycerol*	0.125	0.036	1.00
Harg, Amino acid-related	0.124	0.037	1.00
TG 18:1_34:4, Triacylglycerol*	0.123	0.038	1.00
PC O-30:1, Phosphatidylcholine*	0.123	0.038	1.00
TG 18:2_30:1, Triacylglycerol*	0.117	0.043	1.00
C18:2, Acylcarnitine	0.114	0.044	1.00
TG 16:1_36:2, Triacylglycerol*	0.114	0.044	1.00
PC 32:3, Phosphatidylcholine	0.111	0.047	1.00
TG 16:0_34:4, Triacylglycerol*	0.108	0.050	1.00

*: At least one QC fail for either coefficient of variation or accuracy, measured at three levels with at least three QCs at each level (see S1 Table)

PERMANOVA showed no significant differences in overall metabolite composition between groups (R² = 0.051, F = 1.00, p = 0.429), suggesting that only a small proportion of the overall variance in the dataset was attributable to inter-group variation. However, testing for homogeneity of dispersion revealed significant differences in within-group variability (PERMDISP, p = 0.016), indicating that dispersion differed between groups. This suggests that the lack of group separation observed may reflect differences in within-group heterogeneity rather than distinct group-specific metabolic profiles.

**Fig 3:**
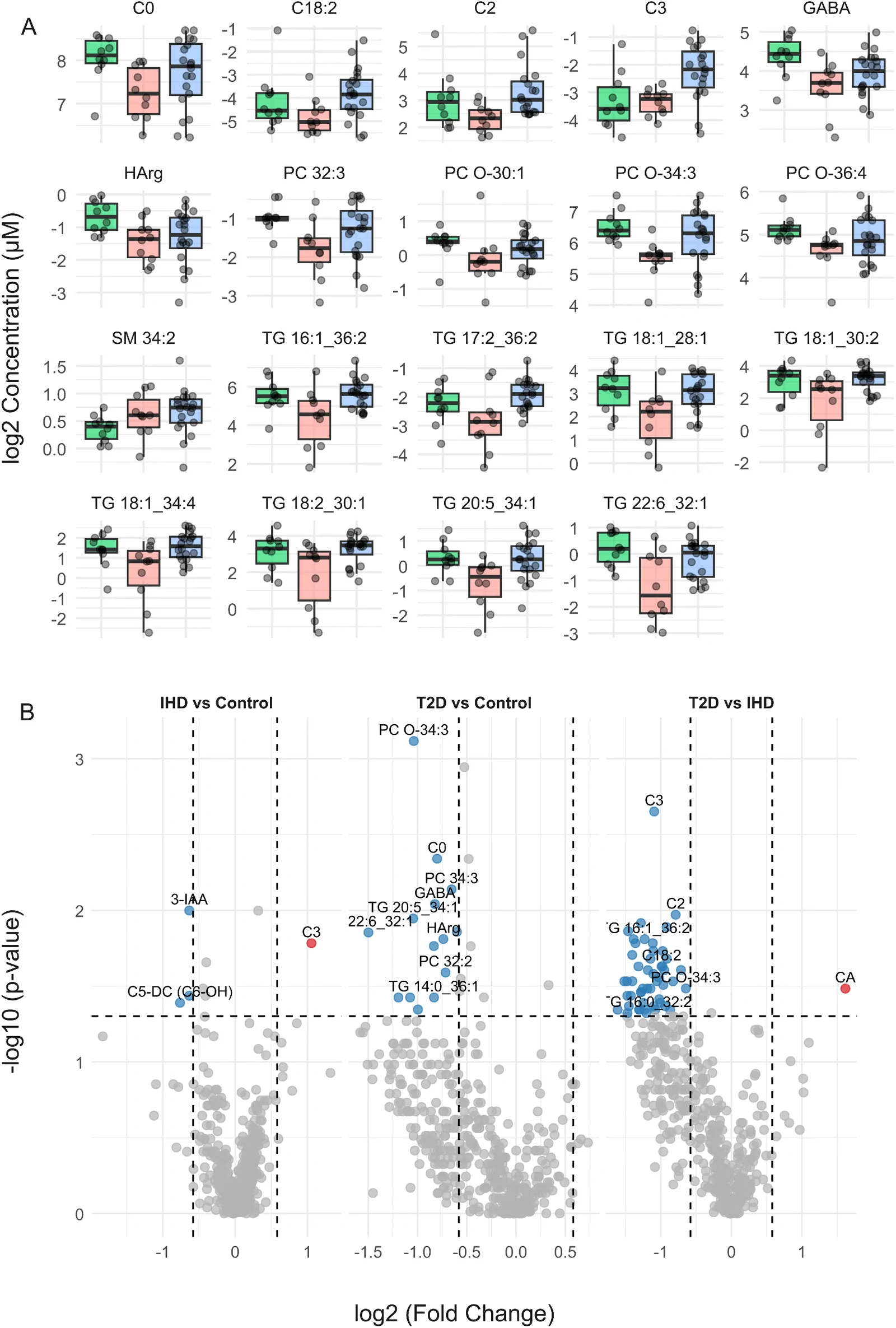
A) Boxplots of 19 metabolites with nominally significant differences between groups (Control, IHD, T2D), identified by Kruskal–Wallis test followed by post hoc Dunn’s test (FDR-corrected p < 0.05; see S5 Table for details). Metabolite concentrations are log2-transformed and shown in µM. Each box represents the interquartile range (IQR), with the median as a horizontal line; whiskers indicate 1.5 × IQR. Individual data points are overlaid. B) Volcano plots showing pairwise comparisons of retained metabolites in cardiac tissue: IHD vs. Control, T2D vs. Control, and T2D vs. IHD. The x-axis shows log_2_ fold change, and the y-axis shows –log₁₀ p-value. Metabolites surpassing significance thresholds (p < 0.05 and log2 fold change > 1) are highlighted in red (upregulated) or blue (downregulated).

Boxplots and volcano plots, shown in Fig 3, were used to visualize groupwise metabolite differences based on effect size (log_2_ fold change) and statistical significance (−log_10_ p-value). The the box plots (Fig 3A) show metabolites with nominally significant differences across groups in the Kruskal–Wallis test using unadjusted p-values (p < 0.05), while the volcano plots (Fig 3B) provide an overview of all retained metabolites. In the comparison between IHD cases and controls, acylcarnitine C3 was upregulated in the IHD cases, while 3-indoleacetic acid (3-IAA) and homoarginine (HArg) were downregulated. For the T2D vs. control comparison, multiple triacylglycerols (e.g., TG 22:6_32:1, TG 14:0_36:1) and phosphatidylcholines (e.g., PC 34:1, PC O-36:3) showed marked downregulation in T2D. The T2D vs. IHD comparison revealed a similar trend of downregulation in short-chain acylcarnitines (e.g., C2, C3, C4) and triacylglycerols in the T2D cases, while a single bile acid (CA) was upregulated compared with the level in IHD. The full results of the post hoc Dunn’s tests are provided in S5 Table, and corresponding fold changes and unadjusted p-values for all metabolites included in the volcano plots are detailed in S6 Table.

## Discussion

### Rationale for the study design

T2D and IHD are common underlying conditions in SCD [17,24,26,37,38]. These groups were chosen to represent pathophysiologically distinct yet clinically relevant forms of cardiac disease [4,22,29,39], making them suitable for assessing the feasibility of applying targeted metabolomics to postmortem cardiac tissue.

Cases were broadly matched in terms of age, sex, BMI, and PMI. Although the individuals included in the study were generally younger and less representative of the broader population, either healthy or affected by IHD and T2D, they reflect the SCD population typically encountered in a forensic setting in Denmark [17]. The forensic autopsy setting enabled access to well-characterized cases with confirmed cardiac pathology and phenotypic specificity based on both macroscopic and histopathological assessment. This provided a suitable setting for testing methodological feasibility in a context where traditional autopsy findings may be inconclusive. Moreover, the ability to analyze cardiac tissue, normally inaccessible in living patients, enhances the translational relevance of the study.

### Feasibility of the commercial targeted metabolomics kit in postmortem cardiac tissue

Using the selected and standardized analytical setup, 463 out of the 630 evaluated metabolites (74%) were retained for downstream analysis, supporting the technical feasibility of using the MxP® Quant 500 kit on autopsy-derived cardiac tissue. This is a notable finding given the known challenges associated with postmortem samples, including variable PMI and potential tissue degradation. Remarkably, the number of retained metabolites is similar to what has been reported for fresh surgical liver tissue originating from living donors and extracted with a similar protocol and kit [10]. This demonstrates the analytical feasibility of the method under postmortem conditions.

Using the vendor-recommended extraction and a fixed sample input volume of 10 μL of homogenized tissue extract (see S1 File, Materials and Methods), we observed that this input amount may have been sub-optimal for certain metabolite classes. Several classes, such as acylcarnitines and biogenic amines, showed a high proportion of values below the LOD, suggesting that these analytes may have required a greater sample volume to increase the analyte concentration. Conversely, amino acids and fatty acids displayed saturation effects, with > 30% of values exceeding the ULOQ in some cases, indicating that these abundant analytes may benefit from sample dilution. Most fatty acids in this kit are analyzed by FIA-MS/MS, a platform with different analytical characteristics than LC-MS/MS, including dynamic range. This is reflected in a higher number of high- and low-level QCs with CV error in this group (S1 Table); if this group of analytes is of particular interest, a lipidomics method or, alternatively, the MxP Quant 500 XL kit would be a better choice.

Values below LOD were imputed with LOD/2 during data preprocessing. However, only metabolites that were quantified in at least 75% of one group were retained, meaning that if a metabolite was consistently measured below LOD, it would be excluded for downstream analysis. The imputation enables inclusion of all samples in multivariate and univariate analyses, which generally require complete numerical datasets, and this imputation assigns uniformly low values where we know the true value is between 0 and LOD. However, as several metabolite classes contained a high proportion of values below LOD, LOD/2 imputation may reduce variance and influence downstream statistics, and therefore these classes should be interpreted cautiously.

In addition to demonstrating the technical feasibility of using the MxP® Quant 500 kit, it is important to assess whether the detected metabolites represent biologically meaningful targets in the context of cardiovascular research. We observed good coverage of several lipid classes, including fatty acids, sphingomyelins, phosphatidylcholines, and triacylglycerols, which are associated with T2D and cardiometabolic disease [39] and have previously been analyzed in human cardiac and liver tissue [10,40]. However, as the specific metabolite classes that can be analyzed with the kit are predefined, a notable limitation in this context is the missing information of lactate, a key intermediate in glycolysis and a marker of ischemia, for which measurements were above the ULOQ (S2 Table). A notable group of metabolites not covered by this kit is cardiolipins (important for cardiac mitochondrial function) whose altered metabolism has been associated with cardiac and metabolic disorders [41]. Overall, the retained metabolite coverage included a broad panel relevant to mitochondrial function, lipid metabolism, and membrane biology, supporting the potential utility of this approach for cardiovascular research.

Postmortem-specific alterations must also be considered, as cellular metabolism does not cease immediately at the time of death [8]. Some metabolites, particularly those involved in energy metabolism (e.g., acylcarnitines involved in mitochondrial function), can be depleted after death, while other classes, such as amino acids and phosphatidylcholines, could reflect cellular breakdown following death [7,8]. Ideally, tissue would be collected immediately after death and snap-frozen to halt ongoing cellular metabolism [42]. In the context of forensic autopsies, where the PMI is often both unknown and extended, it is challenging to completely avoid the metabolic alterations associated with delayed sampling [1]. Despite challenges such as autolysis, anoxia, and variations in PMI, many metabolites remained sufficiently stable to allow for biological interpretation. PMI showed weak to moderate correlations with some metabolite classes, however, these associations were not statistically significant after FDR correction. Similarly, PCA did not demonstrate clear clustering by PMI, suggesting that while PMI contributes to variability, it is not a dominant driver of the overall metabolic profile within the studied interval.

### Metabolic differences among T2D, IHD, and controls

Exploratory multivariate analysis using PCA revealed overlap in the overall variance in metabolic profiles among the IHD, T2D, and control groups, indicating heterogeneity within study groups. PERMANOVA showed no significant differences in the overall metabolite profiles between groups, while tests of homogeneity of dispersion indicated significant differences in within-group variability. Together, these findings suggest that the observed variation may reflect differences in heterogeneity rather than distinct group-specific metabolic profiles, consistent with the overlap observed in PCA.

Univariate analyses identified 19 metabolites with nominal differences between the study groups (unadjusted p < 0.05), as listed in Table 3, however none remained statistically significant after FDR correction. Several metabolites showed moderate-to-large effect sizes (ε² > 0.14), suggesting potential differences that warrant further investigation. For example, acylcarnitine C3 showed higher levels in IHD cases compared with controls and T2D cases. This is consistent with previous reports of elevated acylcarnitine levels in patients with chronic ischemic heart failure have [43]. More broadly, individual metabolites within lipid classes such as phosphatidylcholines, ceramides and triacylglycerols, showed differences across the study groups, however these findings should be interpreted with caution given the lack of statistical significance after FDR correction.

Generally, any suggested biological variance fell on correcting for FDR. Only limited pre-filtering (metabolites must be quantified in at least 75% in at least one study group) was applied before the data analysis. It could be justifiable to include additional filters to reduce number of retained metabolites, such as dispersion ratio filters (removing metabolites with low biological-to-technical variance), or requiring metabolites to be quantified in multiple samples in all study groups. Lowering the number of tested variables would reduce the impact of multiple testing correction. Since this is a proof-of-concept study, it was decided to retain many metabolites also those that might have pronounced intergroup variance in analytical performance.

### Limitations

The composition of our study groups reflects pragmatic compromises. For example, to ensure adequate sample sizes, the IHD group included both chronic and acute (thrombosis) ischemia, and the T2D group had a mixture of causes of death, namely, both IHD and accidental trauma (similar to the control group). More refined case definitions were constrained by the available sample material. We limited our cohort to male individuals aged 40–50 years. As a result, our cohort may not capture the full extent of advanced cardiac pathology, nor allow assessment of sex-specific metabolic differences. In future work, clearer patient stratification, such as separating acute versus chronic ischemia or including better-defined T2D cases, as well as the inclusion of female and older individuals, could enhance the biological insights obtained. Furthermore, comorbidities such as undiagnosed T2D cannot be completely ruled out, particularly in IHD cases where HbA1c is not routinely measured unless diabetes is suspected. Finally, although PMI was restricted to a relatively narrow range (3–9 days), metabolite-specific degradation cannot be ruled out and may likely contribute to the observed variability, although its overall impact on the metabolic patterns appeared limited within the studied interval.

## Conclusions

This proof-of-concept study demonstrates the feasibility of applying a standardized, targeted metabolomics platform to postmortem human cardiac tissue. Among the 630 evaluated metabolites, 463 met the threshold criteria, representing metabolites from all major classes, although with class-dependent analytical performance. However, the absence of clear group clustering and the presence of substantial within-group variability indicate that the current findings should be interpreted as demonstrating analytical feasibility rather than disease-specific metabolic signatures.

Despite the challenges inherent to sampling in a forensic setting, the approach preserved measurable variation, which provides a foundation for future studies to integrate larger cohorts with more refined phenotyping to investigate disease-related metabolic patterns in human cardiac tissue.

## Supporting information

S1 Fig Principal component analysis stratified by postmortem interval (PMI). Principal component analysis (PCA) showing unsupervised clustering of individuals based on metabolite profiles, visualized by PC1 and PC3. Each point represents one individual, with shape indicating diagnostic group: ○T2D, △IHD, and □Control. Colors indicate PMI category: short PMI (<4 days), medium PMI (4–7 days), and long PMI (≥7 days). The plot was used to evaluate whether overall metabolite variation was associated with PMI, with no clear clustering according to PMI category observed.
(TIF)

S1 Table Summary table of analytical parameters for all metabolites, in µM. Summary of analytical run parameters for all 630 metabolites, including LOD, LLOQ, ULOQ, and QC performance on precision and accuracy.
(DOCX)

S2 Table Summary table of retained metabolites measured in heart tissue. Summary of retained metabolites measured in heart tissue, including metabolite class, concentration range, median concentration, and proportions of values below LOD/LLOQ or above ULOQ.
(DOCX)

S3 Table Spearman correlation between PMI and metabolite class scores. Spearman correlations between postmortem interval (PMI) and metabolite class scores, including rho (ρ) values, unadjusted- and FDR-adjusted p-values.
(DOCX)

S4 Table PCA variance and metabolite contributions. Eigenvalues, explained and cumulative variance for PC1–10, with the top contributing metabolite for each principal component.
(DOCX)

S5 Table Dunn’s post hoc test results. Pairwise Dunn’s post hoc test results for metabolites with significant Kruskal–Wallis tests, including Z-values, unadjusted- and FDR-adjusted p-values.
(DOCX)

S6 Table Volcano plot metabolite statistics. Log2 fold changes, p-values, pairwise comparisons, and direction of regulation for metabolites presented in the volcano plots.
(DOCX)

S1 File Materials and methods, Supplementary Information. Detailed description of sample preparation, LC-MS/MS and FIA-MS/MS instrumental analysis, system suitability testing, WebIDQ processing, and data export procedures for the Biocrates MxP® Quant 500 kit.
(DOCX)
