# Supplementary material for: Targeted metabolomics of postmortem human cardiac tissue using the Biocrates MxP Quant 500 kit
Source: PLoS One. 2026 Aug 19;21(8):e0356269. doi: 10.1371/journal.pone.0356269 (PMC13489478; doi:10.1371/journal.pone.0356269)
Supplement: Supplementary file 1 [file pone.0356269.s002.docx]

**S1 Table. Summary Table of Analytical Parameters for all Metabolites, in µM**

| Analyte | Median concentration | Analyte classification | LOD | LLOQ | ULOQ | Coefficient of variation | | | Accuracy | | | Precision |
| --- | --- | --- | --- | --- | --- | --- | --- | --- | --- | --- | --- | --- |
|  |  |  |  |  |  | # QC1 fail | # QC2 fail | # QC3 fail | # QC1  fail | # QC2 fail | # QC3 fail | QC2 RSD% |
| C0 | 238.856 | Quantitative type 1 | 1 | 5 | 120 | 0/3 | 0/4 | 0/4 | 0/3 | 0/4 | 0/4 | 1.34 |
| C2 | 6.636 | Quantitative type 1 | 0.02 | 0.9 | 20 | 0/3 | 0/4 | 0/4 | 0/3 | 0/4 | 0/4 | 3.68 |
| C3 | 0.132 | Quantitative type 1 | 0.01 | 0.9 | 4 | 0/3 | 0/4 | 0/4 | 0/3 | 0/4 | 0/4 | 9.32 |
| C3-DC (C4-OH) | 0.743 | Quantitative type 2 | 0.01 | - | - | 0/3 | 0/4 | 0/4 | - | - | - | 11.50 |
| C3-OH | - | Not validated | 0.01 | 0.1 | 1 | 0/3 | 0/4 | 0/4 | - | - | - | - |
| C3:1 | - | Not validated | 0.01 | - | - | 3/3 | 0/4 | 0/4 | - | - | - | 17.20 |
| C4 | 0.297 | Quantitative type 1 | 0.01 | 0.03 | 6 | 0/3 | 0/4 | 0/4 | 0/3 | 0/4 | 0/4 | 5.50 |
| C4:1 | - | Not validated | 0.01 | - | - | 0/3 | 0/4 | 0/4 | - | - | - | - |
| C5 | 0.285 | Quantitative type 1 | 0.01 | 0.03 | 6 | 0/3 | 0/4 | 0/4 | 0/3 | 0/4 | 0/4 | 4.08 |
| C5-DC (C6-OH) | 0.014 | Not validated | 0.01 | - | - | 0/3 | 0/4 | 0/4 | - | - | - | 0.00 |
| C5-M-DC | - | Not validated | 0.01 | - | - | 0/3 | 0/4 | 0/4 | - | - | - | 4.37 |
| C5-OH (C3-DC-M) | 1.723 | Quantitative type 2 | 0.01 | 0.05 | 1 | 0/3 | 0/4 | 0/4 | - | - | - | - |
| C5:1 | 0.363 | Not validated | 0.01 | 0.03 | 1 | 0/3 | 0/4 | 0/4 | - | - | - | - |
| C5:1-DC | - | Not validated | 0.01 | - | - | 0/3 | 0/4 | 0/4 | - | - | - | - |
| C6 (C4:1-DC) | - | Quantitative type 1 | 0.01 | 0.03 | 4 | 0/3 | 0/4 | 0/4 | 0/3 | 0/4 | 0/4 | 4.76 |
| C6:1 | - | Not validated | 0.01 | 0.03 | 1 | 0/3 | 0/4 | 0/4 | - | - | - | - |
| C7-DC | - | Quantitative type 2 | 0.01 | - | - | 0/3 | 0/4 | 0/4 | - | - | - | 16.48 |
| C8 | - | Quantitative type 1 | 0.01 | 0.2 | 4 | 0/3 | 0/4 | 0/4 | 0/3 | 0/4 | 0/4 | 5.55 |
| C9 | - | Quantitative type 2 | 0.01 | 0.05 | 1 | 0/3 | 0/4 | 0/4 | - | - | - | - |
| C10 | - | Quantitative type 1 | 0.01 | 0.1 | 3 | 0/3 | 0/4 | 0/4 | 0/3 | 0/4 | 0/4 | 4.11 |
| C10:1 | - | Not validated | 0.01 | - | - | 3/3 | 0/4 | 0/4 | - | - | - | 0.00 |
| C10:2 | - | Not validated | 0.01 | - | - | 0/3 | 0/4 | 0/4 | - | - | - | 10.90 |
| C12 | - | Quantitative type 1 | 0.01 | 0.8 | 7 | 0/3 | 0/4 | 0/4 | 0/3 | 0/4 | 0/4 | 3.49 |
| C12-DC | - | Not validated | 0.01 | - | - | 0/3 | 0/4 | 0/4 | - | - | - | 0.00 |
| C12:1 | - | Not validated | 0.01 | - | - | 0/3 | 0/4 | 0/4 | - | - | - | 0.00 |
| C14 | 0.032 | Quantitative type 1 | 0.01 | 0.024 | 6 | 0/3 | 0/4 | 0/4 | 0/3 | 0/4 | 0/4 | 5.51 |
| C14:1 | - | Quantitative type 2 | 0.01 | - | - | 0/3 | 0/4 | 0/4 | - | - | - | 5.33 |
| C14:1-OH | 0.049 | Quantitative type 2 | 0.01 | - | - | 0/3 | 0/4 | 0/4 | - | - | - | 10.20 |
| C14:2 | - | Quantitative type 2 | 0.01 | - | - | 0/3 | 0/4 | 0/4 | - | - | - | 10.61 |
| C14:2-OH | - | Not validated | 0.01 | - | - | 0/3 | 0/4 | 0/4 | - | - | - | - |
| C16 | 0.15 | Quantitative type 1 | 0.01 | 0.02 | 7 | 0/3 | 0/4 | 0/4 | 0/3 | 0/4 | 0/4 | 4.75 |
| C16-OH | 0.003 | Not validated | 0.01 | - | - | 0/3 | 0/4 | 0/4 | - | - | - | - |
| C16:1 | - | Quantitative type 2 | 0.01 | - | - | 0/3 | 0/4 | 0/4 | - | - | - | 10.94 |
| C16:1-OH | 0.147 | Quantitative type 2 | 0.01 | - | - | 0/3 | 0/4 | 0/4 | - | - | - | - |
| C16:2 | - | Not validated | 0.01 | - | - | 0/3 | 0/4 | 0/4 | - | - | - | - |
| C16:2-OH | - | Not validated | 0.01 | - | - | 0/3 | 0/4 | 0/4 | - | - | - | - |
| C18 | 0.552 | Quantitative type 1 | 0.01 | 0.015 | 6 | 0/3 | 0/4 | 0/4 | 0/3 | 0/4 | 0/4 | 4.98 |
| C18:1 | 0.16 | Quantitative type 2 | 0.01 | - | - | 0/3 | 0/4 | 0/4 | - | - | - | 6.63 |
| C18:1-OH | 0.016 | Not validated | 0.01 | - | - | 0/3 | 0/4 | 0/4 | - | - | - | 0.00 |
| C18:2 | 0.047 | Quantitative type 2 | 0.01 | - | - | 0/3 | 0/4 | 0/4 | - | - | - | 8.35 |
| Trigonelline | 1.891 | Quantitative type 2 | 0.1 | 0.3 | 500 | 0/3 | 0/4 | 0/4 | - | - | - | 4.76 |
| TMAO | - | Quantitative type 2 | 0.015 | 0.045 | 200 | 0/3 | 0/4 | 0/4 | - | - | - | 4.20 |
| Ala | - | Quantitative type 1 | 0.01 | 20 | 1600 | 0/3 | 0/4 | 0/4 | 0/3 | 0/4 | 0/4 | 2.37 |
| Arg | 352.139 | Quantitative type 1 | 0.1 | 5 | 400 | 0/3 | 0/4 | 0/4 | 0/3 | 0/4 | 0/4 | 3.71 |
| Asn | 333.144 | Quantitative type 1 | 0.06 | 5 | 400 | 0/3 | 0/4 | 0/4 | 0/3 | 0/4 | 0/4 | 2.06 |
| Asp | 188.706 | Quantitative type 1 | 0.1 | 5 | 400 | 0/3 | 0/4 | 0/4 | 0/3 | 0/4 | 0/4 | 3.70 |
| Cys | 564.175 | Quantitative type 2 | 0.01 | 0.03 | 80 | 0/3 | 0/4 | 0/4 | - | - | - | 11.43 |
| Gln | 1,910.852 | Quantitative type 1 | 0.05 | 20 | 1600 | 0/3 | 0/4 | 0/4 | 0/3 | 0/4 | 0/4 | 4.13 |
| Glu | 1,009.902 | Quantitative type 1 | 0.06 | 10 | 800 | 0/3 | 0/4 | 0/4 | 0/3 | 0/4 | 0/4 | 1.92 |
| Gly | 897.644 | Quantitative type 1 | 0.06 | 25 | 2000 | 0/3 | 0/4 | 0/4 | 0/3 | 0/4 | 0/4 | 5.55 |
| His | 224.411 | Quantitative type 1 | 0.01 | 5 | 400 | 0/3 | 0/4 | 0/4 | 0/3 | 0/4 | 0/4 | 4.59 |
| Ile | 744.434 | Quantitative type 1 | 0.1 | 5 | 400 | 0/3 | 0/4 | 0/4 | 0/3 | 0/4 | 0/4 | 3.66 |
| Leu | - | Quantitative type 1 | 0.2 | 10 | 400 | 0/3 | 0/4 | 0/4 | 0/3 | 0/4 | 0/4 | 3.48 |
| Lys | 436.549 | Quantitative type 1 | 0.15 | 10 | 800 | 0/3 | 0/4 | 0/4 | 0/3 | 0/4 | 0/4 | 1.32 |
| Met | 413.001 | Quantitative type 1 | 0.25 | 5 | 400 | 0/3 | 0/4 | 0/4 | 0/3 | 0/4 | 0/4 | 5.37 |
| Phe | - | Quantitative type 1 | 0.01 | 5 | 400 | 0/3 | 0/4 | 0/4 | 0/3 | 0/4 | 0/4 | 3.52 |
| Pro | 652.575 | Quantitative type 1 | 0.01 | 10 | 800 | 0/3 | 0/4 | 0/4 | 0/3 | 0/4 | 0/4 | 1.30 |
| Ser | - | Quantitative type 1 | 0.015 | 5 | 400 | 0/3 | 0/4 | 0/4 | 0/3 | 0/4 | 0/4 | 2.96 |
| Thr | 639.145 | Quantitative type 1 | 0.01 | 5 | 400 | 0/3 | 0/4 | 0/4 | 0/3 | 0/4 | 0/4 | 2.36 |
| Trp | 164.279 | Quantitative type 1 | 0.025 | 5 | 400 | 0/3 | 0/4 | 0/4 | 0/3 | 0/4 | 0/4 | 5.13 |
| Tyr | 477.713 | Quantitative type 1 | 0.006 | 5 | 400 | 0/3 | 0/4 | 0/4 | 0/3 | 0/4 | 0/4 | 4.26 |
| Val | 891.444 | Quantitative type 1 | 0.02 | 10 | 800 | 0/3 | 0/4 | 0/4 | 0/3 | 0/4 | 0/4 | 3.59 |
| 1-Met-His | 1.768 | Quantitative type 2 | 0.003 | 0.01 | 15 | 0/3 | 0/4 | 0/4 | - | - | - | 3.06 |
| 3-Met-His | 1.037 | Quantitative type 2 | 0.004 | 0.012 | 30 | 0/3 | 0/4 | 0/4 | - | - | - | 4.60 |
| 5-AVA | 0.978 | Quantitative type 2 | 0.01 | 0.03 | 50 | 0/3 | 0/4 | 0/4 | - | - | - | 5.48 |
| AABA | 8.112 | Quantitative type 2 | 0.01 | 0.03 | 30 | 0/3 | 0/4 | 0/4 | - | - | - | 2.40 |
| Ac-Orn | - | Quantitative type 1 | 0.005 | 0.5 | 40 | 0/3 | 0/4 | 0/4 | 0/3 | 0/4 | 0/4 | 3.43 |
| ADMA | 2.056 | Quantitative type 1 | 0.01 | 0.25 | 20 | 0/3 | 0/4 | 0/4 | 0/3 | 0/4 | 0/4 | 3.53 |
| alpha-AAA | 1.638 | Quantitative type 1 | 0.04 | 1 | 80 | 0/3 | 0/4 | 0/4 | 0/3 | 0/4 | 0/4 | 2.03 |
| Anserine | 0.403 | Quantitative type 2 | 0.005 | 0.6 | 25 | 0/3 | 0/4 | 0/4 | - | - | - | 1.59 |
| BABA | 0.033 | Quantitative type 2 | 0.005 | 0.1 | 100 | 0/3 | 0/4 | 0/4 | - | - | - | 8.90 |
| Betaine | 18.256 | Quantitative type 2 | 0.3 | 1 | 60 | 0/3 | 0/4 | 0/4 | - | - | - | 2.61 |
| c4-OH-Pro | - | Quantitative type 1 | 0.025 | 1 | 80 | 0/3 | 0/4 | 0/4 | 3/3 | 4/4 | 0/4 | - |
| Carnosine | 3.811 | Quantitative type 1 | 0.03 | 0.5 | 40 | 0/3 | 0/4 | 0/4 | 0/3 | 0/4 | 0/4 | 6.09 |
| Cit | 5.134 | Quantitative type 1 | 1 | 5 | 400 | 0/3 | 0/4 | 0/4 | 0/3 | 0/4 | 0/4 | 4.27 |
| Creatinine | 81.572 | Quantitative type 1 | 0.4 | 10 | 800 | 0/3 | 0/4 | 0/4 | 0/3 | 0/4 | 0/4 | 7.55 |
| Cystine | 32.447 | Quantitative type 2 | 0.005 | 0.015 | 200 | 0/3 | 0/4 | 0/4 | - | - | - | 16.37 |
| DOPA | 0.075 | Quantitative type 1 | 0.03 | 0.5 | 40 | 0/3 | 0/4 | 0/4 | 0/3 | 0/4 | 0/4 | 14.84 |
| HArg | 0.473 | Quantitative type 2 | 0.005 | 0.015 | 20 | 0/3 | 0/4 | 0/4 | - | - | - | 6.30 |
| HCys | 39.671 | Quantitative type 2 | 0.4 | 1.5 | 15000 | 0/3 | 0/4 | 0/4 | - | - | - | - |
| Kynurenine | 0.663 | Quantitative type 1 | 0.002 | 1 | 80 | 0/3 | 0/4 | 0/4 | 0/3 | 0/4 | 0/4 | 3.94 |
| Met-SO | 46.135 | Quantitative type 1 | 0.01 | 1 | 80 | 3/3 | 4/4 | 0/4 | 0/3 | 0/4 | 0/4 | - |
| Nitro-Tyr | - | Quantitative type 1 | 0.01 | 1 | 80 | 0/3 | 0/4 | 0/4 | 0/3 | 0/4 | 0/4 | 5.35 |
| Orn | 53.817 | Quantitative type 1 | 0.01 | 5 | 400 | 0/3 | 0/4 | 0/4 | 0/3 | 0/4 | 0/4 | 1.99 |
| PAG | - | Quantitative type 2 | 0.006 | 0.025 | 75 | 0/3 | 0/4 | 0/4 | - | - | - | 5.10 |
| PheAlaBetaine | 0.025 | Quantitative type 2 | 0.01 | 0.03 | 10 | 0/3 | 0/4 | 0/4 | - | - | - | 9.45 |
| ProBetaine | 1.114 | Quantitative type 2 | 0.01 | 0.04 | 35 | 0/3 | 0/4 | 0/4 | - | - | - | 5.11 |
| Sarcosine | 0.6 | Quantitative type 1 | 0.015 | 1 | 80 | 0/3 | 0/4 | 0/4 | 1/3 | 1/4 | 0/4 | - |
| SDMA | 0.927 | Quantitative type 1 | 0.002 | 0.1 | 8 | 0/3 | 0/4 | 0/4 | 0/3 | 0/4 | 0/4 | 5.29 |
| t4-OH-Pro | 10.056 | Quantitative type 1 | 0.02 | 1 | 80 | 0/3 | 0/4 | 0/4 | 0/3 | 0/4 | 0/4 | 3.70 |
| Taurine | - | Quantitative type 1 | 0.01 | 2.5 | 200 | 0/3 | 0/4 | 0/4 | 0/3 | 0/4 | 0/4 | 4.33 |
| TrpBetaine | 0.287 | Quantitative type 2 | 0.004 | 0.015 | 30 | 0/3 | 0/4 | 0/4 | - | - | - | 19.56 |
| CA | 0.036 | Quantitative type 2 | 0.002 | 0.005 | 45 | 0/3 | 0/4 | 0/4 | - | - | - | 11.33 |
| CDCA | 0.046 | Quantitative type 2 | 0.003 | 0.01 | 10 | 0/3 | 0/4 | 0/4 | - | - | - | 2.45 |
| DCA | 0.072 | Quantitative type 2 | 0.001 | 0.003 | 30 | 0/3 | 0/4 | 0/4 | - | - | - | 3.43 |
| GCA | 0.192 | Quantitative type 2 | 0.001 | 0.004 | 40 | 0/3 | 0/4 | 0/4 | - | - | - | 6.11 |
| GCDCA | 0.135 | Quantitative type 2 | 0 | 0.001 | 15 | 0/3 | 0/4 | 0/4 | - | - | - | 5.09 |
| GDCA | 0.099 | Quantitative type 2 | 0.002 | 0.005 | 30 | 0/3 | 0/4 | 0/4 | - | - | - | 6.03 |
| GLCA | 0.005 | Quantitative type 2 | 0 | 0.001 | 20 | 0/3 | 0/4 | 0/4 | - | - | - | 5.22 |
| GLCAS | 0.016 | Quantitative type 2 | 0 | 0 | 2.5 | 3/3 | 4/4 | 4/4 | - | - | - | - |
| GUDCA | 0.01 | Quantitative type 2 | 0.001 | 0.003 | 30 | 0/3 | 0/4 | 0/4 | - | - | - | 6.44 |
| TCA | 0.047 | Quantitative type 2 | 0.001 | 0.003 | 35 | 0/3 | 0/4 | 0/4 | - | - | - | 2.68 |
| TCDCA | 0.04 | Quantitative type 2 | 0 | 0.001 | 5 | 0/3 | 0/4 | 0/4 | - | - | - | 10.10 |
| TDCA | 0.02 | Quantitative type 2 | 0.001 | 0.004 | 30 | 0/3 | 0/4 | 0/4 | - | - | - | 5.78 |
| TLCA | - | Quantitative type 2 | 0.001 | 0.003 | 15 | 0/3 | 0/4 | 0/4 | - | - | - | 5.56 |
| TMCA | - | Quantitative type 2 | 0.003 | 0.01 | 50 | 0/3 | 0/4 | 0/4 | - | - | - | 4.22 |
| beta-Ala | 10.126 | Quantitative type 2 | 0.01 | 0.04 | 80 | 0/3 | 0/4 | 0/4 | - | - | - | 12.91 |
| Dopamine | - | Quantitative type 1 | 0.002 | 1 | 80 | 0/3 | 0/4 | 0/4 | 0/3 | 0/4 | 0/4 | 11.79 |
| GABA | 15.671 | Quantitative type 2 | 0.005 | 0.015 | 80 | 0/3 | 0/4 | 0/4 | - | - | - | 5.86 |
| Histamine | 7.543 | Quantitative type 1 | 0.01 | 1 | 80 | 0/3 | 4/4 | 0/4 | 0/3 | 0/4 | 0/4 | - |
| PEA | - | Quantitative type 1 | 0.001 | 0.1 | 8 | 0/3 | 0/4 | 0/4 | 0/3 | 0/4 | 3/4 | 9.49 |
| Putrescine | 1.022 | Quantitative type 1 | 0.001 | 0.05 | 8 | 0/3 | 0/4 | 0/4 | 0/3 | 0/4 | 0/4 | 2.61 |
| Serotonin | - | Quantitative type 1 | 0.001 | 0.1 | 8 | 0/3 | 0/4 | 0/4 | 0/3 | 0/4 | 0/4 | 6.82 |
| Spermidine | 0.997 | Quantitative type 1 | 0.002 | 0.25 | 20 | 0/3 | 0/4 | 0/4 | 0/3 | 0/4 | 0/4 | 7.57 |
| Spermine | 0.761 | Quantitative type 1 | 0.001 | 0.25 | 20 | 0/3 | 0/4 | 0/4 | 0/3 | 0/4 | 0/4 | 7.28 |
| AconAcid | - | Quantitative type 2 | 0.2 | 1 | 300 | 0/3 | 0/4 | 0/4 | - | - | - | 8.17 |
| DiCA(12:0) | - | Quantitative type 2 | 0.01 | 0.04 | 15 | 0/3 | 0/4 | 0/4 | - | - | - | 15.27 |
| DiCA(14:0) | - | Quantitative type 2 | 0.002 | 0.005 | 15 | 0/3 | 0/4 | 0/4 | - | - | - | 8.75 |
| HipAcid | 0.751 | Quantitative type 2 | 0.01 | 0.025 | 200 | 0/3 | 0/4 | 0/4 | - | - | - | 9.29 |
| Lac | 12,416.750 | Quantitative type 2 | 1.5 | 5 | 2000 | 0/3 | 0/4 | 0/4 | - | - | - | - |
| OH-GlutAcid | 20.678 | Quantitative type 2 | 0.15 | 0.5 | 600 | 0/3 | 0/4 | 0/4 | - | - | - | 7.02 |
| Suc | 122.798 | Quantitative type 2 | 0.2 | 1 | 750 | 0/3 | 0/4 | 0/4 | - | - | - | 2.80 |
| Cer d16:1/18:0 | 0.773 | Quantitative type 2 | 0.05 | 0.1 | 1 | 0/3 | 0/4 | 0/4 | - | - | - | - |
| Cer d16:1/20:0 | 0.435 | Quantitative type 2 | 0.031 | 0.1 | 1 | 0/3 | 0/4 | 0/4 | - | - | - | - |
| Cer d16:1/22:0 | 0.569 | Quantitative type 2 | 0.053 | - | - | 0/3 | 0/4 | 3/4 | - | - | - | 12.97 |
| Cer d16:1/23:0 | 0.113 | Quantitative type 2 | 0.025 | - | - | 0/3 | 0/4 | 0/4 | - | - | - | 26.04 |
| Cer d16:1/24:0 | 0.074 | Quantitative type 2 | 0.023 | - | - | 0/3 | 0/4 | 0/4 | - | - | - | 18.23 |
| Cer d18:1/14:0 | 0.5 | Quantitative type 2 | 0.058 | 0.06 | 10 | 0/3 | 0/4 | 0/4 | - | - | - | - |
| Cer d18:1/16:0 | 1.974 | Quantitative type 2 | 0.017 | - | - | 0/3 | 0/4 | 0/4 | - | - | - | 9.31 |
| Cer d18:1/18:0 | 3.01 | Quantitative type 2 | 0.021 | - | - | 0/3 | 0/4 | 0/4 | - | - | - | 26.05 |
| Cer d18:1/18:0-OH | - | Quantitative type 2 | 0.111 | 1.25 | 10 | 0/3 | 0/4 | 0/4 | - | - | - | - |
| Cer d18:1/18:1 | 0.343 | Quantitative type 2 | 0.018 | - | - | 0/3 | 0/4 | 0/4 | - | - | - | 9.69 |
| Cer d18:1/20:0 | 0.664 | Quantitative type 2 | 0.013 | - | - | 0/3 | 0/4 | 3/4 | - | - | - | 16.73 |
| Cer d18:1/20:0-OH | 1.108 | Quantitative type 2 | 1.03 | - | - | 0/3 | 0/4 | 0/4 | - | - | - | - |
| Cer d18:1/22:0 | 1.527 | Quantitative type 2 | 0.023 | - | - | 0/3 | 0/4 | 0/4 | - | - | - | 14.72 |
| Cer d18:1/23:0 | 0.662 | Quantitative type 2 | 0.02 | 0.05 | 1 | 0/3 | 0/4 | 0/4 | - | - | - | - |
| Cer d18:1/24:0 | 1.359 | Quantitative type 2 | 0.032 | - | - | 0/3 | 0/4 | 0/4 | - | - | - | 12.77 |
| Cer d18:1/24:1 | 4.287 | Quantitative type 2 | 0.017 | - | - | 0/3 | 0/4 | 0/4 | - | - | - | 12.79 |
| Cer d18:1/25:0 | 0.157 | Quantitative type 2 | 0.096 | - | - | 0/3 | 0/4 | 0/4 | - | - | - | 24.60 |
| Cer d18:1/26:0 | 0.073 | Quantitative type 2 | 0.03 | - | - | 0/3 | 0/4 | 0/4 | - | - | - | - |
| Cer d18:1/26:1 | 0.067 | Quantitative type 2 | 0.024 | - | - | 0/3 | 0/4 | 0/4 | - | - | - | - |
| Cer d18:2/14:0 | 0.043 | Quantitative type 2 | 0.006 | 0.01 | 0.2 | 0/3 | 0/4 | 0/4 | - | - | - | - |
| Cer d18:2/16:0 | 0.65 | Quantitative type 2 | 0.015 | - | - | 0/3 | 0/4 | 3/4 | - | - | - | 26.60 |
| Cer d18:2/18:0 | 1.473 | Quantitative type 2 | 0.014 | - | - | 0/3 | 0/4 | 3/4 | - | - | - | 21.68 |
| Cer d18:2/18:1 | 0.232 | Quantitative type 2 | 0.005 | 0.012 | 0.2 | 0/3 | 0/4 | 0/4 | - | - | - | - |
| Cer d18:2/20:0 | 0.89 | Quantitative type 2 | 0.017 | - | - | 0/3 | 0/4 | 0/4 | - | - | - | - |
| Cer d18:2/22:0 | 1.051 | Quantitative type 2 | 0.03 | - | - | 0/3 | 0/4 | 0/4 | - | - | - | 29.58 |
| Cer d18:2/23:0 | 0.177 | Quantitative type 2 | 0.016 | - | - | 0/3 | 0/4 | 0/4 | - | - | - | 22.34 |
| Cer d18:2/24:0 | 0.57 | Quantitative type 2 | 0.012 | - | - | 0/3 | 0/4 | 0/4 | - | - | - | 10.91 |
| Cer d18:2/24:1 | 0.861 | Quantitative type 2 | 0.011 | - | - | 0/3 | 0/4 | 3/4 | - | - | - | 17.54 |
| CE 14:0 | 4.141 | Quantitative type 2 | 1 | 2.5 | 20 | 0/3 | 0/4 | 3/4 | - | - | - | - |
| CE 14:1 | - | Quantitative type 2 | 0.1 | 0.13 | 0.5 | 3/3 | 0/4 | 3/4 | - | - | - | - |
| CE 15:0 | 3.053 | Quantitative type 2 | 0.1 | 0.7 | 7 | 0/3 | 0/4 | 3/4 | - | - | - | 9.41 |
| CE 15:1 | 0.723 | Quantitative type 2 | 0.1 | 0.15 | 1.5 | 0/3 | 4/4 | 3/4 | - | - | - | - |
| CE 16:0 | - | Quantitative type 2 | 1 | 40 | 400 | 3/3 | 4/4 | 3/4 | - | - | - | - |
| CE 16:1 | - | Quantitative type 2 | 1 | 35 | 110 | 0/3 | 4/4 | 3/4 | - | - | - | - |
| CE 17:0 | - | Quantitative type 2 | 1 | 3 | 12 | 0/3 | 0/4 | 3/4 | - | - | - | - |
| CE 17:1 | - | Quantitative type 2 | 0.1 | 2 | 6 | 0/3 | 0/4 | 3/4 | - | - | - | 4.54 |
| CE 18:0 | - | Quantitative type 2 | 1 | 2 | 20 | 0/3 | 0/4 | 2/4 | - | - | - | - |
| CE 18:1 | - | Quantitative type 2 | 1 | 60 | 500 | 0/3 | 0/4 | 3/4 | - | - | - | - |
| CE 18:2 | - | Quantitative type 2 | 1 | 300 | 2700 | 3/3 | 0/4 | 3/4 | - | - | - | - |
| CE 18:3 | - | Quantitative type 2 | 1 | 12 | 100 | 3/3 | 0/4 | 3/4 | - | - | - | - |
| CE 20:0 | - | Quantitative type 2 | 1 | 20 | 150 | 3/3 | 0/4 | 3/4 | - | - | - | - |
| CE 20:1 | - | Quantitative type 2 | 1 | 6 | 50 | 3/3 | 0/4 | 3/4 | - | - | - | - |
| CE 20:3 | - | Quantitative type 2 | 1 | 5 | 40 | 0/3 | 0/4 | 3/4 | - | - | - | - |
| CE 20:4 | - | Quantitative type 2 | 1 | 60 | 500 | 3/3 | 0/4 | 3/4 | - | - | - | - |
| CE 20:5 | - | Quantitative type 2 | 1 | 7 | 64 | 0/3 | 0/4 | 3/4 | - | - | - | 23.05 |
| CE 22:0 | - | Not validated | 1 | 2 | 10 | 3/3 | 0/4 | 3/4 | - | - | - | - |
| CE 22:1 | - | Quantitative type 2 | 0.1 | 0.2 | 1.2 | 0/3 | 0/4 | 2/4 | - | - | - | - |
| CE 22:2 | - | Quantitative type 2 | 0.1 | 0.2 | 2.2 | 3/3 | 0/4 | 3/4 | - | - | - | - |
| CE 22:5 | - | Quantitative type 2 | 0.1 | 0.6 | 5 | 0/3 | 0/4 | 3/4 | - | - | - | 20.52 |
| CE 22:6 | - | Quantitative type 2 | 0.1 | 3 | 40 | 3/3 | 0/4 | 3/4 | - | - | - | 7.55 |
| p-Cresol-SO4 | 1.476 | Quantitative type 2 | 0.03 | 0.1 | 50 | 0/3 | 0/4 | 0/4 | - | - | - | 4.92 |
| DG 14:0_14:0 | - | Not validated | 0.01 | 2.1 | 5 | 0/3 | 4/4 | 0/4 | - | - | - | - |
| DG 14:0_18:1 | 1.177 | Quantitative type 2 | 1 | - | - | 0/3 | 0/4 | 0/4 | - | - | - | 14.18 |
| DG 14:0_18:2 | 3.16 | Quantitative type 2 | 0.01 | - | - | 0/3 | 0/4 | 0/4 | - | - | - | - |
| DG 14:0_20:0 | - | Not validated | 0.01 | - | - | 3/3 | 4/4 | 3/4 | - | - | - | - |
| DG 14:1_18:1 | 0.193 | Not validated | 0.01 | - | - | 0/3 | 0/4 | 0/4 | - | - | - | - |
| DG 14:1_20:2 | - | Not validated | 0.01 | 1 | 5 | 1/3 | 0/4 | 0/4 | - | - | - | - |
| DG 16:0_16:0 | 0.002 | Not validated | 1 | - | - | 0/3 | 0/4 | 0/4 | - | - | - | - |
| DG 16:0_16:1 | 1.818 | Quantitative type 2 | 0.01 | - | - | 0/3 | 4/4 | 0/4 | - | - | - | - |
| DG 16:0_18:1 | 16 | Quantitative type 2 | 0.01 | - | - | 3/3 | 0/4 | 0/4 | - | - | - | 24.12 |
| DG 16:0_18:2 | 6.774 | Quantitative type 2 | 0.01 | - | - | 0/3 | 4/4 | 0/4 | - | - | - | - |
| DG 16:0_20:0 | - | Not validated | 0.01 | - | - | 0/3 | 1/4 | 0/4 | - | - | - | - |
| DG 16:0_20:3 | - | Not validated | 0.01 | - | - | 0/3 | 0/4 | 0/4 | - | - | - | - |
| DG 16:0_20:4 | - | Not validated | 0.01 | - | - | 1/3 | 0/4 | 2/4 | - | - | - | - |
| DG 16:1_18:0 | 0.395 | Quantitative type 2 | 0.01 | 0.2 | 1 | 0/3 | 0/4 | 0/4 | - | - | - | - |
| DG 16:1_18:1 | - | Not validated | 1 | - | - | 3/3 | 4/4 | 3/4 | - | - | - | - |
| DG 16:1_18:2 | 0.952 | Quantitative type 2 | 0.01 | - | - | 0/3 | 4/4 | 0/4 | - | - | - | - |
| DG 16:1_20:0 | - | Not validated | 0.01 | - | - | 0/3 | 1/4 | 0/4 | - | - | - | - |
| DG 17:0_17:1 | - | Not validated | 0.01 | - | - | 1/3 | 2/4 | 2/4 | - | - | - | - |
| DG 17:0_18:1 | 1.26 | Quantitative type 2 | 0.01 | 0.3 | 3 | 2/3 | 2/4 | 1/4 | - | - | - | - |
| DG 18:0_20:0 | - | Not validated | 1 | - | - | 0/3 | 0/4 | 0/4 | - | - | - | - |
| DG 18:0_20:4 | 2.928 | Not validated | 0.01 | - | - | 0/3 | 0/4 | 1/4 | - | - | - | - |
| DG 18:1_18:1 | 11.472 | Quantitative type 2 | 0.01 | - | - | 0/3 | 3/4 | 0/4 | - | - | - | - |
| DG 18:1_18:2 | 13.269 | Quantitative type 2 | 0.01 | - | - | 0/3 | 3/4 | 0/4 | - | - | - | - |
| DG 18:1_18:3 | 0.406 | Not validated | 0.01 | - | - | 0/3 | 0/4 | 0/4 | - | - | - | - |
| DG 18:1_18:4 | - | Not validated | 0.01 | - | - | 0/3 | 0/4 | 0/4 | - | - | - | - |
| DG 18:1_20:0 | 0.358 | Quantitative type 2 | 0.01 | - | - | 0/3 | 1/4 | 0/4 | - | - | - | - |
| DG 18:1_20:1 | 0.081 | Quantitative type 2 | 0.01 | 0.1 | 1 | 0/3 | 0/4 | 0/4 | - | - | - | - |
| DG 18:1_20:2 | - | Quantitative type 2 | 0.01 | - | - | 0/3 | 0/4 | 0/4 | - | - | - | - |
| DG 18:1_20:3 | - | Quantitative type 2 | 0.01 | - | - | 0/3 | 0/4 | 0/4 | - | - | - | - |
| DG 18:1_20:4 | 0.272 | Not validated | 0.01 | - | - | 0/3 | 0/4 | 0/4 | - | - | - | - |
| DG 18:1_22:5 | - | Not validated | 0.01 | - | - | 0/3 | 0/4 | 0/4 | - | - | - | - |
| DG 18:1_22:6 | - | Not validated | 0.01 | - | - | 3/3 | 4/4 | 3/4 | - | - | - | - |
| DG 18:2_18:2 | 2.412 | Quantitative type 2 | 0.01 | - | - | 0/3 | 4/4 | 0/4 | - | - | - | - |
| DG 18:2_18:3 | - | Not validated | 0.01 | 2 | 5 | 0/3 | 0/4 | 0/4 | - | - | - | - |
| DG 18:2_18:4 | - | Not validated | 0.01 | 1 | 5 | 0/3 | 0/4 | 1/4 | - | - | - | - |
| DG 18:2_20:0 | - | Quantitative type 2 | 0.01 | - | - | 0/3 | 0/4 | 0/4 | - | - | - | - |
| DG 18:2_20:4 | - | Not validated | 0.01 | - | - | 1/3 | 0/4 | 2/4 | - | - | - | - |
| DG 18:3_18:3 | - | Not validated | 0.01 | 1 | 5 | 0/3 | 0/4 | 1/4 | - | - | - | - |
| DG 18:3_20:2 | - | Not validated | 0.01 | - | - | 0/3 | 1/4 | 0/4 | - | - | - | - |
| DG 21:0_22:6 | - | Not validated | 0.01 | - | - | 0/3 | 0/4 | 0/4 | - | - | - | - |
| DG 22:1_22:2 | - | Not validated | 0.01 | - | - | 0/3 | 0/4 | 0/4 | - | - | - | - |
| DG O-14:0_18:2 | - | Not validated | 1 | 7 | 10 | 0/3 | 0/4 | 0/4 | - | - | - | - |
| DG O-16:0_18:1 | - | Not validated | 0.01 | 0.2 | 1 | 0/3 | 0/4 | 0/4 | - | - | - | - |
| DG O-18:2_18:2 | - | Quantitative type 2 | 0.01 | 0.15 | 1 | 0/3 | 0/4 | 0/4 | - | - | - | 27.94 |
| Cer d18:0/18:0 | 0.055 | Quantitative type 2 | 0.01 | 0.1 | 1 | 0/3 | 0/4 | 0/4 | - | - | - | - |
| Cer d18:0/18:0-OH | - | Quantitative type 2 | 0.765 | 0.9 | 14 | 0/3 | 0/4 | 0/4 | - | - | - | - |
| Cer d18:0/20:0 | - | Not validated | 0.01 | 0.1 | 1 | 0/3 | 0/4 | 0/4 | - | - | - | - |
| Cer d18:0/22:0 | - | Quantitative type 2 | 0.01 | 0.1 | 1 | 0/3 | 0/4 | 1/4 | - | - | - | - |
| Cer d18:0/24:0 | - | Quantitative type 2 | 0.01 | 0.2 | 10 | 0/3 | 0/4 | 0/4 | - | - | - | - |
| Cer d18:0/24:1 | - | Quantitative type 2 | 0.01 | - | - | 0/3 | 0/4 | 0/4 | - | - | - | - |
| Cer d18:0/26:1 | - | Not validated | 0.01 | - | - | 0/3 | 0/4 | 0/4 | - | - | - | - |
| Cer d18:0/26:1-OH | - | Not validated | 0.33 | - | - | 3/3 | 0/4 | 0/4 | - | - | - | - |
| FA 12:0 | - | Quantitative type 2 | 0.01 | 1 | 1000 | 0/3 | 0/4 | 0/4 | - | - | - | 11.68 |
| FA 14:0 | - | Quantitative type 2 | 0.2 | 0.8 | 700 | 0/3 | 0/4 | 0/4 | - | - | - | - |
| FA 16:0 | 952.172 | Not validated | 350 | 400 | 1200 | 0/3 | 0/4 | 0/4 | - | - | - | 3.03 |
| FA 18:0 | 942.272 | Not validated | 350 | 400 | 1500 | 0/3 | 0/4 | 0/4 | - | - | - | - |
| FA 18:1 | 643.591 | Quantitative type 2 | 0.1 | 0.4 | 800 | 0/3 | 0/4 | 0/4 | - | - | - | 5.12 |
| FA 18:2 | 82.139 | Quantitative type 2 | 0.03 | 0.1 | 150 | 0/3 | 0/4 | 0/4 | - | - | - | - |
| FA 20:1 | 30.274 | Quantitative type 2 | 0.03 | 0.1 | 450 | 0/3 | 0/4 | 0/4 | - | - | - | 24.16 |
| FA 20:2 | 24.933 | Quantitative type 2 | 0.1 | 0.3 | 1200 | 0/3 | 0/4 | 0/4 | - | - | - | 9.09 |
| FA 20:3 | 37.271 | Quantitative type 2 | 0.1 | 0.3 | 1500 | 0/3 | 0/4 | 0/4 | - | - | - | 18.29 |
| FA 20:4n-6 (AA) | 292.036 | Quantitative type 2 | 0.04 | 0.14 | 400 | 0/3 | 0/4 | 0/4 | - | - | - | 14.44 |
| FA 20:5n-3 (EPA) | 29.857 | Quantitative type 2 | 0.01 | 0.03 | 65 | 0/3 | 0/4 | 0/4 | - | - | - | 3.51 |
| FA 22:6n-3 (DHA) | 265.028 | Quantitative type 2 | 0.015 | 0.05 | 100 | 0/3 | 0/4 | 0/4 | - | - | - | 6.80 |
| Hex2Cer d18:1/14:0 | 0.1 | Quantitative type 2 | 0.01 | - | - | 0/3 | 2/4 | 0/4 | - | - | - | - |
| Hex2Cer d18:1/16:0 | 0.787 | Quantitative type 2 | 0.01 | - | - | 3/3 | 0/4 | 0/4 | - | - | - | 8.99 |
| Hex2Cer d18:1/18:0 | 0.644 | Quantitative type 2 | 0.01 | - | - | 0/3 | 0/4 | 0/4 | - | - | - | - |
| Hex2Cer d18:1/20:0 | 0.264 | Quantitative type 2 | 0.01 | - | - | 0/3 | 0/4 | 0/4 | - | - | - | - |
| Hex2Cer d18:1/22:0 | 0.672 | Quantitative type 2 | 0.01 | - | - | 0/3 | 0/4 | 0/4 | - | - | - | - |
| Hex2Cer d18:1/24:0 | 0.497 | Quantitative type 2 | 0.01 | - | - | 0/3 | 0/4 | 0/4 | - | - | - | - |
| Hex2Cer d18:1/24:1 | 0.588 | Quantitative type 2 | 0.01 | - | - | 0/3 | 4/4 | 0/4 | - | - | - | - |
| Hex2Cer d18:1/26:0 | - | Quantitative type 2 | 0.01 | 0.02 | 1.5 | 0/3 | 0/4 | 0/4 | - | - | - | - |
| Hex2Cer d18:1/26:1 | - | Quantitative type 2 | 0.01 | 0.012 | 1.5 | 0/3 | 0/4 | 0/4 | - | - | - | - |
| Hex3Cer d18:1/16:0 | 1.761 | Quantitative type 2 | 0.01 | - | - | 3/3 | 0/4 | 0/4 | - | - | - | 19.17 |
| Hex3Cer d18:1/18:0 | 1.08 | Quantitative type 2 | 0.01 | 0.1 | 1 | 0/3 | 1/4 | 0/4 | - | - | - | - |
| Hex3Cer d18:1/20:0 | 0.177 | Quantitative type 2 | 0.01 | - | - | 0/3 | 0/4 | 0/4 | - | - | - | - |
| Hex3Cer d18:1/22:0 | 2.173 | Quantitative type 2 | 0.01 | - | - | 0/3 | 4/4 | 0/4 | - | - | - | - |
| Hex3Cer d18:1/24:1 | 4.691 | Quantitative type 2 | 0.01 | - | - | 1/3 | 3/4 | 0/4 | - | - | - | - |
| Hex3Cer d18:1/26:1 | - | Quantitative type 2 | 0.01 | 0.35 | 5 | 0/3 | 0/4 | 0/4 | - | - | - | - |
| Hex-Cer d16:1/20:0 | - | Quantitative type 2 | 0.01 | 0.012 | 0.2 | 0/3 | 0/4 | 0/4 | - | - | - | - |
| Hex-Cer d16:1/22:0 | - | Quantitative type 2 | 0.01 | 0.02 | 0.2 | 0/3 | 1/4 | 0/4 | - | - | - | - |
| Hex-Cer d16:1/24:0 | - | Quantitative type 2 | 0.01 | 0.1 | 0.5 | 0/3 | 1/4 | 0/4 | - | - | - | - |
| Hex-Cer d18:1/14:0 | - | Quantitative type 2 | 0.01 | 0.06 | 4 | 0/3 | 0/4 | 0/4 | - | - | - | - |
| Hex-Cer d18:1/16:0 | 0.091 | Quantitative type 2 | 0.01 | 0.49 | 11 | 3/3 | 4/4 | 0/4 | - | - | - | - |
| Hex-Cer d18:1/18:0 | 0.09 | Quantitative type 2 | 0.01 | 0.09 | 10 | 0/3 | 0/4 | 0/4 | - | - | - | - |
| Hex-Cer d18:1/18:1 | - | Quantitative type 2 | 0.01 | - | - | 2/3 | 0/4 | 0/4 | - | - | - | 22.66 |
| Hex-Cer d18:1/20:0 | - | Quantitative type 2 | 0.01 | 0.1 | 1 | 0/3 | 0/4 | 0/4 | - | - | - | - |
| Hex-Cer d18:1/22:0 | 0.361 | Quantitative type 2 | 0.01 | - | - | 3/3 | 0/4 | 0/4 | - | - | - | 11.59 |
| Hex-Cer d18:1/23:0 | 0.156 | Quantitative type 2 | 0.01 | - | - | 0/3 | 0/4 | 0/4 | - | - | - | 28.56 |
| Hex-Cer d18:1/24:0 | 0.207 | Quantitative type 2 | 0.01 | - | - | 0/3 | 0/4 | 0/4 | - | - | - | 21.62 |
| Hex-Cer d18:1/24:1 | 0.926 | Quantitative type 2 | 0.01 | - | - | 3/3 | 0/4 | 0/4 | - | - | - | 10.76 |
| Hex-Cer d18:1/26:0 | - | Quantitative type 2 | 0.01 | 0.2 | 1 | 0/3 | 1/4 | 0/4 | - | - | - | - |
| Hex-Cer d18:1/26:1 | - | Quantitative type 2 | 0.01 | 0.2 | 1 | 0/3 | 0/4 | 0/4 | - | - | - | - |
| Hex-Cer d18:2/16:0 | - | Quantitative type 2 | 0.01 | 0.05 | 1 | 0/3 | 0/4 | 0/4 | - | - | - | - |
| Hex-Cer d18:2/18:0 | - | Quantitative type 2 | 0.01 | 0.05 | 1 | 0/3 | 0/4 | 0/4 | - | - | - | - |
| Hex-Cer d18:2/20:0 | - | Quantitative type 2 | 0.01 | 0.05 | 1 | 0/3 | 0/4 | 0/4 | - | - | - | - |
| Hex-Cer d18:2/22:0 | - | Quantitative type 2 | 0.01 | - | - | 0/3 | 1/4 | 0/4 | - | - | - | - |
| Hex-Cer d18:2/23:0 | - | Quantitative type 2 | 0.01 | - | - | 0/3 | 2/4 | 0/4 | - | - | - | - |
| Hex-Cer d18:2/24:0 | - | Quantitative type 2 | 0.01 | - | - | 2/3 | 3/4 | 0/4 | - | - | - | - |
| AbsAcid | - | Quantitative type 2 | 0 | 0.1 | 20 | 0/3 | 0/4 | 0/4 | - | - | - | - |
| Cortisol | 0.026 | Quantitative type 2 | 0.005 | 0.015 | 50 | 0/3 | 0/4 | 0/4 | - | - | - | 3.76 |
| Cortisone | - | Quantitative type 2 | 0.005 | 0.3 | 30 | 0/3 | 0/4 | 0/4 | - | - | - | - |
| DHEAS | 0.109 | Quantitative type 2 | 0.015 | 0.05 | 1500 | 0/3 | 0/4 | 0/4 | - | - | - | 9.86 |
| 3-IAA | 0.148 | Quantitative type 2 | 0.01 | 0.03 | 100 | 0/3 | 0/4 | 0/4 | - | - | - | 4.12 |
| 3-IPA | 0.017 | Quantitative type 2 | 0.003 | 0.01 | 25 | 0/3 | 0/4 | 0/4 | - | - | - | 3.42 |
| Ind-SO4 | 0.176 | Quantitative type 2 | 0.05 | 0.15 | 40 | 0/3 | 0/4 | 0/4 | - | - | - | 4.24 |
| Indole | - | Quantitative type 2 | 4 | 50 | 15000 | 0/3 | 0/4 | 0/4 | - | - | - | 11.80 |
| Hypoxanthine | 837.532 | Quantitative type 2 | 0.1 | 0.3 | 250 | 0/3 | 0/4 | 0/4 | - | - | - | 7.22 |
| Xanthine | 72.004 | Quantitative type 2 | 0.4 | 1.2 | 300 | 0/3 | 0/4 | 0/4 | - | - | - | 6.22 |
| LPC 14:0 | 6.423 | Not validated | 1 | 15 | 200 | 0/3 | 0/4 | 0/4 | - | - | - | - |
| LPC 16:0 | 65.799 | Quantitative type 2 | 0.01 | - | - | 0/3 | 0/4 | 0/4 | - | - | - | 6.06 |
| LPC 16:1 | 2.77 | Quantitative type 2 | 0.01 | - | - | 0/3 | 0/4 | 0/4 | - | - | - | 7.63 |
| LPC 17:0 | 1.32 | Quantitative type 2 | 0.01 | - | - | 0/3 | 0/4 | 0/4 | - | - | - | 8.68 |
| LPC 18:0 | 16.564 | Quantitative type 2 | 0.01 | 2 | 250 | 0/3 | 0/4 | 0/4 | - | - | - | 9.72 |
| LPC 18:1 | 22.943 | Quantitative type 2 | 0.01 | - | - | 0/3 | 0/4 | 0/4 | - | - | - | 10.15 |
| LPC 18:2 | 22.162 | Quantitative type 2 | 0.01 | - | - | 0/3 | 0/4 | 0/4 | - | - | - | 8.74 |
| LPC 20:3 | 1.759 | Quantitative type 2 | 0.01 | - | - | 0/3 | 0/4 | 0/4 | - | - | - | 12.82 |
| LPC 20:4 | 30.696 | Quantitative type 2 | 0.01 | - | - | 0/3 | 0/4 | 0/4 | - | - | - | 10.78 |
| LPC 24:0 | 0.071 | Quantitative type 2 | 0.01 | - | - | 0/3 | 0/4 | 3/4 | - | - | - | 25.15 |
| LPC 26:0 | 0.059 | Quantitative type 2 | 0.01 | - | - | 0/3 | 4/4 | 3/4 | - | - | - | - |
| LPC 26:1 | 0.18 | Quantitative type 2 | 0.01 | 0.2 | 1 | 0/3 | 4/4 | 3/4 | - | - | - | - |
| PC 24:0 | 0.075 | Quantitative type 2 | 0.01 | 10 | 200 | 0/3 | 0/4 | 0/4 | - | - | - | 5.60 |
| PC 26:0 | - | Not validated | 1 | 3 | 50 | 0/3 | 0/4 | 0/4 | - | - | - | - |
| PC 28:1 | 0.896 | Quantitative type 2 | 0.01 | - | - | 0/3 | 0/4 | 0/4 | - | - | - | 6.28 |
| PC 30:0 | 11.392 | Quantitative type 2 | 0.01 | - | - | 0/3 | 0/4 | 0/4 | - | - | - | 2.84 |
| PC 32:0 | 82.711 | Quantitative type 2 | 0.01 | - | - | 0/3 | 0/4 | 0/4 | - | - | - | 8.78 |
| PC 32:1 | 14.123 | Quantitative type 2 | 0.01 | - | - | 0/3 | 0/4 | 0/4 | - | - | - | 9.21 |
| PC 32:2 | 2.694 | Quantitative type 2 | 0.01 | - | - | 0/3 | 0/4 | 0/4 | - | - | - | 10.06 |
| PC 32:3 | 0.42 | Quantitative type 2 | 0.01 | - | - | 0/3 | 0/4 | 0/4 | - | - | - | 11.97 |
| PC 34:1 | 196.72 | Quantitative type 2 | 0.01 | - | - | 0/3 | 0/4 | 0/4 | - | - | - | 7.93 |
| PC 34:2 | 112.986 | Quantitative type 2 | 0.01 | - | - | 0/3 | 0/4 | 0/4 | - | - | - | 8.25 |
| PC 34:3 | 7.835 | Quantitative type 2 | 0.01 | - | - | 0/3 | 0/4 | 0/4 | - | - | - | 8.87 |
| PC 34:4 | 1.696 | Quantitative type 2 | 0.01 | - | - | 0/3 | 0/4 | 0/4 | - | - | - | 7.71 |
| PC 36:0 | 17.753 | Quantitative type 2 | 0.01 | - | - | 0/3 | 0/4 | 3/4 | - | - | - | 10.61 |
| PC 36:1 | 30.696 | Quantitative type 2 | 0.01 | - | - | 0/3 | 0/4 | 0/4 | - | - | - | 5.89 |
| PC 36:2 | 50.914 | Quantitative type 2 | 0.01 | - | - | 0/3 | 0/4 | 0/4 | - | - | - | 7.02 |
| PC 36:3 | 28.641 | Quantitative type 2 | 0.01 | - | - | 0/3 | 0/4 | 0/4 | - | - | - | 6.95 |
| PC 36:4 | 84.449 | Quantitative type 2 | 0.01 | - | - | 0/3 | 0/4 | 0/4 | - | - | - | 6.76 |
| PC 36:5 | 12.042 | Quantitative type 2 | 0.01 | - | - | 0/3 | 0/4 | 0/4 | - | - | - | 6.57 |
| PC 36:6 | 0.941 | Quantitative type 2 | 0.01 | - | - | 0/3 | 0/4 | 0/4 | - | - | - | 7.32 |
| PC 38:0 | 8.877 | Quantitative type 2 | 0.01 | - | - | 0/3 | 0/4 | 0/4 | - | - | - | 6.23 |
| PC 38:3 | 6.681 | Quantitative type 2 | 0.01 | - | - | 0/3 | 0/4 | 0/4 | - | - | - | 5.19 |
| PC 38:4 | 24.251 | Quantitative type 2 | 0.01 | - | - | 0/3 | 0/4 | 0/4 | - | - | - | 5.22 |
| PC 38:5 | 19.16 | Quantitative type 2 | 0.01 | - | - | 0/3 | 0/4 | 0/4 | - | - | - | 5.63 |
| PC 38:6 | 15.889 | Quantitative type 2 | 0.01 | - | - | 0/3 | 0/4 | 0/4 | - | - | - | 6.46 |
| PC 40:1 | - | Quantitative type 2 | 0.1 | 0.3 | 1 | 0/3 | 0/4 | 0/4 | - | - | - | - |
| PC 40:2 | 0.266 | Quantitative type 2 | 0.01 | - | - | 0/3 | 0/4 | 0/4 | - | - | - | 10.96 |
| PC 40:3 | 0.356 | Quantitative type 2 | 0.01 | - | - | 0/3 | 0/4 | 3/4 | - | - | - | 10.85 |
| PC 40:4 | 0.885 | Quantitative type 2 | 0.01 | - | - | 0/3 | 0/4 | 0/4 | - | - | - | 3.63 |
| PC 40:5 | 2.219 | Quantitative type 2 | 0.01 | - | - | 0/3 | 0/4 | 0/4 | - | - | - | 4.93 |
| PC 40:6 | 3.811 | Quantitative type 2 | 0.01 | - | - | 0/3 | 0/4 | 0/4 | - | - | - | 5.28 |
| PC 42:0 | 0.14 | Quantitative type 2 | 0.05 | - | - | 0/3 | 0/4 | 0/4 | - | - | - | 7.36 |
| PC 42:1 | 0.113 | Quantitative type 2 | 0.01 | - | - | 0/3 | 0/4 | 0/4 | - | - | - | 4.97 |
| PC 42:2 | 0.142 | Quantitative type 2 | 0.01 | 0.1 | 1 | 0/3 | 0/4 | 0/4 | - | - | - | 4.96 |
| PC 42:4 | 0.117 | Quantitative type 2 | 0.01 | - | - | 0/3 | 0/4 | 0/4 | - | - | - | 7.10 |
| PC 42:5 | 0.156 | Quantitative type 2 | 0.01 | - | - | 0/3 | 0/4 | 0/4 | - | - | - | 3.00 |
| PC 42:6 | - | Quantitative type 2 | 0.01 | 0.2 | 1 | 0/3 | 0/4 | 0/4 | - | - | - | 17.79 |
| PC O-28:0 | 0.12 | Quantitative type 2 | 0.01 | 0.3 | 1 | 0/3 | 4/4 | 3/4 | - | - | - | - |
| PC O-28:1 | 0.232 | Quantitative type 2 | 0.01 | - | - | 0/3 | 4/4 | 3/4 | - | - | - | - |
| PC O-30:0 | 0.319 | Quantitative type 2 | 0.01 | 0.1 | 1 | 0/3 | 0/4 | 0/4 | - | - | - | 18.71 |
| PC O-30:1 | 1.148 | Not validated | 0.01 | 0.5 | 1 | 3/3 | 4/4 | 0/4 | - | - | - | - |
| PC O-30:2 | 0.101 | Quantitative type 2 | 0.01 | - | - | 0/3 | 0/4 | 3/4 | - | - | - | 28.05 |
| PC O-32:1 | 14.825 | Quantitative type 2 | 0.01 | - | - | 0/3 | 0/4 | 0/4 | - | - | - | 11.44 |
| PC O-32:2 | 2.297 | Quantitative type 2 | 0.01 | - | - | 0/3 | 0/4 | 0/4 | - | - | - | 9.07 |
| PC O-34:0 | 3.117 | Quantitative type 2 | 0.01 | - | - | 0/3 | 0/4 | 0/4 | - | - | - | 7.28 |
| PC O-34:1 | 9.126 | Quantitative type 2 | 0.01 | - | - | 0/3 | 0/4 | 0/4 | - | - | - | 9.21 |
| PC O-34:2 | 51.625 | Quantitative type 2 | 0.01 | - | - | 0/3 | 0/4 | 0/4 | - | - | - | 8.70 |
| PC O-34:3 | 73.009 | Quantitative type 2 | 0.01 | - | - | 0/3 | 0/4 | 0/4 | - | - | - | 9.85 |
| PC O-36:0 | 0.634 | Quantitative type 2 | 0.01 | - | - | 0/3 | 0/4 | 0/4 | - | - | - | 13.90 |
| PC O-36:1 | 4.993 | Quantitative type 2 | 0.01 | - | - | 0/3 | 0/4 | 0/4 | - | - | - | 7.98 |
| PC O-36:2 | 8.34 | Quantitative type 2 | 0.01 | - | - | 0/3 | 0/4 | 0/4 | - | - | - | 7.58 |
| PC O-36:3 | 20.393 | Quantitative type 2 | 0.01 | - | - | 0/3 | 0/4 | 0/4 | - | - | - | 7.63 |
| PC O-36:4 | 29.041 | Quantitative type 2 | 0.01 | - | - | 0/3 | 0/4 | 0/4 | - | - | - | 9.27 |
| PC O-36:5 | 187.403 | Quantitative type 2 | 0.01 | - | - | 0/3 | 0/4 | 0/4 | - | - | - | 9.25 |
| PC O-38:0 | 1.59 | Quantitative type 2 | 0.01 | - | - | 0/3 | 0/4 | 0/4 | - | - | - | 6.96 |
| PC O-38:1 | 0.293 | Quantitative type 2 | 0.01 | - | - | 0/3 | 0/4 | 3/4 | - | - | - | 14.74 |
| PC O-38:2 | 0.92 | Quantitative type 2 | 0.01 | - | - | 0/3 | 0/4 | 0/4 | - | - | - | 9.04 |
| PC O-38:3 | 1.685 | Quantitative type 2 | 0.01 | - | - | 0/3 | 0/4 | 0/4 | - | - | - | 7.99 |
| PC O-38:4 | 5.54 | Quantitative type 2 | 0.01 | - | - | 0/3 | 0/4 | 0/4 | - | - | - | 7.25 |
| PC O-38:5 | 42.518 | Quantitative type 2 | 0.01 | - | - | 0/3 | 0/4 | 0/4 | - | - | - | 7.00 |
| PC O-38:6 | 55.715 | Quantitative type 2 | 0.01 | - | - | 0/3 | 0/4 | 0/4 | - | - | - | 7.55 |
| PC O-40:1 | 2.378 | Quantitative type 2 | 0.01 | - | - | 0/3 | 0/4 | 0/4 | - | - | - | 16.49 |
| PC O-40:2 | 0.761 | Quantitative type 2 | 0.01 | - | - | 0/3 | 0/4 | 0/4 | - | - | - | 6.70 |
| PC O-40:3 | 0.429 | Quantitative type 2 | 0.01 | - | - | 0/3 | 0/4 | 0/4 | - | - | - | 8.36 |
| PC O-40:4 | 0.611 | Quantitative type 2 | 0.01 | - | - | 0/3 | 0/4 | 0/4 | - | - | - | 7.07 |
| PC O-40:5 | 1.71 | Quantitative type 2 | 0.01 | - | - | 0/3 | 0/4 | 0/4 | - | - | - | 6.99 |
| PC O-40:6 | 4.691 | Quantitative type 2 | 0.01 | - | - | 0/3 | 0/4 | 0/4 | - | - | - | 5.25 |
| PC O-42:0 | - | Not validated | 0.1 | 1 | 10 | 0/3 | 0/4 | 3/4 | - | - | - | - |
| PC O-42:1 | 0.186 | Quantitative type 2 | 0.01 | - | - | 0/3 | 0/4 | 0/4 | - | - | - | 27.46 |
| PC O-42:2 | 0.358 | Quantitative type 2 | 0.01 | - | - | 0/3 | 0/4 | 0/4 | - | - | - | 11.63 |
| PC O-42:3 | 0.657 | Quantitative type 2 | 0.01 | - | - | 0/3 | 0/4 | 0/4 | - | - | - | 9.03 |
| PC O-42:4 | 0.126 | Quantitative type 2 | 0.01 | - | - | 0/3 | 0/4 | 0/4 | - | - | - | 3.31 |
| PC O-42:5 | - | Quantitative type 2 | 0.01 | - | - | 0/3 | 0/4 | 0/4 | - | - | - | 4.43 |
| PC O-44:3 | 0.081 | Quantitative type 2 | 0.01 | - | - | 0/3 | 0/4 | 3/4 | - | - | - | 16.80 |
| PC O-44:4 | 0.126 | Quantitative type 2 | 0.01 | - | - | 0/3 | 0/4 | 0/4 | - | - | - | 9.29 |
| PC O-44:5 | 0.202 | Quantitative type 2 | 0.01 | - | - | 0/3 | 0/4 | 0/4 | - | - | - | 1.27 |
| PC O-44:6 | - | Quantitative type 2 | 0.01 | - | - | 0/3 | 0/4 | 0/4 | - | - | - | 6.65 |
| SM 33:1 | 0.868 | Quantitative type 2 | 0.01 | - | - | 0/3 | 0/4 | 0/4 | - | - | - | 6.18 |
| SM 34:1 | 29.041 | Quantitative type 2 | 0.01 | - | - | 0/3 | 0/4 | 0/4 | - | - | - | 4.85 |
| SM 34:2 | 1.517 | Quantitative type 2 | 0.01 | - | - | 0/3 | 0/4 | 0/4 | - | - | - | 5.21 |
| SM 35:1 | 1.149 | Quantitative type 2 | 0.01 | - | - | 0/3 | 0/4 | 3/4 | - | - | - | 6.54 |
| SM 36:1 | 18.507 | Quantitative type 2 | 0.01 | - | - | 0/3 | 0/4 | 3/4 | - | - | - | 4.83 |
| SM 36:2 | 3.01 | Quantitative type 2 | 0.01 | - | - | 0/3 | 0/4 | 3/4 | - | - | - | 6.13 |
| SM 38:3 | 0.04 | Quantitative type 2 | 0.01 | - | - | 0/3 | 0/4 | 3/4 | - | - | - | 13.74 |
| SM 41:1 | 3.555 | Quantitative type 2 | 0.01 | - | - | 0/3 | 0/4 | 3/4 | - | - | - | 1.26 |
| SM 41:2 | 1.543 | Quantitative type 2 | 0.01 | - | - | 0/3 | 0/4 | 3/4 | - | - | - | 3.60 |
| SM 42:1 | 8.056 | Quantitative type 2 | 0.01 | - | - | 0/3 | 0/4 | 3/4 | - | - | - | 1.24 |
| SM 42:2 | 15.671 | Quantitative type 2 | 0.01 | - | - | 0/3 | 0/4 | 3/4 | - | - | - | 2.73 |
| SM 43:1 | 0.289 | Quantitative type 2 | 0.01 | - | - | 0/3 | 0/4 | 0/4 | - | - | - | 2.39 |
| SM 44:1 | 0.093 | Quantitative type 2 | 0.01 | - | - | 0/3 | 0/4 | 0/4 | - | - | - | 7.56 |
| SM 44:2 | 0.178 | Quantitative type 2 | 0.01 | - | - | 0/3 | 0/4 | 0/4 | - | - | - | 5.04 |
| Hexose | - | Quantitative type 1 | 200 | 1000 | 15000 | 3/3 | 0/4 | 0/4 | 0/3 | 1/4 | 0/4 | - |
| TG 14:0_32:2 | 3.706 | Quantitative type 2 | 0.01 | - | - | 3/3 | 0/4 | 3/4 | - | - | - | 10.26 |
| TG 14:0_34:0 | 5.618 | Quantitative type 2 | 0.01 | - | - | 0/3 | 0/4 | 3/4 | - | - | - | 1.60 |
| TG 14:0_34:1 | 45.57 | Quantitative type 2 | 0.01 | - | - | 0/3 | 0/4 | 3/4 | - | - | - | 4.18 |
| TG 14:0_34:2 | 22.785 | Quantitative type 2 | 0.01 | - | - | 3/3 | 0/4 | 3/4 | - | - | - | 5.98 |
| TG 14:0_34:3 | 3.63 | Quantitative type 2 | 0.01 | - | - | 3/3 | 0/4 | 3/4 | - | - | - | 11.16 |
| TG 14:0_35:1 | 1.402 | Quantitative type 2 | 0.01 | - | - | 1/3 | 0/4 | 3/4 | - | - | - | 12.14 |
| TG 14:0_35:2 | 1.076 | Quantitative type 2 | 0.01 | 0.2 | 2 | 0/3 | 0/4 | 3/4 | - | - | - | 13.93 |
| TG 14:0_36:1 | 6.543 | Quantitative type 2 | 0.01 | - | - | 3/3 | 0/4 | 3/4 | - | - | - | 17.18 |
| TG 14:0_36:2 | 36.002 | Quantitative type 2 | 0.01 | - | - | 3/3 | 0/4 | 3/4 | - | - | - | 4.81 |
| TG 14:0_36:3 | 17.03 | Quantitative type 2 | 0.01 | - | - | 3/3 | 0/4 | 3/4 | - | - | - | 3.74 |
| TG 14:0_36:4 | 3.63 | Quantitative type 2 | 0.01 | - | - | 3/3 | 0/4 | 3/4 | - | - | - | 10.70 |
| TG 14:0_38:4 | 0.627 | Quantitative type 2 | 0.01 | - | - | 0/3 | 4/4 | 0/4 | - | - | - | - |
| TG 14:0_38:5 | 0.628 | Quantitative type 2 | 0.01 | - | - | 2/3 | 4/4 | 3/4 | - | - | - | - |
| TG 14:0_40:5 | - | Quantitative type 2 | 0.01 | 0.3 | 2 | 0/3 | 0/4 | 0/4 | - | - | - | - |
| TG 16:0_28:1 | 8.34 | Quantitative type 2 | 0.01 | - | - | 3/3 | 0/4 | 3/4 | - | - | - | 14.66 |
| TG 16:0_28:2 | 2.849 | Quantitative type 2 | 0.01 | - | - | 3/3 | 0/4 | 3/4 | - | - | - | 4.89 |
| TG 16:0_30:2 | 10.056 | Quantitative type 2 | 0.01 | - | - | 3/3 | 0/4 | 3/4 | - | - | - | 2.66 |
| TG 16:0_32:0 | 11.794 | Quantitative type 2 | 0.01 | - | - | 3/3 | 0/4 | 3/4 | - | - | - | 3.99 |
| TG 16:0_32:1 | 75.061 | Quantitative type 2 | 0.01 | - | - | 3/3 | 0/4 | 3/4 | - | - | - | 2.82 |
| TG 16:0_32:2 | 32.447 | Quantitative type 2 | 0.01 | - | - | 0/3 | 0/4 | 3/4 | - | - | - | 2.86 |
| TG 16:0_32:3 | 4.408 | Quantitative type 2 | 0.01 | - | - | 0/3 | 0/4 | 3/4 | - | - | - | 4.45 |
| TG 16:0_33:1 | 6.635 | Quantitative type 2 | 0.01 | - | - | 3/3 | 0/4 | 3/4 | - | - | - | 5.95 |
| TG 16:0_33:2 | 2.789 | Quantitative type 2 | 0.01 | - | - | 3/3 | 0/4 | 3/4 | - | - | - | 11.04 |
| TG 16:0_34:0 | 3.34 | Quantitative type 2 | 0.01 | - | - | 0/3 | 0/4 | 3/4 | - | - | - | 3.14 |
| TG 16:0_34:1 | 142.025 | Quantitative type 2 | 0.01 | - | - | 3/3 | 0/4 | 3/4 | - | - | - | 6.16 |
| TG 16:0_34:2 | 121.938 | Quantitative type 2 | 0.01 | - | - | 3/3 | 0/4 | 3/4 | - | - | - | 3.75 |
| TG 16:0_34:3 | 22.785 | Quantitative type 2 | 0.01 | - | - | 3/3 | 0/4 | 3/4 | - | - | - | 3.96 |
| TG 16:0_34:4 | 2.639 | Quantitative type 2 | 0.01 | - | - | 3/3 | 0/4 | 3/4 | - | - | - | 6.85 |
| TG 16:0_35:1 | 4.724 | Quantitative type 2 | 0.01 | - | - | 0/3 | 0/4 | 3/4 | - | - | - | 10.12 |
| TG 16:0_35:2 | 5.856 | Quantitative type 2 | 0.01 | - | - | 3/3 | 0/4 | 3/4 | - | - | - | 6.58 |
| TG 16:0_35:3 | 1.559 | Quantitative type 2 | 0.01 | - | - | 3/3 | 0/4 | 3/4 | - | - | - | 7.09 |
| TG 16:0_36:2 | 265.028 | Quantitative type 2 | 0.01 | - | - | 3/3 | 0/4 | 3/4 | - | - | - | 6.76 |
| TG 16:0_36:3 | 155.417 | Quantitative type 2 | 0.01 | - | - | 3/3 | 0/4 | 3/4 | - | - | - | 7.03 |
| TG 16:0_36:4 | 35.017 | Quantitative type 2 | 0.01 | - | - | 3/3 | 0/4 | 3/4 | - | - | - | 3.41 |
| TG 16:0_36:5 | 4.627 | Quantitative type 2 | 0.01 | - | - | 3/3 | 0/4 | 3/4 | - | - | - | 7.06 |
| TG 16:0_36:6 | 0.587 | Quantitative type 2 | 0.01 | - | - | 0/3 | 0/4 | 3/4 | - | - | - | 29.36 |
| TG 16:0_37:3 | 0.756 | Quantitative type 2 | 0.01 | - | - | 3/3 | 0/4 | 0/4 | - | - | - | 6.09 |
| TG 16:0_38:1 | - | Quantitative type 2 | 0.01 | - | - | 0/3 | 2/4 | 3/4 | - | - | - | - |
| TG 16:0_38:2 | 4.595 | Quantitative type 2 | 0.01 | - | - | 0/3 | 0/4 | 3/4 | - | - | - | 23.61 |
| TG 16:0_38:3 | 3.655 | Quantitative type 2 | 0.01 | - | - | 0/3 | 0/4 | 3/4 | - | - | - | 2.87 |
| TG 16:0_38:4 | 2.99 | Quantitative type 2 | 0.01 | - | - | 0/3 | 0/4 | 3/4 | - | - | - | 13.88 |
| TG 16:0_38:5 | 3.204 | Quantitative type 2 | 0.01 | - | - | 0/3 | 0/4 | 3/4 | - | - | - | 10.95 |
| TG 16:0_38:6 | 1.331 | Quantitative type 2 | 0.01 | - | - | 3/3 | 0/4 | 0/4 | - | - | - | 12.32 |
| TG 16:0_38:7 | 0.376 | Quantitative type 2 | 0.01 | - | - | 0/3 | 0/4 | 0/4 | - | - | - | 11.79 |
| TG 16:0_40:6 | 1.516 | Quantitative type 2 | 0.01 | - | - | 3/3 | 0/4 | 3/4 | - | - | - | 16.68 |
| TG 16:0_40:7 | 1.055 | Quantitative type 2 | 0.01 | - | - | 3/3 | 0/4 | 0/4 | - | - | - | 26.62 |
| TG 16:0_40:8 | - | Quantitative type 2 | 0.01 | - | - | 0/3 | 3/4 | 0/4 | - | - | - | - |
| TG 16:1_28:0 | 3.117 | Quantitative type 2 | 0.01 | - | - | 2/3 | 0/4 | 3/4 | - | - | - | 9.62 |
| TG 16:1_30:1 | 5.242 | Quantitative type 2 | 0.01 | - | - | 3/3 | 0/4 | 3/4 | - | - | - | 6.01 |
| TG 16:1_32:0 | 11.632 | Quantitative type 2 | 0.01 | - | - | 3/3 | 0/4 | 3/4 | - | - | - | 4.32 |
| TG 16:1_32:1 | 20.252 | Quantitative type 2 | 0.01 | - | - | 3/3 | 0/4 | 3/4 | - | - | - | 4.58 |
| TG 16:1_32:2 | 4.823 | Quantitative type 2 | 0.01 | - | - | 3/3 | 0/4 | 3/4 | - | - | - | 1.67 |
| TG 16:1_33:1 | 1.956 | Quantitative type 2 | 0.01 | - | - | 3/3 | 0/4 | 3/4 | - | - | - | 10.73 |
| TG 16:1_34:0 | 4.084 | Quantitative type 2 | 0.01 | - | - | 3/3 | 0/4 | 3/4 | - | - | - | 10.29 |
| TG 16:1_34:1 | 67.182 | Quantitative type 2 | 0.01 | - | - | 3/3 | 0/4 | 3/4 | - | - | - | 5.03 |
| TG 16:1_34:2 | 30.065 | Quantitative type 2 | 0.01 | - | - | 3/3 | 0/4 | 3/4 | - | - | - | 4.48 |
| TG 16:1_34:3 | 4.469 | Quantitative type 2 | 0.01 | - | - | 3/3 | 0/4 | 3/4 | - | - | - | 8.68 |
| TG 16:1_36:1 | 8.056 | Quantitative type 2 | 0.01 | - | - | 3/3 | 0/4 | 3/4 | - | - | - | 13.05 |
| TG 16:1_36:2 | 44.632 | Quantitative type 2 | 0.01 | - | - | 3/3 | 0/4 | 3/4 | - | - | - | 5.29 |
| TG 16:1_36:3 | 19.427 | Quantitative type 2 | 0.01 | - | - | 3/3 | 0/4 | 3/4 | - | - | - | 5.53 |
| TG 16:1_36:4 | 4.228 | Quantitative type 2 | 0.01 | - | - | 3/3 | 0/4 | 3/4 | - | - | - | 10.44 |
| TG 16:1_36:5 | 0.543 | Quantitative type 2 | 0.01 | - | - | 3/3 | 0/4 | 3/4 | - | - | - | 9.28 |
| TG 16:1_38:3 | 0.874 | Quantitative type 2 | 0.01 | - | - | 0/3 | 0/4 | 2/4 | - | - | - | 19.35 |
| TG 16:1_38:4 | 0.682 | Quantitative type 2 | 0.01 | - | - | 1/3 | 0/4 | 3/4 | - | - | - | 22.83 |
| TG 16:1_38:5 | 0.531 | Quantitative type 2 | 0.01 | - | - | 0/3 | 0/4 | 2/4 | - | - | - | 17.60 |
| TG 17:0_32:1 | 1.752 | Quantitative type 2 | 0.01 | 1 | 5 | 3/3 | 0/4 | 3/4 | - | - | - | - |
| TG 17:0_34:1 | 2.949 | Quantitative type 2 | 0.01 | - | - | 3/3 | 0/4 | 3/4 | - | - | - | 5.76 |
| TG 17:0_34:2 | 1.737 | Quantitative type 2 | 0.01 | - | - | 0/3 | 0/4 | 3/4 | - | - | - | 13.57 |
| TG 17:0_34:3 | 0.528 | Quantitative type 2 | 0.01 | - | - | 0/3 | 0/4 | 3/4 | - | - | - | 25.86 |
| TG 17:0_36:3 | 2.25 | Quantitative type 2 | 0.01 | - | - | 3/3 | 0/4 | 3/4 | - | - | - | 11.35 |
| TG 17:0_36:4 | 0.549 | Quantitative type 2 | 0.01 | - | - | 2/3 | 0/4 | 3/4 | - | - | - | 23.38 |
| TG 17:1_32:1 | 2.032 | Quantitative type 2 | 0.01 | 1 | 5 | 3/3 | 0/4 | 2/4 | - | - | - | - |
| TG 17:1_34:1 | 4.199 | Quantitative type 2 | 0.01 | - | - | 3/3 | 0/4 | 3/4 | - | - | - | 2.47 |
| TG 17:1_34:2 | 2.014 | Quantitative type 2 | 0.01 | - | - | 0/3 | 0/4 | 3/4 | - | - | - | 11.13 |
| TG 17:1_34:3 | 0.423 | Quantitative type 2 | 0.01 | - | - | 0/3 | 0/4 | 3/4 | - | - | - | 17.99 |
| TG 17:1_36:3 | 1.166 | Quantitative type 2 | 0.01 | - | - | 0/3 | 0/4 | 3/4 | - | - | - | 17.84 |
| TG 17:1_36:4 | 0.39 | Quantitative type 2 | 0.01 | - | - | 2/3 | 0/4 | 3/4 | - | - | - | 20.08 |
| TG 17:1_36:5 | - | Quantitative type 2 | 0.01 | - | - | 0/3 | 0/4 | 3/4 | - | - | - | - |
| TG 17:1_38:5 | - | Quantitative type 2 | 0.01 | 0.2 | 2 | 0/3 | 0/4 | 3/4 | - | - | - | - |
| TG 17:1_38:6 | - | Quantitative type 2 | 0.01 | 0.2 | 2 | 0/3 | 0/4 | 3/4 | - | - | - | - |
| TG 17:1_38:7 | - | Quantitative type 2 | 0.01 | - | - | 0/3 | 0/4 | 2/4 | - | - | - | - |
| TG 17:2_34:2 | - | Quantitative type 2 | 0.01 | - | - | 0/3 | 0/4 | 2/4 | - | - | - | - |
| TG 17:2_34:3 | - | Quantitative type 2 | 0.01 | 0.3 | 2 | 1/3 | 0/4 | 3/4 | - | - | - | 14.50 |
| TG 17:2_36:2 | 0.221 | Quantitative type 2 | 0.01 | 0.15 | 2 | 0/3 | 0/4 | 2/4 | - | - | - | - |
| TG 17:2_36:3 | 0.178 | Quantitative type 2 | 0.01 | 0.15 | 2 | 0/3 | 4/4 | 3/4 | - | - | - | - |
| TG 17:2_36:4 | 0.18 | Quantitative type 2 | 0.01 | 0.5 | 2 | 3/3 | 0/4 | 3/4 | - | - | - | 5.65 |
| TG 17:2_38:5 | 0.08 | Quantitative type 2 | 0.01 | 0.2 | 2 | 0/3 | 0/4 | 3/4 | - | - | - | - |
| TG 17:2_38:6 | - | Quantitative type 2 | 0.01 | 0.2 | 2 | 3/3 | 0/4 | 3/4 | - | - | - | - |
| TG 17:2_38:7 | - | Quantitative type 2 | 0.01 | 0.1 | 2 | 0/3 | 0/4 | 3/4 | - | - | - | 10.45 |
| TG 18:0_30:0 | 1.912 | Quantitative type 2 | 0.01 | 1 | 5 | 0/3 | 0/4 | 0/4 | - | - | - | 9.68 |
| TG 18:0_30:1 | 3.458 | Quantitative type 2 | 0.01 | - | - | 0/3 | 0/4 | 3/4 | - | - | - | 15.07 |
| TG 18:0_32:0 | 2.313 | Quantitative type 2 | 0.01 | 2 | 10 | 0/3 | 0/4 | 3/4 | - | - | - | 17.04 |
| TG 18:0_32:1 | 8.574 | Quantitative type 2 | 0.01 | - | - | 3/3 | 0/4 | 3/4 | - | - | - | 6.61 |
| TG 18:0_32:2 | 4.317 | Quantitative type 2 | 0.01 | - | - | 0/3 | 0/4 | 3/4 | - | - | - | 16.25 |
| TG 18:0_34:2 | 14.123 | Quantitative type 2 | 0.01 | - | - | 3/3 | 0/4 | 3/4 | - | - | - | 7.64 |
| TG 18:0_34:3 | 2.639 | Quantitative type 2 | 0.01 | - | - | 3/3 | 0/4 | 3/4 | - | - | - | 10.32 |
| TG 18:0_36:1 | 3.784 | Quantitative type 2 | 0.01 | 1 | 20 | 3/3 | 0/4 | 0/4 | - | - | - | 12.83 |
| TG 18:0_36:2 | 24.59 | Quantitative type 2 | 0.01 | - | - | 0/3 | 0/4 | 3/4 | - | - | - | 9.94 |
| TG 18:0_36:3 | 14.32 | Quantitative type 2 | 0.01 | - | - | 0/3 | 0/4 | 3/4 | - | - | - | 5.82 |
| TG 18:0_36:4 | 3.681 | Quantitative type 2 | 0.01 | - | - | 0/3 | 0/4 | 3/4 | - | - | - | 2.23 |
| TG 18:0_36:5 | 0.366 | Quantitative type 2 | 0.01 | - | - | 3/3 | 0/4 | 3/4 | - | - | - | 11.21 |
| TG 18:0_38:6 | - | Quantitative type 2 | 0.01 | - | - | 2/3 | 0/4 | 3/4 | - | - | - | 18.19 |
| TG 18:0_38:7 | - | Quantitative type 2 | 0.01 | 0.2 | 1 | 2/3 | 0/4 | 3/4 | - | - | - | 25.32 |
| TG 18:1_26:0 | 7.16 | Quantitative type 2 | 0.01 | - | - | 0/3 | 0/4 | 3/4 | - | - | - | 12.93 |
| TG 18:1_28:1 | 7.16 | Quantitative type 2 | 0.01 | - | - | 3/3 | 0/4 | 3/4 | - | - | - | 3.63 |
| TG 18:1_30:0 | 53.817 | Quantitative type 2 | 0.01 | - | - | 3/3 | 0/4 | 3/4 | - | - | - | 1.38 |
| TG 18:1_30:1 | 39.947 | Quantitative type 2 | 0.01 | - | - | 3/3 | 0/4 | 3/4 | - | - | - | 3.02 |
| TG 18:1_30:2 | 9.19 | Quantitative type 2 | 0.01 | - | - | 3/3 | 0/4 | 3/4 | - | - | - | 7.58 |
| TG 18:1_31:0 | 5.134 | Quantitative type 2 | 0.01 | - | - | 0/3 | 0/4 | 3/4 | - | - | - | 7.52 |
| TG 18:1_32:0 | 68.12 | Quantitative type 2 | 0.01 | - | - | 3/3 | 0/4 | 3/4 | - | - | - | 4.88 |
| TG 18:1_32:1 | 142.025 | Quantitative type 2 | 0.01 | - | - | 3/3 | 0/4 | 3/4 | - | - | - | 5.95 |
| TG 18:1_32:2 | 33.128 | Quantitative type 2 | 0.01 | - | - | 3/3 | 0/4 | 3/4 | - | - | - | 7.79 |
| TG 18:1_32:3 | 4.112 | Quantitative type 2 | 0.01 | - | - | 3/3 | 0/4 | 3/4 | - | - | - | 2.85 |
| TG 18:1_33:0 | 3.918 | Quantitative type 2 | 0.01 | - | - | 0/3 | 0/4 | 3/4 | - | - | - | 14.91 |
| TG 18:1_33:1 | 13.929 | Quantitative type 2 | 0.01 | - | - | 0/3 | 0/4 | 3/4 | - | - | - | 6.94 |
| TG 18:1_33:2 | 3.784 | Quantitative type 2 | 0.01 | - | - | 3/3 | 0/4 | 3/4 | - | - | - | 5.20 |
| TG 18:1_33:3 | 0.332 | Quantitative type 2 | 0.01 | - | - | 2/3 | 0/4 | 3/4 | - | - | - | 6.23 |
| TG 18:1_34:1 | 484.382 | Quantitative type 2 | 0.01 | - | - | 3/3 | 0/4 | 3/4 | - | - | - | 6.07 |
| TG 18:1_34:2 | 215.269 | Quantitative type 2 | 0.01 | - | - | 3/3 | 0/4 | 3/4 | - | - | - | 5.36 |
| TG 18:1_34:3 | 32.672 | Quantitative type 2 | 0.01 | - | - | 3/3 | 0/4 | 3/4 | - | - | - | 5.59 |
| TG 18:1_34:4 | 2.657 | Quantitative type 2 | 0.01 | - | - | 0/3 | 0/4 | 3/4 | - | - | - | 4.34 |
| TG 18:1_35:2 | 6.821 | Quantitative type 2 | 0.01 | - | - | 0/3 | 0/4 | 3/4 | - | - | - | 12.69 |
| TG 18:1_35:3 | 1.611 | Quantitative type 2 | 0.01 | - | - | 0/3 | 0/4 | 3/4 | - | - | - | 19.17 |
| TG 18:1_36:0 | 1.762 | Quantitative type 2 | 0.01 | 1 | 10 | 0/3 | 3/4 | 2/4 | - | - | - | - |
| TG 18:1_36:1 | 53.076 | Quantitative type 2 | 0.01 | - | - | 0/3 | 0/4 | 3/4 | - | - | - | 6.89 |
| TG 18:1_36:2 | 215.269 | Quantitative type 2 | 0.01 | - | - | 0/3 | 0/4 | 3/4 | - | - | - | 9.36 |
| TG 18:1_36:3 | 109.896 | Quantitative type 2 | 0.01 | - | - | 0/3 | 0/4 | 3/4 | - | - | - | 4.18 |
| TG 18:1_36:4 | 22.943 | Quantitative type 2 | 0.01 | - | - | 3/3 | 0/4 | 3/4 | - | - | - | 3.95 |
| TG 18:1_36:5 | 2.848 | Quantitative type 2 | 0.01 | - | - | 3/3 | 0/4 | 3/4 | - | - | - | 7.18 |
| TG 18:1_36:6 | 0.49 | Quantitative type 2 | 0.01 | - | - | 2/3 | 0/4 | 3/4 | - | - | - | 29.07 |
| TG 18:1_38:5 | 2.479 | Quantitative type 2 | 0.01 | - | - | 3/3 | 0/4 | 3/4 | - | - | - | 7.63 |
| TG 18:1_38:6 | 0.966 | Quantitative type 2 | 0.01 | - | - | 3/3 | 0/4 | 3/4 | - | - | - | 7.79 |
| TG 18:1_38:7 | 0.095 | Quantitative type 2 | 0.01 | 0.2 | 2 | 3/3 | 0/4 | 3/4 | - | - | - | 11.98 |
| TG 18:2_28:0 | 6.543 | Quantitative type 2 | 0.01 | - | - | 0/3 | 0/4 | 3/4 | - | - | - | 5.89 |
| TG 18:2_30:0 | 18.126 | Quantitative type 2 | 0.01 | - | - | 3/3 | 0/4 | 3/4 | - | - | - | 3.21 |
| TG 18:2_30:1 | 10.483 | Quantitative type 2 | 0.9 | 1.7 | 12 | 0/3 | 0/4 | 3/4 | - | - | - | 7.30 |
| TG 18:2_31:0 | 1.559 | Quantitative type 2 | 0.01 | - | - | 0/3 | 0/4 | 3/4 | - | - | - | 12.36 |
| TG 18:2_32:0 | 24.761 | Quantitative type 2 | 0.01 | - | - | 3/3 | 0/4 | 3/4 | - | - | - | 2.98 |
| TG 18:2_32:1 | 33.128 | Quantitative type 2 | 0.01 | - | - | 3/3 | 0/4 | 3/4 | - | - | - | 5.35 |
| TG 18:2_32:2 | 7.362 | Quantitative type 2 | 0.01 | - | - | 3/3 | 0/4 | 3/4 | - | - | - | 4.52 |
| TG 18:2_33:0 | 1.254 | Quantitative type 2 | 0.01 | - | - | 0/3 | 0/4 | 2/4 | - | - | - | 11.53 |
| TG 18:2_33:1 | 3.63 | Quantitative type 2 | 0.01 | - | - | 3/3 | 0/4 | 3/4 | - | - | - | 5.02 |
| TG 18:2_33:2 | 0.744 | Quantitative type 2 | 0.01 | - | - | 0/3 | 0/4 | 3/4 | - | - | - | 4.17 |
| TG 18:2_34:0 | 11.081 | Quantitative type 2 | 0.01 | - | - | 3/3 | 0/4 | 3/4 | - | - | - | 9.72 |
| TG 18:2_34:1 | 137.187 | Quantitative type 2 | 0.01 | - | - | 3/3 | 0/4 | 3/4 | - | - | - | 5.69 |
| TG 18:2_34:2 | 53.076 | Quantitative type 2 | 0.01 | - | - | 3/3 | 0/4 | 3/4 | - | - | - | 3.96 |
| TG 18:2_34:3 | 6.635 | Quantitative type 2 | 0.01 | - | - | 3/3 | 0/4 | 3/4 | - | - | - | 3.85 |
| TG 18:2_34:4 | 0.574 | Quantitative type 2 | 0.01 | - | - | 3/3 | 0/4 | 3/4 | - | - | - | 12.51 |
| TG 18:2_35:1 | 3.01 | Quantitative type 2 | 0.01 | - | - | 0/3 | 0/4 | 3/4 | - | - | - | 10.27 |
| TG 18:2_35:2 | 1.735 | Quantitative type 2 | 0.01 | - | - | 3/3 | 0/4 | 3/4 | - | - | - | 8.30 |
| TG 18:2_35:3 | 0.31 | Quantitative type 2 | 0.01 | - | - | 0/3 | 0/4 | 3/4 | - | - | - | 26.63 |
| TG 18:2_36:0 | 1.316 | Quantitative type 2 | 0.01 | - | - | 3/3 | 0/4 | 0/4 | - | - | - | 12.05 |
| TG 18:2_36:1 | 16 | Quantitative type 2 | 0.01 | - | - | 3/3 | 0/4 | 3/4 | - | - | - | 5.72 |
| TG 18:2_36:2 | 59.302 | Quantitative type 2 | 0.01 | - | - | 0/3 | 0/4 | 3/4 | - | - | - | 3.64 |
| TG 18:2_36:3 | 19.835 | Quantitative type 2 | 0.01 | - | - | 0/3 | 0/4 | 3/4 | - | - | - | 5.41 |
| TG 18:2_36:4 | 4.141 | Quantitative type 2 | 0.01 | - | - | 3/3 | 0/4 | 3/4 | - | - | - | 4.39 |
| TG 18:2_36:5 | 0.584 | Quantitative type 2 | 0.01 | - | - | 3/3 | 0/4 | 3/4 | - | - | - | 9.47 |
| TG 18:2_38:4 | 1.204 | Quantitative type 2 | 0.01 | - | - | 0/3 | 0/4 | 3/4 | - | - | - | 15.70 |
| TG 18:2_38:5 | 0.946 | Quantitative type 2 | 0.01 | - | - | 0/3 | 0/4 | 0/4 | - | - | - | 23.51 |
| TG 18:2_38:6 | - | Quantitative type 2 | 0.01 | - | - | 3/3 | 0/4 | 3/4 | - | - | - | 15.08 |
| TG 18:3_30:0 | 1.875 | Quantitative type 2 | 0.01 | - | - | 3/3 | 0/4 | 2/4 | - | - | - | 5.74 |
| TG 18:3_32:0 | 2.657 | Quantitative type 2 | 0.01 | - | - | 3/3 | 0/4 | 3/4 | - | - | - | 5.09 |
| TG 18:3_32:1 | 3.864 | Quantitative type 2 | 0.01 | - | - | 3/3 | 0/4 | 3/4 | - | - | - | 5.42 |
| TG 18:3_33:2 | - | Quantitative type 2 | 0.01 | 0.2 | 2 | 0/3 | 2/4 | 2/4 | - | - | - | - |
| TG 18:3_34:0 | 2.189 | Quantitative type 2 | 0.01 | - | - | 0/3 | 0/4 | 3/4 | - | - | - | 9.32 |
| TG 18:3_34:1 | 15.348 | Quantitative type 2 | 0.01 | - | - | 3/3 | 0/4 | 3/4 | - | - | - | 3.67 |
| TG 18:3_34:2 | 5.657 | Quantitative type 2 | 0.01 | - | - | 3/3 | 0/4 | 3/4 | - | - | - | 4.01 |
| TG 18:3_34:3 | 0.773 | Quantitative type 2 | 0.01 | - | - | 3/3 | 0/4 | 3/4 | - | - | - | 6.51 |
| TG 18:3_35:2 | - | Quantitative type 2 | 0.01 | - | - | 0/3 | 0/4 | 2/4 | - | - | - | - |
| TG 18:3_36:1 | 1.997 | Quantitative type 2 | 0.01 | - | - | 0/3 | 0/4 | 3/4 | - | - | - | 9.40 |
| TG 18:3_36:2 | 6.869 | Quantitative type 2 | 0.01 | - | - | 3/3 | 0/4 | 3/4 | - | - | - | 16.18 |
| TG 18:3_36:3 | 2.639 | Quantitative type 2 | 0.01 | - | - | 3/3 | 0/4 | 3/4 | - | - | - | 9.73 |
| TG 18:3_36:4 | 0.594 | Quantitative type 2 | 0.01 | - | - | 0/3 | 0/4 | 3/4 | - | - | - | 10.82 |
| TG 18:3_38:5 | - | Quantitative type 2 | 0.01 | - | - | 0/3 | 0/4 | 2/4 | - | - | - | - |
| TG 18:3_38:6 | - | Quantitative type 2 | 0.01 | 0.1 | 1 | 0/3 | 3/4 | 3/4 | - | - | - | - |
| TG 20:0_32:3 | 0.366 | Quantitative type 2 | 0.01 | - | - | 3/3 | 0/4 | 3/4 | - | - | - | 9.90 |
| TG 20:0_32:4 | 0.225 | Quantitative type 2 | 0.01 | - | - | 3/3 | 0/4 | 3/4 | - | - | - | 9.70 |
| TG 20:0_34:1 | - | Quantitative type 2 | 0.01 | - | - | 0/3 | 0/4 | 0/4 | - | - | - | - |
| TG 20:1_24:3 | - | Not validated | 1 | 3 | 10 | 0/3 | 0/4 | 0/4 | - | - | - | - |
| TG 20:1_26:1 | - | Not validated | 0.01 | 0.3 | 1 | 3/3 | 0/4 | 3/4 | - | - | - | - |
| TG 20:1_30:1 | 0.467 | Quantitative type 2 | 0.01 | 0.3 | 1 | 0/3 | 0/4 | 3/4 | - | - | - | - |
| TG 20:1_32:0 | 0.255 | Quantitative type 2 | 0.01 | - | - | 3/3 | 0/4 | 3/4 | - | - | - | 28.98 |
| TG 20:1_32:1 | 1.932 | Quantitative type 2 | 0.01 | - | - | 0/3 | 0/4 | 3/4 | - | - | - | 18.79 |
| TG 20:1_32:2 | 0.527 | Quantitative type 2 | 0.01 | 0.3 | 1 | 0/3 | 0/4 | 2/4 | - | - | - | - |
| TG 20:1_32:3 | - | Quantitative type 2 | 0.01 | - | - | 0/3 | 0/4 | 3/4 | - | - | - | - |
| TG 20:1_34:0 | - | Quantitative type 2 | 0.01 | - | - | 0/3 | 0/4 | 0/4 | - | - | - | - |
| TG 20:1_34:1 | 4.377 | Quantitative type 2 | 0.01 | - | - | 3/3 | 0/4 | 0/4 | - | - | - | 24.54 |
| TG 20:1_34:2 | 2.445 | Quantitative type 2 | 0.01 | - | - | 3/3 | 0/4 | 0/4 | - | - | - | 12.57 |
| TG 20:1_34:3 | 0.378 | Quantitative type 2 | 0.01 | - | - | 0/3 | 0/4 | 0/4 | - | - | - | - |
| TG 20:2_32:0 | - | Quantitative type 2 | 0.01 | - | - | 0/3 | 0/4 | 2/4 | - | - | - | 3.34 |
| TG 20:2_32:1 | 1.2 | Quantitative type 2 | 0.01 | - | - | 3/3 | 0/4 | 3/4 | - | - | - | 22.66 |
| TG 20:2_34:1 | 2.282 | Quantitative type 2 | 0.01 | - | - | 0/3 | 0/4 | 0/4 | - | - | - | 7.51 |
| TG 20:2_34:2 | 1.264 | Quantitative type 2 | 0.01 | 0.02 | 1 | 3/3 | 0/4 | 3/4 | - | - | - | - |
| TG 20:2_34:3 | - | Quantitative type 2 | 0.01 | 0.04 | 1 | 1/3 | 0/4 | 0/4 | - | - | - | - |
| TG 20:2_34:4 | - | Quantitative type 2 | 0.01 | - | - | 0/3 | 0/4 | 0/4 | - | - | - | - |
| TG 20:2_36:5 | - | Quantitative type 2 | 0.01 | - | - | 0/3 | 0/4 | 3/4 | - | - | - | - |
| TG 20:3_32:0 | 0.554 | Quantitative type 2 | 0.01 | - | - | 0/3 | 0/4 | 3/4 | - | - | - | 9.33 |
| TG 20:3_32:1 | 1.129 | Quantitative type 2 | 0.01 | - | - | 0/3 | 0/4 | 3/4 | - | - | - | 7.88 |
| TG 20:3_32:2 | 0.346 | Quantitative type 2 | 0.01 | - | - | 1/3 | 0/4 | 0/4 | - | - | - | 14.98 |
| TG 20:3_34:0 | - | Quantitative type 2 | 0.01 | - | - | 0/3 | 2/4 | 0/4 | - | - | - | - |
| TG 20:3_34:1 | 2.99 | Quantitative type 2 | 0.01 | - | - | 3/3 | 0/4 | 3/4 | - | - | - | 5.90 |
| TG 20:3_34:2 | 1.286 | Quantitative type 2 | 0.01 | - | - | 0/3 | 0/4 | 2/4 | - | - | - | 9.92 |
| TG 20:3_34:3 | - | Quantitative type 2 | 0.01 | - | - | 2/3 | 0/4 | 2/4 | - | - | - | 7.28 |
| TG 20:3_36:3 | 0.649 | Quantitative type 2 | 0.01 | - | - | 3/3 | 0/4 | 2/4 | - | - | - | 12.68 |
| TG 20:3_36:4 | - | Quantitative type 2 | 0.01 | - | - | 1/3 | 0/4 | 3/4 | - | - | - | 18.94 |
| TG 20:3_36:5 | - | Quantitative type 2 | 0.01 | 0.15 | 1 | 0/3 | 0/4 | 3/4 | - | - | - | 24.51 |
| TG 20:4_30:0 | 1.182 | Quantitative type 2 | 0.01 | - | - | 3/3 | 0/4 | 3/4 | - | - | - | 18.71 |
| TG 20:4_32:0 | 1.431 | Quantitative type 2 | 0.01 | - | - | 3/3 | 0/4 | 3/4 | - | - | - | 5.00 |
| TG 20:4_32:1 | 2.144 | Quantitative type 2 | 0.01 | - | - | 3/3 | 0/4 | 3/4 | - | - | - | 8.98 |
| TG 20:4_32:2 | 0.403 | Quantitative type 2 | 0.01 | - | - | 3/3 | 0/4 | 3/4 | - | - | - | 10.64 |
| TG 20:4_33:2 | - | Quantitative type 2 | 0.01 | 0.1 | 1 | 0/3 | 0/4 | 2/4 | - | - | - | - |
| TG 20:4_34:0 | 1.001 | Quantitative type 2 | 0.01 | - | - | 3/3 | 0/4 | 3/4 | - | - | - | 5.67 |
| TG 20:4_34:1 | 6.364 | Quantitative type 2 | 0.01 | - | - | 0/3 | 0/4 | 3/4 | - | - | - | 5.81 |
| TG 20:4_34:2 | 2.77 | Quantitative type 2 | 0.01 | - | - | 0/3 | 0/4 | 3/4 | - | - | - | 5.32 |
| TG 20:4_34:3 | 0.297 | Quantitative type 2 | 0.01 | - | - | 3/3 | 0/4 | 3/4 | - | - | - | 5.85 |
| TG 20:4_35:3 | - | Quantitative type 2 | 0.01 | 0.1 | 1 | 0/3 | 0/4 | 0/4 | - | - | - | - |
| TG 20:4_36:2 | 2.639 | Quantitative type 2 | 0.01 | - | - | 0/3 | 0/4 | 0/4 | - | - | - | 17.87 |
| TG 20:4_36:3 | 1.126 | Quantitative type 2 | 0.01 | - | - | 0/3 | 0/4 | 3/4 | - | - | - | 10.38 |
| TG 20:4_36:4 | 0.31 | Quantitative type 2 | 0.01 | - | - | 0/3 | 0/4 | 3/4 | - | - | - | 4.50 |
| TG 20:4_36:5 | - | Quantitative type 2 | 0.01 | - | - | 0/3 | 0/4 | 3/4 | - | - | - | - |
| TG 20:5_34:0 | - | Quantitative type 2 | 0.01 | - | - | 0/3 | 2/4 | 0/4 | - | - | - | - |
| TG 20:5_34:1 | 1.006 | Quantitative type 2 | 0.01 | - | - | 3/3 | 0/4 | 3/4 | - | - | - | 10.74 |
| TG 20:5_34:2 | 0.521 | Quantitative type 2 | 0.01 | - | - | 3/3 | 0/4 | 3/4 | - | - | - | 11.48 |
| TG 20:5_36:2 | 0.454 | Quantitative type 2 | 0.01 | - | - | 1/3 | 4/4 | 0/4 | - | - | - | - |
| TG 20:5_36:3 | - | Quantitative type 2 | 0.01 | - | - | 0/3 | 2/4 | 3/4 | - | - | - | - |
| TG 22:0_32:4 | - | Not validated | 0.01 | 0.2 | 1 | 0/3 | 2/4 | 3/4 | - | - | - | - |
| TG 22:1_32:5 | - | Not validated | 0.01 | 0.2 | 1 | 0/3 | 2/4 | 1/4 | - | - | - | - |
| TG 22:2_32:4 | - | Not validated | 0.05 | 0.2 | 1 | 0/3 | 0/4 | 3/4 | - | - | - | - |
| TG 22:3_30:2 | - | Not validated | 0.01 | 0.2 | 1 | 0/3 | 0/4 | 2/4 | - | - | - | - |
| TG 22:4_32:0 | 0.277 | Quantitative type 2 | 0.01 | 0.2 | 2 | 0/3 | 0/4 | 3/4 | - | - | - | 19.14 |
| TG 22:4_32:2 | - | Quantitative type 2 | 0.01 | 0.2 | 2 | 0/3 | 0/4 | 2/4 | - | - | - | - |
| TG 22:4_34:2 | 0.756 | Quantitative type 2 | 0.01 | - | - | 0/3 | 0/4 | 3/4 | - | - | - | 16.20 |
| TG 22:5_32:0 | 0.663 | Quantitative type 2 | 0.01 | - | - | 2/3 | 0/4 | 3/4 | - | - | - | 26.71 |
| TG 22:5_32:1 | 0.728 | Quantitative type 2 | 0.01 | - | - | 0/3 | 0/4 | 3/4 | - | - | - | 21.59 |
| TG 22:5_34:1 | 2.657 | Quantitative type 2 | 0.01 | - | - | 2/3 | 0/4 | 3/4 | - | - | - | 15.37 |
| TG 22:5_34:2 | 0.979 | Quantitative type 2 | 0.01 | - | - | 0/3 | 0/4 | 3/4 | - | - | - | 10.95 |
| TG 22:5_34:3 | - | Quantitative type 2 | 0.01 | - | - | 0/3 | 0/4 | 3/4 | - | - | - | - |
| TG 22:6_32:0 | 0.993 | Quantitative type 2 | 0.01 | - | - | 0/3 | 0/4 | 3/4 | - | - | - | 26.64 |
| TG 22:6_32:1 | 0.982 | Quantitative type 2 | 0.01 | - | - | 3/3 | 4/4 | 3/4 | - | - | - | - |
| TG 22:6_34:1 | 3.138 | Quantitative type 2 | 0.01 | - | - | 0/3 | 0/4 | 0/4 | - | - | - | 13.37 |
| TG 22:6_34:2 | 1.229 | Quantitative type 2 | 0.01 | - | - | 3/3 | 0/4 | 3/4 | - | - | - | 7.13 |
| TG 22:6_34:3 | - | Quantitative type 2 | 0.01 | - | - | 0/3 | 1/4 | 2/4 | - | - | - | - |
| Choline | 216.767 | Quantitative type 2 | 0.02 | 0.07 | 45 | 0/3 | 0/4 | 0/4 | - | - | - | 0.61 |

LOD: Limit of Detection, LLOQ: Lower-Limit of Quantitation, ULOQ: Upper-Limit of Quantitation, QC: Quality Control
