# Supplementary material for: Targeted metabolomics of postmortem human cardiac tissue using the Biocrates MxP Quant 500 kit
Source: PLoS One. 2026 Aug 19;21(8):e0356269. doi: 10.1371/journal.pone.0356269 (PMC13489478; doi:10.1371/journal.pone.0356269)
Supplement: Supplementary file 2 [file pone.0356269.s003.docx]

# S2 Table. Summary Table of Retained Metabolites Measured in Heart Tissue

| Metabolite | Class | Min conc (µM) | Median conc (µM) | Max conc (µM) | Proportion < LOD values, % | Proportion < LLOQ values, % | Proportion > ULOQ values, % |
| --- | --- | --- | --- | --- | --- | --- | --- |
| C0 | Acylcarnitines | 74.543 | 238.856 | 413.001 | 0.0 | 0.0 | 80.0 |
| C2 | Acylcarnitines | 3.074 | 6.636 | 48.168 | 2.5 | 0.0 | 10.0 |
| C3 | Acylcarnitines | 0.040 | 0.132 | 0.589 | 2.5 | 95.0 | 0.0 |
| C3-DC (C4-OH) | Acylcarnitines | 0.225 | 0.743 | 4.347 | 0.0 | 0.0 | 0.0 |
| C4 | Acylcarnitines | 0.074 | 0.297 | 1.142 | 5.0 | 0.0 | 0.0 |
| C5 | Acylcarnitines | 0.099 | 0.285 | 0.550 | 2.5 | 0.0 | 0.0 |
| C5-DC (C6-OH) | Acylcarnitines | 0.004 | 0.014 | 0.043 | 0.0 | 0.0 | 0.0 |
| C5-OH (C3-DC-M) | Acylcarnitines | 0.334 | 1.723 | 3.272 | 0.0 | 0.0 | 12.5 |
| C5:1 | Acylcarnitines | 0.099 | 0.363 | 0.832 | 2.5 | 0.0 | 0.0 |
| C14 | Acylcarnitines | 0.022 | 0.032 | 0.094 | 52.5 | 0.0 | 0.0 |
| C14:1-OH | Acylcarnitines | 0.030 | 0.049 | 0.102 | 7.5 | 0.0 | 0.0 |
| C16 | Acylcarnitines | 0.040 | 0.150 | 1.122 | 0.0 | 0.0 | 0.0 |
| C16-OH | Acylcarnitines | 0.002 | 0.003 | 0.015 | 2.5 | 0.0 | 0.0 |
| C16:1-OH | Acylcarnitines | 0.049 | 0.147 | 0.319 | 0.0 | 0.0 | 0.0 |
| C18 | Acylcarnitines | 0.067 | 0.552 | 3.249 | 0.0 | 0.0 | 0.0 |
| C18:1 | Acylcarnitines | 0.030 | 0.160 | 1.231 | 0.0 | 0.0 | 0.0 |
| C18:1-OH | Acylcarnitines | 0.004 | 0.016 | 0.039 | 0.0 | 0.0 | 0.0 |
| C18:2 | Acylcarnitines | 0.019 | 0.047 | 0.470 | 0.0 | 0.0 | 0.0 |
| Trigonelline | Alkaloids | 0.295 | 1.891 | 4.532 | 0.0 | 0.0 | 0.0 |
| Arg | Aminoacids | 28.443 | 352.139 | 1,351.176 | 0.0 | 0.0 | 20.0 |
| Asn | Aminoacids | 48.503 | 333.144 | 809.002 | 0.0 | 0.0 | 27.5 |
| Asp | Aminoacids | 37.271 | 188.706 | 455.087 | 0.0 | 0.0 | 0.0 |
| Cys | Aminoacids | 150.123 | 564.175 | 948.826 | 0.0 | 0.0 | 97.5 |
| Gln | Aminoacids | 657.114 | 1,910.852 | 3,326.986 | 0.0 | 0.0 | 65.0 |
| Glu | Aminoacids | 186.108 | 1,009.902 | 2,194.992 | 0.0 | 0.0 | 55.0 |
| Gly | Aminoacids | 282.088 | 897.644 | 1,782.888 | 0.0 | 0.0 | 0.0 |
| His | Aminoacids | 44.942 | 224.411 | 512.000 | 0.0 | 0.0 | 5.0 |
| Ile | Aminoacids | 86.823 | 744.434 | 2,194.992 | 0.0 | 0.0 | 72.5 |
| Lys | Aminoacids | 69.551 | 436.549 | 1,016.927 | 0.0 | 0.0 | 7.5 |
| Met | Aminoacids | 43.713 | 413.001 | 1,910.852 | 0.0 | 0.0 | 22.5 |
| Pro | Aminoacids | 134.364 | 652.575 | 2,702.352 | 0.0 | 0.0 | 30.0 |
| Thr | Aminoacids | 105.420 | 639.145 | 1,782.888 | 0.0 | 0.0 | 65.0 |
| Trp | Aminoacids | 19.293 | 164.279 | 519.147 | 0.0 | 0.0 | 7.5 |
| Tyr | Aminoacids | 58.081 | 477.713 | 1,351.176 | 0.0 | 0.0 | 50.0 |
| Val | Aminoacids | 117.784 | 891.444 | 3,104.188 | 0.0 | 0.0 | 52.5 |
| 1-Met-His | Aminoacids Related | 0.851 | 1.768 | 3.758 | 0.0 | 0.0 | 0.0 |
| 3-Met-His | Aminoacids Related | 0.182 | 1.037 | 10.411 | 0.0 | 0.0 | 0.0 |
| 5-AVA | Aminoacids Related | 0.119 | 0.978 | 75.584 | 0.0 | 0.0 | 2.5 |
| AABA | Aminoacids Related | 1.895 | 8.112 | 24.084 | 0.0 | 0.0 | 0.0 |
| ADMA | Aminoacids Related | 0.637 | 2.056 | 6.277 | 0.0 | 0.0 | 0.0 |
| alpha-AAA | Aminoacids Related | 0.293 | 1.638 | 3.972 | 0.0 | 17.5 | 0.0 |
| Anserine | Aminoacids Related | 0.067 | 0.403 | 3.655 | 0.0 | 65.0 | 0.0 |
| BABA | Aminoacids Related | 0.018 | 0.033 | 0.076 | 0.0 | 95.0 | 0.0 |
| Betaine | Aminoacids Related | 8.398 | 18.256 | 69.551 | 12.5 | 0.0 | 2.5 |
| Carnosine | Aminoacids Related | 1.345 | 3.811 | 17.268 | 0.0 | 0.0 | 0.0 |
| Cit | Aminoacids Related | 2.732 | 5.134 | 25.107 | 0.0 | 60.0 | 0.0 |
| Creatinine | Aminoacids Related | 25.107 | 81.572 | 146.018 | 0.0 | 0.0 | 0.0 |
| Cystine | Aminoacids Related | 7.210 | 32.447 | 101.125 | 0.0 | 0.0 | 0.0 |
| DOPA | Aminoacids Related | 0.029 | 0.075 | 0.339 | 10.0 | 87.5 | 0.0 |
| HArg | Aminoacids Related | 0.102 | 0.473 | 0.977 | 0.0 | 0.0 | 0.0 |
| HCys | Aminoacids Related | 9.580 | 39.671 | 136.239 | 0.0 | 0.0 | 0.0 |
| Kynurenine | Aminoacids Related | 0.379 | 0.663 | 1.196 | 0.0 | 90.0 | 0.0 |
| Met-SO | Aminoacids Related | 0.732 | 46.135 | 276.282 | 2.5 | 5.0 | 0.0 |
| Orn | Aminoacids Related | 12.126 | 53.817 | 398.932 | 0.0 | 0.0 | 0.0 |
| PheAlaBetaine | Aminoacids Related | 0.016 | 0.025 | 0.050 | 32.5 | 62.5 | 0.0 |
| ProBetaine | Aminoacids Related | 0.577 | 1.114 | 8.112 | 0.0 | 0.0 | 0.0 |
| Sarcosine | Aminoacids Related | 0.031 | 0.600 | 5.028 | 7.5 | 85.0 | 0.0 |
| SDMA | Aminoacids Related | 0.187 | 0.927 | 3.160 | 0.0 | 0.0 | 0.0 |
| t4-OH-Pro | Aminoacids Related | 3.434 | 10.056 | 25.813 | 0.0 | 0.0 | 0.0 |
| TrpBetaine | Aminoacids Related | 0.018 | 0.287 | 4.627 | 0.0 | 10.0 | 0.0 |
| CA | Bile Acids | 0.004 | 0.036 | 0.470 | 2.5 | 10.0 | 0.0 |
| CDCA | Bile Acids | 0.008 | 0.046 | 0.467 | 5.0 | 15.0 | 0.0 |
| DCA | Bile Acids | 0.043 | 0.072 | 0.170 | 30.0 | 0.0 | 0.0 |
| GCA | Bile Acids | 0.028 | 0.192 | 3.758 | 0.0 | 0.0 | 0.0 |
| GCDCA | Bile Acids | 0.018 | 0.135 | 1.876 | 0.0 | 0.0 | 0.0 |
| GDCA | Bile Acids | 0.001 | 0.099 | 1.968 | 0.0 | 2.5 | 0.0 |
| GLCA | Bile Acids | 0.003 | 0.005 | 0.081 | 27.5 | 0.0 | 0.0 |
| GLCAS | Bile Acids | 0.000 | 0.016 | 0.089 | 5.0 | 2.5 | 0.0 |
| GUDCA | Bile Acids | 0.001 | 0.010 | 0.218 | 5.0 | 7.5 | 0.0 |
| TCA | Bile Acids | 0.006 | 0.047 | 1.670 | 0.0 | 0.0 | 0.0 |
| TCDCA | Bile Acids | 0.010 | 0.040 | 1.251 | 0.0 | 0.0 | 0.0 |
| TDCA | Bile Acids | 0.003 | 0.020 | 0.636 | 2.5 | 2.5 | 0.0 |
| beta-Ala | Biogenic Amines | 2.928 | 10.126 | 32.000 | 0.0 | 0.0 | 0.0 |
| GABA | Biogenic Amines | 4.891 | 15.671 | 32.900 | 0.0 | 0.0 | 0.0 |
| Histamine | Biogenic Amines | 5.618 | 7.543 | 23.425 | 35.0 | 0.0 | 0.0 |
| Putrescine | Biogenic Amines | 0.321 | 1.022 | 6.635 | 0.0 | 0.0 | 0.0 |
| Spermidine | Biogenic Amines | 0.369 | 0.997 | 2.848 | 0.0 | 0.0 | 0.0 |
| Spermine | Biogenic Amines | 0.426 | 0.761 | 1.525 | 5.0 | 0.0 | 0.0 |
| HipAcid | Carboxylic Acids | 0.101 | 0.751 | 15.032 | 0.0 | 0.0 | 0.0 |
| Lac | Carboxylic Acids | 843.357 | 12,416.750 | 16,384.000 | 0.0 | 0.0 | 95.0 |
| OH-GlutAcid | Carboxylic Acids | 7.062 | 20.678 | 45.887 | 0.0 | 0.0 | 0.0 |
| Suc | Carboxylic Acids | 30.696 | 122.798 | 1,448.155 | 35.0 | 0.0 | 5.0 |
| Cer d16:1/18:0 | Ceramides | 0.277 | 0.773 | 2.313 | 0.0 | 0.0 | 57.5 |
| Cer d16:1/20:0 | Ceramides | 0.155 | 0.435 | 0.896 | 0.0 | 0.0 | 30.0 |
| Cer d16:1/22:0 | Ceramides | 0.170 | 0.569 | 1.246 | 0.0 | 0.0 | 0.0 |
| Cer d16:1/23:0 | Ceramides | 0.034 | 0.113 | 0.325 | 5.0 | 0.0 | 0.0 |
| Cer d16:1/24:0 | Ceramides | 0.031 | 0.074 | 0.165 | 0.0 | 0.0 | 0.0 |
| Cer d18:1/14:0 | Ceramides | 0.145 | 0.500 | 3.531 | 0.0 | 0.0 | 0.0 |
| Cer d18:1/16:0 | Ceramides | 0.595 | 1.974 | 9.849 | 0.0 | 0.0 | 0.0 |
| Cer d18:1/18:0 | Ceramides | 1.142 | 3.010 | 10.126 | 0.0 | 0.0 | 0.0 |
| Cer d18:1/18:1 | Ceramides | 0.053 | 0.343 | 3.784 | 2.5 | 0.0 | 0.0 |
| Cer d18:1/20:0 | Ceramides | 0.310 | 0.664 | 1.495 | 0.0 | 0.0 | 0.0 |
| Cer d18:1/20:0-OH | Ceramides | 0.325 | 1.108 | 9.318 | 47.5 | 0.0 | 0.0 |
| Cer d18:1/22:0 | Ceramides | 0.663 | 1.527 | 4.056 | 0.0 | 0.0 | 0.0 |
| Cer d18:1/23:0 | Ceramides | 0.264 | 0.662 | 1.434 | 0.0 | 0.0 | 37.5 |
| Cer d18:1/24:0 | Ceramides | 0.746 | 1.359 | 2.713 | 0.0 | 0.0 | 0.0 |
| Cer d18:1/24:1 | Ceramides | 1.356 | 4.287 | 12.126 | 0.0 | 0.0 | 0.0 |
| Cer d18:1/25:0 | Ceramides | 0.093 | 0.157 | 4.000 | 0.0 | 0.0 | 0.0 |
| Cer d18:1/26:0 | Ceramides | 0.032 | 0.073 | 1.370 | 12.5 | 0.0 | 0.0 |
| Cer d18:1/26:1 | Ceramides | 0.025 | 0.067 | 1.485 | 10.0 | 0.0 | 0.0 |
| Cer d18:2/14:0 | Ceramides | 0.010 | 0.043 | 0.218 | 10.0 | 0.0 | 2.5 |
| Cer d18:2/16:0 | Ceramides | 0.215 | 0.650 | 2.585 | 0.0 | 0.0 | 0.0 |
| Cer d18:2/18:0 | Ceramides | 0.470 | 1.473 | 4.000 | 0.0 | 0.0 | 0.0 |
| Cer d18:2/18:1 | Ceramides | 0.018 | 0.232 | 1.878 | 10.0 | 0.0 | 62.5 |
| Cer d18:2/20:0 | Ceramides | 0.275 | 0.890 | 2.204 | 0.0 | 0.0 | 0.0 |
| Cer d18:2/22:0 | Ceramides | 0.351 | 1.051 | 3.811 | 0.0 | 0.0 | 0.0 |
| Cer d18:2/23:0 | Ceramides | 0.053 | 0.177 | 0.604 | 0.0 | 0.0 | 0.0 |
| Cer d18:2/24:0 | Ceramides | 0.222 | 0.570 | 1.248 | 0.0 | 0.0 | 0.0 |
| Cer d18:2/24:1 | Ceramides | 0.366 | 0.861 | 2.770 | 0.0 | 0.0 | 0.0 |
| CE 14:0 | Cholesterol Esters | 1.011 | 4.141 | 6.727 | 40.0 | 0.0 | 0.0 |
| CE 15:0 | Cholesterol Esters | 1.597 | 3.053 | 5.205 | 0.0 | 0.0 | 0.0 |
| CE 15:1 | Cholesterol Esters | 0.283 | 0.723 | 1.344 | 0.0 | 0.0 | 5.0 |
| p-Cresol-SO4 | Cresols | 0.082 | 1.476 | 11.632 | 0.0 | 2.5 | 0.0 |
| DG 14:0_18:1 | Diacylglycerols | 0.314 | 1.177 | 5.816 | 7.5 | 0.0 | 0.0 |
| DG 14:0_18:2 | Diacylglycerols | 0.864 | 3.160 | 9.190 | 27.5 | 0.0 | 0.0 |
| DG 14:1_18:1 | Diacylglycerols | 0.056 | 0.193 | 1.549 | 55.0 | 0.0 | 0.0 |
| DG 16:0_16:0 | Diacylglycerols | 0.001 | 0.002 | 0.006 | 5.0 | 0.0 | 0.0 |
| DG 16:0_16:1 | Diacylglycerols | 0.204 | 1.818 | 16.450 | 15.0 | 0.0 | 0.0 |
| DG 16:0_18:1 | Diacylglycerols | 6.364 | 16.000 | 59.714 | 5.0 | 0.0 | 0.0 |
| DG 16:0_18:2 | Diacylglycerols | 2.969 | 6.774 | 22.316 | 0.0 | 0.0 | 0.0 |
| DG 16:1_18:0 | Diacylglycerols | 0.029 | 0.395 | 2.514 | 20.0 | 12.5 | 0.0 |
| DG 16:1_18:2 | Diacylglycerols | 0.257 | 0.952 | 9.318 | 20.0 | 0.0 | 0.0 |
| DG 17:0_18:1 | Diacylglycerols | 0.483 | 1.260 | 3.138 | 2.5 | 17.5 | 0.0 |
| DG 18:0_20:4 | Diacylglycerols | 0.448 | 2.928 | 23.425 | 22.5 | 0.0 | 0.0 |
| DG 18:1_18:1 | Diacylglycerols | 3.918 | 11.472 | 73.517 | 0.0 | 0.0 | 0.0 |
| DG 18:1_18:2 | Diacylglycerols | 4.438 | 13.269 | 57.680 | 0.0 | 0.0 | 0.0 |
| DG 18:1_18:3 | Diacylglycerols | 0.141 | 0.406 | 2.235 | 35.0 | 0.0 | 0.0 |
| DG 18:1_20:0 | Diacylglycerols | 0.096 | 0.358 | 1.230 | 75.0 | 0.0 | 0.0 |
| DG 18:1_20:1 | Diacylglycerols | 0.012 | 0.081 | 0.332 | 27.5 | 35.0 | 0.0 |
| DG 18:1_20:4 | Diacylglycerols | 0.099 | 0.272 | 0.780 | 57.5 | 0.0 | 0.0 |
| DG 18:2_18:2 | Diacylglycerols | 0.988 | 2.412 | 9.781 | 5.0 | 0.0 | 0.0 |
| Cer d18:0/18:0 | Dihydroceramides | 0.024 | 0.055 | 0.183 | 12.5 | 62.5 | 0.0 |
| FA 16:0 | Fatty Acids | 621.668 | 952.172 | 1,663.493 | 7.5 | 0.0 | 22.5 |
| FA 18:0 | Fatty Acids | 481.036 | 942.272 | 1,782.888 | 5.0 | 5.0 | 0.0 |
| FA 18:1 | Fatty Acids | 202.251 | 643.591 | 1,351.176 | 0.0 | 0.0 | 45.0 |
| FA 18:2 | Fatty Acids | 48.840 | 82.139 | 154.343 | 0.0 | 0.0 | 85.0 |
| FA 20:1 | Fatty Acids | 5.502 | 30.274 | 109.896 | 0.0 | 0.0 | 0.0 |
| FA 20:2 | Fatty Acids | 5.098 | 24.933 | 89.264 | 0.0 | 0.0 | 0.0 |
| FA 20:3 | Fatty Acids | 6.964 | 37.271 | 155.417 | 0.0 | 0.0 | 0.0 |
| FA 20:4n-6 (AA) | Fatty Acids | 153.277 | 292.036 | 382.681 | 0.0 | 0.0 | 97.5 |
| FA 20:5n-3 (EPA) | Fatty Acids | 8.340 | 29.857 | 129.787 | 0.0 | 0.0 | 47.5 |
| FA 22:6n-3 (DHA) | Fatty Acids | 26.909 | 265.028 | 680.287 | 0.0 | 0.0 | 95.0 |
| Hex2Cer d18:1/14:0 | Glycosylceramides | 0.044 | 0.100 | 0.264 | 55.0 | 0.0 | 0.0 |
| Hex2Cer d18:1/16:0 | Glycosylceramides | 0.387 | 0.787 | 5.979 | 0.0 | 0.0 | 0.0 |
| Hex2Cer d18:1/18:0 | Glycosylceramides | 0.281 | 0.644 | 4.595 | 0.0 | 0.0 | 0.0 |
| Hex2Cer d18:1/20:0 | Glycosylceramides | 0.126 | 0.264 | 0.796 | 2.5 | 0.0 | 0.0 |
| Hex2Cer d18:1/22:0 | Glycosylceramides | 0.337 | 0.672 | 1.672 | 0.0 | 0.0 | 0.0 |
| Hex2Cer d18:1/24:0 | Glycosylceramides | 0.222 | 0.497 | 1.796 | 2.5 | 0.0 | 0.0 |
| Hex2Cer d18:1/24:1 | Glycosylceramides | 0.255 | 0.588 | 2.828 | 0.0 | 0.0 | 0.0 |
| Hex3Cer d18:1/16:0 | Glycosylceramides | 0.929 | 1.761 | 3.434 | 0.0 | 0.0 | 0.0 |
| Hex3Cer d18:1/18:0 | Glycosylceramides | 0.463 | 1.080 | 2.313 | 0.0 | 0.0 | 50.0 |
| Hex3Cer d18:1/20:0 | Glycosylceramides | 0.074 | 0.177 | 0.398 | 0.0 | 0.0 | 0.0 |
| Hex3Cer d18:1/22:0 | Glycosylceramides | 1.030 | 2.173 | 4.347 | 0.0 | 0.0 | 0.0 |
| Hex3Cer d18:1/24:1 | Glycosylceramides | 1.506 | 4.691 | 8.000 | 0.0 | 0.0 | 0.0 |
| Hex-Cer d18:1/16:0 | Glycosylceramides | 0.035 | 0.091 | 0.398 | 20.0 | 77.5 | 0.0 |
| Hex-Cer d18:1/18:0 | Glycosylceramides | 0.020 | 0.090 | 6.105 | 27.5 | 20.0 | 0.0 |
| Hex-Cer d18:1/22:0 | Glycosylceramides | 0.202 | 0.361 | 3.182 | 2.5 | 0.0 | 0.0 |
| Hex-Cer d18:1/23:0 | Glycosylceramides | 0.031 | 0.156 | 4.891 | 40.0 | 0.0 | 0.0 |
| Hex-Cer d18:1/24:0 | Glycosylceramides | 0.091 | 0.207 | 5.776 | 0.0 | 0.0 | 0.0 |
| Hex-Cer d18:1/24:1 | Glycosylceramides | 0.328 | 0.926 | 102.537 | 0.0 | 0.0 | 0.0 |
| Cortisol | Hormones | 0.009 | 0.026 | 0.076 | 15.0 | 5.0 | 0.0 |
| DHEAS | Hormones | 0.019 | 0.109 | 0.222 | 0.0 | 0.0 | 0.0 |
| 3-IAA | Indoles Derivatives | 0.090 | 0.148 | 0.725 | 0.0 | 0.0 | 0.0 |
| 3-IPA | Indoles Derivatives | 0.007 | 0.017 | 0.219 | 15.0 | 10.0 | 0.0 |
| Ind-SO4 | Indoles Derivatives | 0.023 | 0.176 | 2.235 | 0.0 | 75.0 | 0.0 |
| Hypoxanthine | Nucleobases Related | 259.574 | 837.532 | 1,782.888 | 0.0 | 0.0 | 90.0 |
| Xanthine | Nucleobases Related | 29.651 | 72.004 | 199.466 | 0.0 | 0.0 | 0.0 |
| LPC 14:0 | Phosphatidylcholines | 2.639 | 6.423 | 18.507 | 7.5 | 90.0 | 0.0 |
| LPC 16:0 | Phosphatidylcholines | 13.833 | 65.799 | 162.017 | 0.0 | 0.0 | 0.0 |
| LPC 16:1 | Phosphatidylcholines | 1.092 | 2.770 | 7.890 | 0.0 | 0.0 | 0.0 |
| LPC 17:0 | Phosphatidylcholines | 0.593 | 1.320 | 3.630 | 0.0 | 0.0 | 0.0 |
| LPC 18:0 | Phosphatidylcholines | 6.453 | 16.564 | 29.243 | 0.0 | 0.0 | 0.0 |
| LPC 18:1 | Phosphatidylcholines | 5.028 | 22.943 | 125.366 | 0.0 | 0.0 | 0.0 |
| LPC 18:2 | Phosphatidylcholines | 3.434 | 22.162 | 126.238 | 0.0 | 0.0 | 0.0 |
| LPC 20:3 | Phosphatidylcholines | 0.444 | 1.759 | 6.190 | 0.0 | 0.0 | 0.0 |
| LPC 20:4 | Phosphatidylcholines | 7.727 | 30.696 | 78.793 | 0.0 | 0.0 | 0.0 |
| LPC 24:0 | Phosphatidylcholines | 0.039 | 0.071 | 0.212 | 0.0 | 0.0 | 0.0 |
| LPC 26:0 | Phosphatidylcholines | 0.034 | 0.059 | 0.237 | 0.0 | 0.0 | 0.0 |
| LPC 26:1 | Phosphatidylcholines | 0.078 | 0.180 | 0.602 | 0.0 | 0.0 | 47.5 |
| PC 24:0 | Phosphatidylcholines | 0.045 | 0.075 | 0.323 | 0.0 | 97.5 | 0.0 |
| PC 28:1 | Phosphatidylcholines | 0.518 | 0.896 | 1.571 | 0.0 | 0.0 | 0.0 |
| PC 30:0 | Phosphatidylcholines | 6.190 | 11.392 | 35.261 | 0.0 | 0.0 | 0.0 |
| PC 32:0 | Phosphatidylcholines | 57.282 | 82.711 | 186.108 | 0.0 | 0.0 | 0.0 |
| PC 32:1 | Phosphatidylcholines | 7.727 | 14.123 | 26.173 | 0.0 | 0.0 | 0.0 |
| PC 32:2 | Phosphatidylcholines | 0.721 | 2.694 | 7.062 | 0.0 | 0.0 | 0.0 |
| PC 32:3 | Phosphatidylcholines | 0.110 | 0.420 | 0.759 | 0.0 | 0.0 | 0.0 |
| PC 34:1 | Phosphatidylcholines | 87.427 | 196.720 | 359.538 | 0.0 | 0.0 | 0.0 |
| PC 34:2 | Phosphatidylcholines | 23.918 | 112.986 | 302.334 | 0.0 | 0.0 | 0.0 |
| PC 34:3 | Phosphatidylcholines | 2.173 | 7.835 | 16.564 | 0.0 | 0.0 | 0.0 |
| PC 34:4 | Phosphatidylcholines | 0.680 | 1.696 | 4.691 | 0.0 | 0.0 | 0.0 |
| PC 36:0 | Phosphatidylcholines | 4.823 | 17.753 | 42.518 | 0.0 | 0.0 | 0.0 |
| PC 36:1 | Phosphatidylcholines | 17.509 | 30.696 | 49.522 | 0.0 | 0.0 | 0.0 |
| PC 36:2 | Phosphatidylcholines | 16.564 | 50.914 | 96.336 | 0.0 | 0.0 | 0.0 |
| PC 36:3 | Phosphatidylcholines | 8.225 | 28.641 | 56.493 | 0.0 | 0.0 | 0.0 |
| PC 36:4 | Phosphatidylcholines | 38.586 | 84.449 | 173.645 | 0.0 | 0.0 | 0.0 |
| PC 36:5 | Phosphatidylcholines | 5.502 | 12.042 | 29.651 | 0.0 | 0.0 | 0.0 |
| PC 36:6 | Phosphatidylcholines | 0.330 | 0.941 | 2.694 | 0.0 | 0.0 | 0.0 |
| PC 38:0 | Phosphatidylcholines | 3.138 | 8.877 | 13.833 | 0.0 | 0.0 | 0.0 |
| PC 38:3 | Phosphatidylcholines | 3.630 | 6.681 | 8.938 | 0.0 | 0.0 | 0.0 |
| PC 38:4 | Phosphatidylcholines | 12.641 | 24.251 | 46.527 | 0.0 | 0.0 | 0.0 |
| PC 38:5 | Phosphatidylcholines | 10.853 | 19.160 | 33.591 | 0.0 | 0.0 | 0.0 |
| PC 38:6 | Phosphatidylcholines | 5.816 | 15.889 | 30.065 | 0.0 | 0.0 | 0.0 |
| PC 40:2 | Phosphatidylcholines | 0.113 | 0.266 | 0.508 | 0.0 | 0.0 | 0.0 |
| PC 40:3 | Phosphatidylcholines | 0.189 | 0.356 | 0.619 | 0.0 | 0.0 | 0.0 |
| PC 40:4 | Phosphatidylcholines | 0.438 | 0.885 | 1.565 | 0.0 | 0.0 | 0.0 |
| PC 40:5 | Phosphatidylcholines | 1.110 | 2.219 | 3.811 | 0.0 | 0.0 | 0.0 |
| PC 40:6 | Phosphatidylcholines | 1.601 | 3.811 | 7.062 | 0.0 | 0.0 | 0.0 |
| PC 42:0 | Phosphatidylcholines | 0.066 | 0.140 | 0.207 | 0.0 | 0.0 | 0.0 |
| PC 42:1 | Phosphatidylcholines | 0.061 | 0.113 | 0.224 | 0.0 | 0.0 | 0.0 |
| PC 42:2 | Phosphatidylcholines | 0.064 | 0.142 | 0.321 | 0.0 | 17.5 | 0.0 |
| PC 42:4 | Phosphatidylcholines | 0.053 | 0.117 | 0.204 | 0.0 | 0.0 | 0.0 |
| PC 42:5 | Phosphatidylcholines | 0.079 | 0.156 | 0.274 | 0.0 | 0.0 | 0.0 |
| PC O-28:0 | Phosphatidylcholines | 0.067 | 0.120 | 0.325 | 2.5 | 40.0 | 0.0 |
| PC O-28:1 | Phosphatidylcholines | 0.097 | 0.232 | 0.473 | 0.0 | 0.0 | 0.0 |
| PC O-30:0 | Phosphatidylcholines | 0.224 | 0.319 | 0.905 | 20.0 | 0.0 | 2.5 |
| PC O-30:1 | Phosphatidylcholines | 0.379 | 1.148 | 3.387 | 0.0 | 0.0 | 97.5 |
| PC O-30:2 | Phosphatidylcholines | 0.033 | 0.101 | 0.232 | 0.0 | 0.0 | 0.0 |
| PC O-32:1 | Phosphatidylcholines | 6.681 | 14.825 | 26.723 | 0.0 | 0.0 | 0.0 |
| PC O-32:2 | Phosphatidylcholines | 1.027 | 2.297 | 4.925 | 0.0 | 0.0 | 0.0 |
| PC O-34:0 | Phosphatidylcholines | 1.676 | 3.117 | 7.464 | 0.0 | 0.0 | 0.0 |
| PC O-34:1 | Phosphatidylcholines | 5.352 | 9.126 | 14.123 | 0.0 | 0.0 | 0.0 |
| PC O-34:2 | Phosphatidylcholines | 18.765 | 51.625 | 80.449 | 0.0 | 0.0 | 0.0 |
| PC O-34:3 | Phosphatidylcholines | 16.912 | 73.009 | 182.278 | 0.0 | 0.0 | 0.0 |
| PC O-36:0 | Phosphatidylcholines | 0.274 | 0.634 | 1.421 | 0.0 | 0.0 | 0.0 |
| PC O-36:1 | Phosphatidylcholines | 2.085 | 4.993 | 11.314 | 0.0 | 0.0 | 0.0 |
| PC O-36:2 | Phosphatidylcholines | 3.706 | 8.340 | 16.111 | 0.0 | 0.0 | 0.0 |
| PC O-36:3 | Phosphatidylcholines | 7.413 | 20.393 | 45.887 | 0.0 | 0.0 | 0.0 |
| PC O-36:4 | Phosphatidylcholines | 10.703 | 29.041 | 60.129 | 0.0 | 0.0 | 0.0 |
| PC O-36:5 | Phosphatidylcholines | 60.129 | 187.403 | 388.023 | 0.0 | 0.0 | 0.0 |
| PC O-38:0 | Phosphatidylcholines | 0.543 | 1.590 | 2.928 | 0.0 | 0.0 | 0.0 |
| PC O-38:1 | Phosphatidylcholines | 0.058 | 0.293 | 0.583 | 12.5 | 0.0 | 0.0 |
| PC O-38:2 | Phosphatidylcholines | 0.346 | 0.920 | 1.257 | 0.0 | 0.0 | 0.0 |
| PC O-38:3 | Phosphatidylcholines | 0.702 | 1.685 | 2.603 | 0.0 | 0.0 | 0.0 |
| PC O-38:4 | Phosphatidylcholines | 2.713 | 5.540 | 9.126 | 0.0 | 0.0 | 0.0 |
| PC O-38:5 | Phosphatidylcholines | 17.630 | 42.518 | 71.012 | 0.0 | 0.0 | 0.0 |
| PC O-38:6 | Phosphatidylcholines | 23.588 | 55.715 | 106.891 | 0.0 | 0.0 | 0.0 |
| PC O-40:1 | Phosphatidylcholines | 0.719 | 2.378 | 4.469 | 0.0 | 0.0 | 0.0 |
| PC O-40:2 | Phosphatidylcholines | 0.418 | 0.761 | 1.757 | 0.0 | 0.0 | 0.0 |
| PC O-40:3 | Phosphatidylcholines | 0.210 | 0.429 | 0.723 | 0.0 | 0.0 | 0.0 |
| PC O-40:4 | Phosphatidylcholines | 0.358 | 0.611 | 1.044 | 0.0 | 0.0 | 0.0 |
| PC O-40:5 | Phosphatidylcholines | 0.904 | 1.710 | 2.908 | 0.0 | 0.0 | 0.0 |
| PC O-40:6 | Phosphatidylcholines | 2.014 | 4.691 | 7.160 | 0.0 | 0.0 | 0.0 |
| PC O-42:1 | Phosphatidylcholines | 0.117 | 0.186 | 0.412 | 0.0 | 0.0 | 0.0 |
| PC O-42:2 | Phosphatidylcholines | 0.132 | 0.358 | 0.619 | 0.0 | 0.0 | 0.0 |
| PC O-42:3 | Phosphatidylcholines | 0.180 | 0.657 | 1.224 | 0.0 | 0.0 | 0.0 |
| PC O-42:4 | Phosphatidylcholines | 0.055 | 0.126 | 0.297 | 0.0 | 0.0 | 0.0 |
| PC O-44:3 | Phosphatidylcholines | 0.040 | 0.081 | 0.142 | 0.0 | 0.0 | 0.0 |
| PC O-44:4 | Phosphatidylcholines | 0.063 | 0.126 | 0.184 | 5.0 | 0.0 | 0.0 |
| PC O-44:5 | Phosphatidylcholines | 0.116 | 0.202 | 0.511 | 0.0 | 0.0 | 0.0 |
| SM 33:1 | Sphingomyelins | 0.497 | 0.868 | 2.297 | 0.0 | 0.0 | 0.0 |
| SM 34:1 | Sphingomyelins | 19.698 | 29.041 | 48.168 | 0.0 | 0.0 | 0.0 |
| SM 34:2 | Sphingomyelins | 0.784 | 1.517 | 3.031 | 0.0 | 0.0 | 0.0 |
| SM 35:1 | Sphingomyelins | 0.623 | 1.149 | 2.888 | 0.0 | 0.0 | 0.0 |
| SM 36:1 | Sphingomyelins | 8.754 | 18.507 | 44.632 | 0.0 | 0.0 | 0.0 |
| SM 36:2 | Sphingomyelins | 1.819 | 3.010 | 5.278 | 0.0 | 0.0 | 0.0 |
| SM 38:3 | Sphingomyelins | 0.011 | 0.040 | 0.064 | 0.0 | 0.0 | 0.0 |
| SM 41:1 | Sphingomyelins | 1.617 | 3.555 | 8.225 | 0.0 | 0.0 | 0.0 |
| SM 41:2 | Sphingomyelins | 0.747 | 1.543 | 3.095 | 0.0 | 0.0 | 0.0 |
| SM 42:1 | Sphingomyelins | 4.959 | 8.056 | 12.126 | 0.0 | 0.0 | 0.0 |
| SM 42:2 | Sphingomyelins | 10.126 | 15.671 | 29.041 | 0.0 | 0.0 | 0.0 |
| SM 43:1 | Sphingomyelins | 0.177 | 0.289 | 0.572 | 0.0 | 0.0 | 0.0 |
| SM 44:1 | Sphingomyelins | 0.060 | 0.093 | 0.187 | 0.0 | 0.0 | 0.0 |
| SM 44:2 | Sphingomyelins | 0.103 | 0.178 | 0.949 | 0.0 | 0.0 | 0.0 |
| TG 14:0_32:2 | Triacylglycerols | 0.063 | 3.706 | 13.361 | 2.5 | 0.0 | 0.0 |
| TG 14:0_34:0 | Triacylglycerols | 0.486 | 5.618 | 14.221 | 0.0 | 0.0 | 0.0 |
| TG 14:0_34:1 | Triacylglycerols | 2.828 | 45.570 | 118.603 | 0.0 | 0.0 | 0.0 |
| TG 14:0_34:2 | Triacylglycerols | 1.027 | 22.785 | 64.893 | 0.0 | 0.0 | 0.0 |
| TG 14:0_34:3 | Triacylglycerols | 0.058 | 3.630 | 9.646 | 5.0 | 0.0 | 0.0 |
| TG 14:0_35:1 | Triacylglycerols | 0.127 | 1.402 | 2.868 | 10.0 | 0.0 | 0.0 |
| TG 14:0_35:2 | Triacylglycerols | 0.115 | 1.076 | 2.428 | 7.5 | 5.0 | 0.0 |
| TG 14:0_36:1 | Triacylglycerols | 1.193 | 6.543 | 22.162 | 0.0 | 0.0 | 0.0 |
| TG 14:0_36:2 | Triacylglycerols | 2.676 | 36.002 | 81.572 | 0.0 | 0.0 | 0.0 |
| TG 14:0_36:3 | Triacylglycerols | 0.785 | 17.030 | 34.776 | 0.0 | 0.0 | 0.0 |
| TG 14:0_36:4 | Triacylglycerols | 0.158 | 3.630 | 7.210 | 2.5 | 0.0 | 0.0 |
| TG 14:0_38:4 | Triacylglycerols | 0.033 | 0.627 | 1.233 | 25.0 | 0.0 | 0.0 |
| TG 14:0_38:5 | Triacylglycerols | 0.154 | 0.628 | 1.308 | 27.5 | 0.0 | 0.0 |
| TG 16:0_28:1 | Triacylglycerols | 0.164 | 8.340 | 26.538 | 2.5 | 0.0 | 0.0 |
| TG 16:0_28:2 | Triacylglycerols | 0.262 | 2.849 | 10.556 | 2.5 | 0.0 | 0.0 |
| TG 16:0_30:2 | Triacylglycerols | 0.176 | 10.056 | 23.425 | 0.0 | 0.0 | 0.0 |
| TG 16:0_32:0 | Triacylglycerols | 3.031 | 11.794 | 62.683 | 0.0 | 0.0 | 0.0 |
| TG 16:0_32:1 | Triacylglycerols | 5.134 | 75.061 | 215.269 | 0.0 | 0.0 | 0.0 |
| TG 16:0_32:2 | Triacylglycerols | 1.257 | 32.447 | 98.360 | 0.0 | 0.0 | 0.0 |
| TG 16:0_32:3 | Triacylglycerols | 0.187 | 4.408 | 11.472 | 2.5 | 0.0 | 0.0 |
| TG 16:0_33:1 | Triacylglycerols | 0.562 | 6.635 | 16.679 | 0.0 | 0.0 | 0.0 |
| TG 16:0_33:2 | Triacylglycerols | 0.183 | 2.789 | 6.589 | 0.0 | 0.0 | 0.0 |
| TG 16:0_34:0 | Triacylglycerols | 0.262 | 3.340 | 32.447 | 25.0 | 0.0 | 0.0 |
| TG 16:0_34:1 | Triacylglycerols | 20.821 | 142.025 | 481.036 | 0.0 | 0.0 | 0.0 |
| TG 16:0_34:2 | Triacylglycerols | 9.448 | 121.938 | 393.440 | 0.0 | 0.0 | 0.0 |
| TG 16:0_34:3 | Triacylglycerols | 1.241 | 22.785 | 66.718 | 0.0 | 0.0 | 0.0 |
| TG 16:0_34:4 | Triacylglycerols | 0.104 | 2.639 | 6.320 | 0.0 | 0.0 | 0.0 |
| TG 16:0_35:1 | Triacylglycerols | 0.722 | 4.724 | 12.906 | 0.0 | 0.0 | 0.0 |
| TG 16:0_35:2 | Triacylglycerols | 0.682 | 5.856 | 12.906 | 0.0 | 0.0 | 0.0 |
| TG 16:0_35:3 | Triacylglycerols | 0.101 | 1.559 | 4.028 | 2.5 | 0.0 | 0.0 |
| TG 16:0_36:2 | Triacylglycerols | 36.002 | 265.028 | 634.730 | 0.0 | 0.0 | 0.0 |
| TG 16:0_36:3 | Triacylglycerols | 13.548 | 155.417 | 333.144 | 0.0 | 0.0 | 0.0 |
| TG 16:0_36:4 | Triacylglycerols | 2.266 | 35.017 | 78.793 | 0.0 | 0.0 | 0.0 |
| TG 16:0_36:5 | Triacylglycerols | 0.221 | 4.627 | 10.778 | 2.5 | 0.0 | 0.0 |
| TG 16:0_36:6 | Triacylglycerols | 0.038 | 0.587 | 1.275 | 22.5 | 0.0 | 0.0 |
| TG 16:0_37:3 | Triacylglycerols | 0.078 | 0.756 | 1.544 | 10.0 | 0.0 | 0.0 |
| TG 16:0_38:2 | Triacylglycerols | 1.096 | 4.595 | 14.026 | 0.0 | 0.0 | 0.0 |
| TG 16:0_38:3 | Triacylglycerols | 0.670 | 3.655 | 10.411 | 0.0 | 0.0 | 0.0 |
| TG 16:0_38:4 | Triacylglycerols | 0.483 | 2.990 | 8.282 | 0.0 | 0.0 | 0.0 |
| TG 16:0_38:5 | Triacylglycerols | 0.515 | 3.204 | 7.890 | 0.0 | 0.0 | 0.0 |
| TG 16:0_38:6 | Triacylglycerols | 0.215 | 1.331 | 2.809 | 7.5 | 0.0 | 0.0 |
| TG 16:0_38:7 | Triacylglycerols | 0.041 | 0.376 | 1.350 | 47.5 | 0.0 | 0.0 |
| TG 16:0_40:6 | Triacylglycerols | 0.451 | 1.516 | 5.352 | 2.5 | 0.0 | 0.0 |
| TG 16:0_40:7 | Triacylglycerols | 0.191 | 1.055 | 2.282 | 10.0 | 0.0 | 0.0 |
| TG 16:1_28:0 | Triacylglycerols | 0.063 | 3.117 | 10.196 | 5.0 | 0.0 | 0.0 |
| TG 16:1_30:1 | Triacylglycerols | 0.163 | 5.242 | 21.706 | 0.0 | 0.0 | 0.0 |
| TG 16:1_32:0 | Triacylglycerols | 0.821 | 11.632 | 35.506 | 0.0 | 0.0 | 0.0 |
| TG 16:1_32:1 | Triacylglycerols | 0.903 | 20.252 | 101.125 | 0.0 | 0.0 | 0.0 |
| TG 16:1_32:2 | Triacylglycerols | 0.172 | 4.823 | 22.943 | 0.0 | 0.0 | 0.0 |
| TG 16:1_33:1 | Triacylglycerols | 0.149 | 1.956 | 6.190 | 2.5 | 0.0 | 0.0 |
| TG 16:1_34:0 | Triacylglycerols | 0.600 | 4.084 | 13.833 | 2.5 | 0.0 | 0.0 |
| TG 16:1_34:1 | Triacylglycerols | 4.469 | 67.182 | 296.112 | 0.0 | 0.0 | 0.0 |
| TG 16:1_34:2 | Triacylglycerols | 1.368 | 30.065 | 138.141 | 0.0 | 0.0 | 0.0 |
| TG 16:1_34:3 | Triacylglycerols | 0.151 | 4.469 | 16.912 | 2.5 | 0.0 | 0.0 |
| TG 16:1_36:1 | Triacylglycerols | 1.218 | 8.056 | 21.259 | 0.0 | 0.0 | 0.0 |
| TG 16:1_36:2 | Triacylglycerols | 3.482 | 44.632 | 165.421 | 0.0 | 0.0 | 0.0 |
| TG 16:1_36:3 | Triacylglycerols | 1.157 | 19.427 | 45.255 | 0.0 | 0.0 | 0.0 |
| TG 16:1_36:4 | Triacylglycerols | 0.171 | 4.228 | 8.112 | 2.5 | 0.0 | 0.0 |
| TG 16:1_36:5 | Triacylglycerols | 0.024 | 0.543 | 1.179 | 25.0 | 0.0 | 0.0 |
| TG 16:1_38:3 | Triacylglycerols | 0.027 | 0.874 | 2.266 | 17.5 | 0.0 | 0.0 |
| TG 16:1_38:4 | Triacylglycerols | 0.089 | 0.682 | 2.751 | 15.0 | 0.0 | 0.0 |
| TG 16:1_38:5 | Triacylglycerols | 0.158 | 0.531 | 1.600 | 25.0 | 0.0 | 0.0 |
| TG 17:0_32:1 | Triacylglycerols | 0.094 | 1.752 | 3.580 | 5.0 | 25.0 | 0.0 |
| TG 17:0_34:1 | Triacylglycerols | 0.563 | 2.949 | 8.282 | 0.0 | 0.0 | 0.0 |
| TG 17:0_34:2 | Triacylglycerols | 0.117 | 1.737 | 3.411 | 2.5 | 0.0 | 0.0 |
| TG 17:0_34:3 | Triacylglycerols | 0.021 | 0.528 | 1.402 | 30.0 | 0.0 | 0.0 |
| TG 17:0_36:3 | Triacylglycerols | 0.203 | 2.250 | 4.627 | 0.0 | 0.0 | 0.0 |
| TG 17:0_36:4 | Triacylglycerols | 0.026 | 0.549 | 1.498 | 25.0 | 0.0 | 0.0 |
| TG 17:1_32:1 | Triacylglycerols | 0.401 | 2.032 | 5.028 | 2.5 | 40.0 | 0.0 |
| TG 17:1_34:1 | Triacylglycerols | 0.412 | 4.199 | 10.196 | 0.0 | 0.0 | 0.0 |
| TG 17:1_34:2 | Triacylglycerols | 0.098 | 2.014 | 5.242 | 2.5 | 0.0 | 0.0 |
| TG 17:1_34:3 | Triacylglycerols | 0.025 | 0.423 | 1.087 | 32.5 | 0.0 | 0.0 |
| TG 17:1_36:3 | Triacylglycerols | 0.149 | 1.166 | 2.329 | 7.5 | 0.0 | 0.0 |
| TG 17:1_36:4 | Triacylglycerols | 0.049 | 0.390 | 0.719 | 40.0 | 0.0 | 0.0 |
| TG 17:2_36:2 | Triacylglycerols | 0.045 | 0.221 | 0.601 | 57.5 | 22.5 | 0.0 |
| TG 17:2_36:3 | Triacylglycerols | 0.044 | 0.178 | 0.486 | 52.5 | 32.5 | 0.0 |
| TG 17:2_36:4 | Triacylglycerols | 0.024 | 0.180 | 0.762 | 15.0 | 80.0 | 0.0 |
| TG 17:2_38:5 | Triacylglycerols | 0.018 | 0.080 | 0.301 | 22.5 | 72.5 | 0.0 |
| TG 18:0_30:0 | Triacylglycerols | 0.310 | 1.912 | 7.516 | 0.0 | 45.0 | 0.0 |
| TG 18:0_30:1 | Triacylglycerols | 0.392 | 3.458 | 9.714 | 0.0 | 0.0 | 0.0 |
| TG 18:0_32:0 | Triacylglycerols | 0.966 | 2.313 | 12.729 | 0.0 | 75.0 | 0.0 |
| TG 18:0_32:1 | Triacylglycerols | 1.183 | 8.574 | 26.355 | 0.0 | 0.0 | 0.0 |
| TG 18:0_32:2 | Triacylglycerols | 0.270 | 4.317 | 9.514 | 0.0 | 0.0 | 0.0 |
| TG 18:0_34:2 | Triacylglycerols | 2.071 | 14.123 | 43.111 | 0.0 | 0.0 | 0.0 |
| TG 18:0_34:3 | Triacylglycerols | 0.264 | 2.639 | 7.160 | 2.5 | 0.0 | 0.0 |
| TG 18:0_36:1 | Triacylglycerols | 1.228 | 3.784 | 19.160 | 0.0 | 20.0 | 0.0 |
| TG 18:0_36:2 | Triacylglycerols | 8.282 | 24.590 | 85.036 | 0.0 | 0.0 | 0.0 |
| TG 18:0_36:3 | Triacylglycerols | 3.387 | 14.320 | 41.355 | 0.0 | 0.0 | 0.0 |
| TG 18:0_36:4 | Triacylglycerols | 0.713 | 3.681 | 12.641 | 0.0 | 0.0 | 0.0 |
| TG 18:0_36:5 | Triacylglycerols | 0.050 | 0.366 | 1.155 | 25.0 | 0.0 | 0.0 |
| TG 18:1_26:0 | Triacylglycerols | 0.997 | 7.160 | 19.698 | 0.0 | 0.0 | 0.0 |
| TG 18:1_28:1 | Triacylglycerols | 0.875 | 7.160 | 21.259 | 0.0 | 0.0 | 0.0 |
| TG 18:1_30:0 | Triacylglycerols | 3.249 | 53.817 | 134.364 | 0.0 | 0.0 | 0.0 |
| TG 18:1_30:1 | Triacylglycerols | 1.968 | 39.947 | 109.896 | 0.0 | 0.0 | 0.0 |
| TG 18:1_30:2 | Triacylglycerols | 0.199 | 9.190 | 20.112 | 0.0 | 0.0 | 0.0 |
| TG 18:1_31:0 | Triacylglycerols | 0.646 | 5.134 | 12.553 | 0.0 | 0.0 | 0.0 |
| TG 18:1_32:0 | Triacylglycerols | 9.781 | 68.120 | 233.941 | 0.0 | 0.0 | 0.0 |
| TG 18:1_32:1 | Triacylglycerols | 9.580 | 142.025 | 477.713 | 0.0 | 0.0 | 0.0 |
| TG 18:1_32:2 | Triacylglycerols | 1.536 | 33.128 | 92.411 | 0.0 | 0.0 | 0.0 |
| TG 18:1_32:3 | Triacylglycerols | 0.096 | 4.112 | 8.938 | 2.5 | 0.0 | 0.0 |
| TG 18:1_33:0 | Triacylglycerols | 0.616 | 3.918 | 12.553 | 0.0 | 0.0 | 0.0 |
| TG 18:1_33:1 | Triacylglycerols | 1.170 | 13.929 | 26.538 | 0.0 | 0.0 | 0.0 |
| TG 18:1_33:2 | Triacylglycerols | 0.171 | 3.784 | 6.498 | 0.0 | 0.0 | 0.0 |
| TG 18:1_33:3 | Triacylglycerols | 0.017 | 0.332 | 0.680 | 27.5 | 0.0 | 0.0 |
| TG 18:1_34:1 | Triacylglycerols | 64.000 | 484.382 | 1,097.496 | 0.0 | 0.0 | 0.0 |
| TG 18:1_34:2 | Triacylglycerols | 20.535 | 215.269 | 530.056 | 0.0 | 0.0 | 0.0 |
| TG 18:1_34:3 | Triacylglycerols | 1.721 | 32.672 | 64.893 | 0.0 | 0.0 | 0.0 |
| TG 18:1_34:4 | Triacylglycerols | 0.151 | 2.657 | 6.105 | 2.5 | 0.0 | 0.0 |
| TG 18:1_35:2 | Triacylglycerols | 0.776 | 6.821 | 12.996 | 0.0 | 0.0 | 0.0 |
| TG 18:1_35:3 | Triacylglycerols | 0.074 | 1.611 | 3.010 | 5.0 | 0.0 | 0.0 |
| TG 18:1_36:0 | Triacylglycerols | 0.122 | 1.762 | 10.928 | 50.0 | 42.5 | 0.0 |
| TG 18:1_36:1 | Triacylglycerols | 18.252 | 53.076 | 191.341 | 0.0 | 0.0 | 0.0 |
| TG 18:1_36:2 | Triacylglycerols | 39.397 | 215.269 | 533.742 | 0.0 | 0.0 | 0.0 |
| TG 18:1_36:3 | Triacylglycerols | 13.548 | 109.896 | 252.476 | 0.0 | 0.0 | 0.0 |
| TG 18:1_36:4 | Triacylglycerols | 2.282 | 22.943 | 58.081 | 0.0 | 0.0 | 0.0 |
| TG 18:1_36:5 | Triacylglycerols | 0.150 | 2.848 | 6.320 | 2.5 | 0.0 | 0.0 |
| TG 18:1_36:6 | Triacylglycerols | 0.040 | 0.490 | 0.920 | 25.0 | 0.0 | 0.0 |
| TG 18:1_38:5 | Triacylglycerols | 0.634 | 2.479 | 5.816 | 0.0 | 0.0 | 0.0 |
| TG 18:1_38:6 | Triacylglycerols | 0.277 | 0.966 | 1.985 | 10.0 | 0.0 | 0.0 |
| TG 18:1_38:7 | Triacylglycerols | 0.025 | 0.095 | 0.312 | 50.0 | 45.0 | 0.0 |
| TG 18:2_28:0 | Triacylglycerols | 0.245 | 6.543 | 16.223 | 0.0 | 0.0 | 0.0 |
| TG 18:2_30:0 | Triacylglycerols | 0.783 | 18.126 | 42.814 | 0.0 | 0.0 | 0.0 |
| TG 18:2_30:1 | Triacylglycerols | 0.401 | 10.483 | 23.588 | 0.0 | 7.5 | 10.0 |
| TG 18:2_31:0 | Triacylglycerols | 0.772 | 1.559 | 2.428 | 20.0 | 0.0 | 0.0 |
| TG 18:2_32:0 | Triacylglycerols | 2.412 | 24.761 | 72.505 | 0.0 | 0.0 | 0.0 |
| TG 18:2_32:1 | Triacylglycerols | 1.567 | 33.128 | 85.627 | 0.0 | 0.0 | 0.0 |
| TG 18:2_32:2 | Triacylglycerols | 0.270 | 7.362 | 16.450 | 0.0 | 0.0 | 0.0 |
| TG 18:2_33:0 | Triacylglycerols | 0.092 | 1.254 | 3.411 | 5.0 | 0.0 | 0.0 |
| TG 18:2_33:1 | Triacylglycerols | 0.196 | 3.630 | 8.225 | 2.5 | 0.0 | 0.0 |
| TG 18:2_33:2 | Triacylglycerols | 0.051 | 0.744 | 1.944 | 7.5 | 0.0 | 0.0 |
| TG 18:2_34:0 | Triacylglycerols | 2.713 | 11.081 | 49.522 | 0.0 | 0.0 | 0.0 |
| TG 18:2_34:1 | Triacylglycerols | 13.086 | 137.187 | 296.112 | 0.0 | 0.0 | 0.0 |
| TG 18:2_34:2 | Triacylglycerols | 2.789 | 53.076 | 114.563 | 0.0 | 0.0 | 0.0 |
| TG 18:2_34:3 | Triacylglycerols | 0.295 | 6.635 | 12.641 | 2.5 | 0.0 | 0.0 |
| TG 18:2_34:4 | Triacylglycerols | 0.024 | 0.574 | 1.001 | 20.0 | 0.0 | 0.0 |
| TG 18:2_35:1 | Triacylglycerols | 0.339 | 3.010 | 5.736 | 0.0 | 0.0 | 0.0 |
| TG 18:2_35:2 | Triacylglycerols | 0.103 | 1.735 | 3.706 | 5.0 | 0.0 | 0.0 |
| TG 18:2_35:3 | Triacylglycerols | 0.033 | 0.310 | 0.851 | 27.5 | 0.0 | 0.0 |
| TG 18:2_36:0 | Triacylglycerols | 0.240 | 1.316 | 6.589 | 20.0 | 0.0 | 0.0 |
| TG 18:2_36:1 | Triacylglycerols | 3.758 | 16.000 | 46.527 | 0.0 | 0.0 | 0.0 |
| TG 18:2_36:2 | Triacylglycerols | 7.674 | 59.302 | 139.102 | 0.0 | 0.0 | 0.0 |
| TG 18:2_36:3 | Triacylglycerols | 1.928 | 19.835 | 75.584 | 0.0 | 0.0 | 0.0 |
| TG 18:2_36:4 | Triacylglycerols | 0.270 | 4.141 | 17.509 | 0.0 | 0.0 | 0.0 |
| TG 18:2_36:5 | Triacylglycerols | 0.027 | 0.584 | 1.594 | 20.0 | 0.0 | 0.0 |
| TG 18:2_38:4 | Triacylglycerols | 0.332 | 1.204 | 3.010 | 7.5 | 0.0 | 0.0 |
| TG 18:2_38:5 | Triacylglycerols | 0.132 | 0.946 | 2.189 | 20.0 | 0.0 | 0.0 |
| TG 18:3_30:0 | Triacylglycerols | 0.047 | 1.875 | 4.347 | 5.0 | 0.0 | 0.0 |
| TG 18:3_32:0 | Triacylglycerols | 0.203 | 2.657 | 9.448 | 0.0 | 0.0 | 0.0 |
| TG 18:3_32:1 | Triacylglycerols | 0.118 | 3.864 | 8.515 | 2.5 | 0.0 | 0.0 |
| TG 18:3_34:0 | Triacylglycerols | 0.126 | 2.189 | 6.453 | 2.5 | 0.0 | 0.0 |
| TG 18:3_34:1 | Triacylglycerols | 1.080 | 15.348 | 38.854 | 0.0 | 0.0 | 0.0 |
| TG 18:3_34:2 | Triacylglycerols | 0.133 | 5.657 | 11.236 | 2.5 | 0.0 | 0.0 |
| TG 18:3_34:3 | Triacylglycerols | 0.037 | 0.773 | 1.599 | 10.0 | 0.0 | 0.0 |
| TG 18:3_36:1 | Triacylglycerols | 0.224 | 1.997 | 6.498 | 0.0 | 0.0 | 0.0 |
| TG 18:3_36:2 | Triacylglycerols | 0.483 | 6.869 | 13.548 | 0.0 | 0.0 | 0.0 |
| TG 18:3_36:3 | Triacylglycerols | 0.200 | 2.639 | 6.277 | 5.0 | 0.0 | 0.0 |
| TG 18:3_36:4 | Triacylglycerols | 0.029 | 0.594 | 1.674 | 12.5 | 0.0 | 0.0 |
| TG 20:0_32:3 | Triacylglycerols | 0.030 | 0.366 | 0.862 | 10.0 | 0.0 | 0.0 |
| TG 20:0_32:4 | Triacylglycerols | 0.029 | 0.225 | 0.677 | 27.5 | 0.0 | 0.0 |
| TG 20:1_30:1 | Triacylglycerols | 0.041 | 0.467 | 1.291 | 5.0 | 35.0 | 5.0 |
| TG 20:1_32:0 | Triacylglycerols | 0.064 | 0.255 | 1.013 | 12.5 | 0.0 | 0.0 |
| TG 20:1_32:1 | Triacylglycerols | 0.116 | 1.932 | 6.190 | 2.5 | 0.0 | 0.0 |
| TG 20:1_32:2 | Triacylglycerols | 0.049 | 0.527 | 1.683 | 10.0 | 42.5 | 0.0 |
| TG 20:1_34:1 | Triacylglycerols | 0.958 | 4.377 | 14.929 | 0.0 | 0.0 | 0.0 |
| TG 20:1_34:2 | Triacylglycerols | 0.432 | 2.445 | 6.681 | 0.0 | 0.0 | 0.0 |
| TG 20:1_34:3 | Triacylglycerols | 0.026 | 0.378 | 0.807 | 17.5 | 0.0 | 0.0 |
| TG 20:2_32:1 | Triacylglycerols | 0.039 | 1.200 | 3.605 | 5.0 | 0.0 | 0.0 |
| TG 20:2_34:1 | Triacylglycerols | 0.463 | 2.282 | 5.816 | 0.0 | 0.0 | 0.0 |
| TG 20:2_34:2 | Triacylglycerols | 0.061 | 1.264 | 2.621 | 5.0 | 0.0 | 12.5 |
| TG 20:3_32:0 | Triacylglycerols | 0.182 | 0.554 | 1.537 | 15.0 | 0.0 | 0.0 |
| TG 20:3_32:1 | Triacylglycerols | 0.044 | 1.129 | 3.434 | 5.0 | 0.0 | 0.0 |
| TG 20:3_32:2 | Triacylglycerols | 0.026 | 0.346 | 0.875 | 40.0 | 0.0 | 0.0 |
| TG 20:3_34:1 | Triacylglycerols | 0.270 | 2.990 | 7.362 | 5.0 | 0.0 | 0.0 |
| TG 20:3_34:2 | Triacylglycerols | 0.139 | 1.286 | 2.969 | 5.0 | 0.0 | 0.0 |
| TG 20:3_36:3 | Triacylglycerols | 0.028 | 0.649 | 1.574 | 15.0 | 0.0 | 0.0 |
| TG 20:4_30:0 | Triacylglycerols | 0.191 | 1.182 | 2.479 | 5.0 | 0.0 | 0.0 |
| TG 20:4_32:0 | Triacylglycerols | 0.275 | 1.431 | 3.227 | 0.0 | 0.0 | 0.0 |
| TG 20:4_32:1 | Triacylglycerols | 0.198 | 2.144 | 6.409 | 2.5 | 0.0 | 0.0 |
| TG 20:4_32:2 | Triacylglycerols | 0.062 | 0.403 | 1.025 | 15.0 | 0.0 | 0.0 |
| TG 20:4_34:0 | Triacylglycerols | 0.432 | 1.001 | 2.621 | 2.5 | 0.0 | 0.0 |
| TG 20:4_34:1 | Triacylglycerols | 1.427 | 6.364 | 15.562 | 0.0 | 0.0 | 0.0 |
| TG 20:4_34:2 | Triacylglycerols | 0.531 | 2.770 | 5.579 | 0.0 | 0.0 | 0.0 |
| TG 20:4_34:3 | Triacylglycerols | 0.033 | 0.297 | 0.689 | 20.0 | 0.0 | 0.0 |
| TG 20:4_36:2 | Triacylglycerols | 1.321 | 2.639 | 6.869 | 0.0 | 0.0 | 0.0 |
| TG 20:4_36:3 | Triacylglycerols | 0.480 | 1.126 | 2.770 | 2.5 | 0.0 | 0.0 |
| TG 20:4_36:4 | Triacylglycerols | 0.049 | 0.310 | 1.073 | 45.0 | 0.0 | 0.0 |
| TG 20:5_34:1 | Triacylglycerols | 0.153 | 1.006 | 3.095 | 5.0 | 0.0 | 0.0 |
| TG 20:5_34:2 | Triacylglycerols | 0.045 | 0.521 | 1.557 | 25.0 | 0.0 | 0.0 |
| TG 20:5_36:2 | Triacylglycerols | 0.048 | 0.454 | 1.134 | 42.5 | 0.0 | 0.0 |
| TG 22:4_32:0 | Triacylglycerols | 0.041 | 0.277 | 1.070 | 30.0 | 32.5 | 0.0 |
| TG 22:4_34:2 | Triacylglycerols | 0.020 | 0.756 | 1.502 | 17.5 | 0.0 | 0.0 |
| TG 22:5_32:0 | Triacylglycerols | 0.196 | 0.663 | 2.014 | 10.0 | 0.0 | 0.0 |
| TG 22:5_32:1 | Triacylglycerols | 0.102 | 0.728 | 1.982 | 5.0 | 0.0 | 0.0 |
| TG 22:5_34:1 | Triacylglycerols | 0.476 | 2.657 | 9.063 | 0.0 | 0.0 | 0.0 |
| TG 22:5_34:2 | Triacylglycerols | 0.209 | 0.979 | 3.010 | 5.0 | 0.0 | 0.0 |
| TG 22:6_32:0 | Triacylglycerols | 0.227 | 0.993 | 2.462 | 5.0 | 0.0 | 0.0 |
| TG 22:6_32:1 | Triacylglycerols | 0.127 | 0.982 | 2.099 | 5.0 | 0.0 | 0.0 |
| TG 22:6_34:1 | Triacylglycerols | 0.435 | 3.138 | 7.890 | 2.5 | 0.0 | 0.0 |
| TG 22:6_34:2 | Triacylglycerols | 0.176 | 1.229 | 3.706 | 10.0 | 0.0 | 0.0 |
| Choline | Vitamins & Cofactors | 80.449 | 216.767 | 556.408 | 0.0 | 0.0 | 97.5 |
