# Supplementary material for: Targeted metabolomics of postmortem human cardiac tissue using the Biocrates MxP Quant 500 kit
Source: PLoS One. 2026 Aug 19;21(8):e0356269. doi: 10.1371/journal.pone.0356269 (PMC13489478; doi:10.1371/journal.pone.0356269)
Supplement: Supplementary file 3 [file pone.0356269.s004.docx]

| S3 Table. Spearman Correlation between PMI and metabolite class scores | | | |
| --- | --- | --- | --- |
| **Class** | **Spearman’s ρ** | **Unadjusted p-value** | **FDR adjusted p-value** |
| Biogenic Amines | 0.427 | 0.006 | 0.055 |
| Fatty Acids | 0.430 | 0.006 | 0.055 |
| Glycosylceramides | 0.415 | 0.008 | 0.055 |
| Aminoacids Related | 0.389 | 0.013 | 0.069 |
| Aminoacids | 0.273 | 0.088 | 0.265 |
| Cresols | -0.280 | 0.080 | 0.265 |
| Vitamins & Cofactors | 0.275 | 0.086 | 0.265 |
| Dihydroceramides | 0.241 | 0.134 | 0.313 |
| Indoles Derivatives | -0.248 | 0.123 | 0.313 |
| Ceramides | 0.227 | 0.158 | 0.332 |
| Cholesterol Esters | 0.202 | 0.212 | 0.372 |
| Nucleobases Related | 0.207 | 0.201 | 0.372 |
| Diacylglycerols | 0.185 | 0.252 | 0.408 |
| Alkaloids | -0.121 | 0.457 | 0.685 |
| Hormones | -0.086 | 0.596 | 0.834 |
| Bile Acids | 0.070 | 0.668 | 0.834 |
| Triacylglycerols | -0.068 | 0.675 | 0.834 |
| Sphingomyelins | 0.056 | 0.730 | 0.852 |
| Carboxylic Acids | 0.042 | 0.795 | 0.879 |
| Acylcarnitines | 0.016 | 0.921 | 0.967 |
| Phosphatidylcholines | 0.004 | 0.979 | 0.979 |

Spearman correlation coefficients (ρ, rho) with FDR-adjusted p-values.
