# Supplementary material for: Targeted metabolomics of postmortem human cardiac tissue using the Biocrates MxP Quant 500 kit
Source: PLoS One. 2026 Aug 19;21(8):e0356269. doi: 10.1371/journal.pone.0356269 (PMC13489478; doi:10.1371/journal.pone.0356269)
Supplement: Supplementary file 4 [file pone.0356269.s005.docx]

## S4 Table. PCA Eigenvalues and Explained Variance (PC1–10)

| **Principal Component** | **Eigenvalue** | **Explained Variance (%)** | **Cumulative Variance (%)** |
| --- | --- | --- | --- |
| PC1 | 152.56 | 32.95 | 32.95 |
| PC2 | 57.51 | 12.42 | 45.37 |
| PC3 | 55.41 | 11.97 | 57.34 |
| PC4 | 27.06 | 5.85 | 63.18 |
| PC5 | 21.87 | 4.72 | 67.91 |
| PC6 | 17.78 | 3.84 | 71.75 |
| PC7 | 14.48 | 3.13 | 74.88 |
| PC8 | 12.83 | 2.77 | 77.65 |
| PC9 | 9.60 | 2.07 | 79.72 |
| PC10 | 8.40 | 1.81 | 81.53 |

## S3.1 Table. Top Contributing Metabolite per Principal Component (PC1–10)

| **Principal Component** | **Top Contributing Metabolite** | **Contribution (%)** |
| --- | --- | --- |
| PC1 | TG 18:1_34:2 | 0.63 |
| PC2 | FA 20:3 | 1.40 |
| PC3 | PC 40:6 | 1.45 |
| PC4 | Cer d18:2/22:0 | 1.74 |
| PC5 | PC 28:1 | 1.78 |
| PC6 | DG 16:0_18:2 | 1.89 |
| PC7 | Cer d18:1/26:0 | 2.47 |
| PC8 | PC 24:0 | 2.24 |
| PC9 | Kynurenine | 3.07 |
| PC10 | Hex3Cer d18:1/16:0 | 4.50 |
