# Supplementary material for: Targeted metabolomics of postmortem human cardiac tissue using the Biocrates MxP Quant 500 kit
Source: PLoS One. 2026 Aug 19;21(8):e0356269. doi: 10.1371/journal.pone.0356269 (PMC13489478; doi:10.1371/journal.pone.0356269)
Supplement: Supplementary file 5 [file pone.0356269.s006.docx]

# S5 Table. Dunn's Post Hoc Test Results for Significant Metabolites

| **Metabolite** | **Comparison** | **Z** | **Unadjusted p-value** | **FDR adjusted p-value** |
| --- | --- | --- | --- | --- |
| C3 | Control - T2D | 0.115 | 0.909 | 1.000 |
| C3 | Control - IHD | -2.695 | 0.007 | 0.021 |
| C3 | T2D - IHD | -2.827 | 0.005 | 0.014 |
| PC O-34:3 | Control - T2D | 3.042 | 0.002 | 0.007 |
| PC O-34:3 | Control - IHD | 1.116 | 0.265 | 0.794 |
| PC O-34:3 | T2D - IHD | -2.397 | 0.017 | 0.050 |
| GABA | Control - T2D | 2.841 | 0.004 | 0.013 |
| GABA | Control - IHD | 2.071 | 0.038 | 0.115 |
| GABA | T2D - IHD | -1.209 | 0.227 | 0.680 |
| TG 22:6_32:1 | Control - T2D | 2.735 | 0.006 | 0.019 |
| TG 22:6_32:1 | Control - IHD | 1.027 | 0.304 | 0.913 |
| TG 22:6_32:1 | T2D - IHD | -2.131 | 0.033 | 0.099 |
| PC O-36:4 | Control - T2D | 2.765 | 0.006 | 0.017 |
| PC O-36:4 | Control - IHD | 1.784 | 0.074 | 0.223 |
| PC O-36:4 | T2D - IHD | -1.409 | 0.159 | 0.477 |
| TG 20:5_34:1 | Control - T2D | 2.391 | 0.017 | 0.050 |
| TG 20:5_34:1 | Control - IHD | 0.254 | 0.800 | 1.000 |
| TG 20:5_34:1 | T2D - IHD | -2.507 | 0.012 | 0.037 |
| C0 | Control - T2D | 2.678 | 0.007 | 0.022 |
| C0 | Control - IHD | 1.458 | 0.145 | 0.435 |
| C0 | T2D - IHD | -1.635 | 0.102 | 0.306 |
| TG 17:2_36:2 | Control - T2D | 1.272 | 0.203 | 0.610 |
| TG 17:2_36:2 | Control - IHD | -1.154 | 0.248 | 0.745 |
| TG 17:2_36:2 | T2D - IHD | -2.623 | 0.009 | 0.026 |
| TG 18:1_28:1 | Control - T2D | 2.229 | 0.026 | 0.078 |
| TG 18:1_28:1 | Control - IHD | 0.193 | 0.847 | 1.000 |
| TG 18:1_28:1 | T2D - IHD | -2.380 | 0.017 | 0.052 |
| C2 | Control - T2D | 1.684 | 0.092 | 0.277 |
| C2 | Control - IHD | -0.641 | 0.522 | 1.000 |
| C2 | T2D - IHD | -2.585 | 0.010 | 0.029 |
| SM 34:2 | Control - T2D | -1.855 | 0.064 | 0.191 |
| SM 34:2 | Control - IHD | -2.551 | 0.011 | 0.032 |
| SM 34:2 | T2D - IHD | -0.409 | 0.683 | 1.000 |
| TG 18:1_30:2 | Control - T2D | 2.085 | 0.037 | 0.111 |
| TG 18:1_30:2 | Control - IHD | -0.033 | 0.974 | 1.000 |
| TG 18:1_30:2 | T2D - IHD | -2.441 | 0.015 | 0.044 |
| HArg | Control - T2D | 2.353 | 0.019 | 0.056 |
| HArg | Control - IHD | 2.198 | 0.028 | 0.084 |
| HArg | T2D - IHD | -0.519 | 0.604 | 1.000 |
| TG 18:1_34:4 | Control - T2D | 1.913 | 0.056 | 0.167 |
| TG 18:1_34:4 | Control - IHD | -0.287 | 0.774 | 1.000 |
| TG 18:1_34:4 | T2D - IHD | -2.496 | 0.013 | 0.038 |
| PC O-30:1 | Control - T2D | 2.554 | 0.011 | 0.032 |
| PC O-30:1 | Control - IHD | 1.331 | 0.183 | 0.550 |
| PC O-30:1 | T2D - IHD | -1.618 | 0.106 | 0.317 |
| TG 18:2_30:1 | Control - T2D | 1.808 | 0.071 | 0.212 |
| TG 18:2_30:1 | Control - IHD | -0.381 | 0.703 | 1.000 |
| TG 18:2_30:1 | T2D - IHD | -2.468 | 0.014 | 0.041 |
| C18:2 | Control - T2D | 1.377 | 0.168 | 0.505 |
| C18:2 | Control - IHD | -0.906 | 0.365 | 1.000 |
| C18:2 | T2D - IHD | -2.496 | 0.013 | 0.038 |
| TG 16:1_36:2 | Control - T2D | 1.884 | 0.060 | 0.179 |
| TG 16:1_36:2 | Control - IHD | -0.248 | 0.804 | 1.000 |
| TG 16:1_36:2 | T2D - IHD | -2.424 | 0.015 | 0.046 |
| PC 32:3 | Control - T2D | 2.449 | 0.014 | 0.043 |
| PC 32:3 | Control - IHD | 1.149 | 0.251 | 0.752 |
| PC 32:3 | T2D - IHD | -1.679 | 0.093 | 0.280 |
