# Supplementary material for: Targeted metabolomics of postmortem human cardiac tissue using the Biocrates MxP Quant 500 kit
Source: PLoS One. 2026 Aug 19;21(8):e0356269. doi: 10.1371/journal.pone.0356269 (PMC13489478; doi:10.1371/journal.pone.0356269)
Supplement: Supplementary file 6 [file pone.0356269.s007.docx]

# S6 Table. Fold change and p-values for metabolites presented in Volcano plots

| Metabolite | Log2 Fold Change | p-value | Comparison | Direction |
| --- | --- | --- | --- | --- |
| **3-IAA** | **-0.63** | **0.010000** | **IHD vs Control** | **Downregulated** |
| **C3** | **1.05** | **0.016500** | **IHD vs Control** | **Upregulated** |
| **HArg** | **-0.64** | **0.036600** | **IHD vs Control** | **Downregulated** |
| **C5-DC (C6-OH)** | **-0.76** | **0.040800** | **IHD vs Control** | **Downregulated** |
| **PC O-34:3** | **-1.04** | **0.000765** | **T2D vs Control** | **Downregulated** |
| **C0** | **-0.80** | **0.004570** | **T2D vs Control** | **Downregulated** |
| **PC 34:3** | **-0.65** | **0.007280** | **T2D vs Control** | **Downregulated** |
| **GABA** | **-0.82** | **0.009110** | **T2D vs Control** | **Downregulated** |
| **TG 20:5_34:1** | **-1.04** | **0.011300** | **T2D vs Control** | **Downregulated** |
| **PC O-36:3** | **-0.60** | **0.013900** | **T2D vs Control** | **Downregulated** |
| **TG 22:6_32:1** | **-1.50** | **0.014000** | **T2D vs Control** | **Downregulated** |
| **HArg** | **-0.74** | **0.015500** | **T2D vs Control** | **Downregulated** |
| **PC 32:3** | **-0.83** | **0.017200** | **T2D vs Control** | **Downregulated** |
| **PC 32:2** | **-0.72** | **0.025700** | **T2D vs Control** | **Downregulated** |
| **TG 14:0_36:1** | **-0.83** | **0.037600** | **T2D vs Control** | **Downregulated** |
| **TG 18:1_28:1** | **-1.19** | **0.037600** | **T2D vs Control** | **Downregulated** |
| **TG 22:6_34:2** | **-1.08** | **0.037600** | **T2D vs Control** | **Downregulated** |
| **TG 20:1_32:2** | **-1.00** | **0.045200** | **T2D vs Control** | **Downregulated** |
| **C3** | **-1.09** | **0.002230** | **T2D vs IHD** | **Downregulated** |
| **C2** | **-0.79** | **0.010700** | **T2D vs IHD** | **Downregulated** |
| **TG 18:1_34:4** | **-1.28** | **0.012100** | **T2D vs IHD** | **Downregulated** |
| **TG 17:2_36:2** | **-0.92** | **0.012900** | **T2D vs IHD** | **Downregulated** |
| **TG 18:1_30:2** | **-1.46** | **0.013700** | **T2D vs IHD** | **Downregulated** |
| **TG 16:1_36:2** | **-1.23** | **0.015500** | **T2D vs IHD** | **Downregulated** |
| **TG 18:2_30:1** | **-1.39** | **0.015500** | **T2D vs IHD** | **Downregulated** |
| **TG 18:1_28:1** | **-1.11** | **0.016500** | **T2D vs IHD** | **Downregulated** |
| **TG 18:1_32:3** | **-1.36** | **0.016500** | **T2D vs IHD** | **Downregulated** |
| **TG 18:0_30:1** | **-1.11** | **0.018500** | **T2D vs IHD** | **Downregulated** |
| **TG 20:5_34:1** | **-0.97** | **0.018600** | **T2D vs IHD** | **Downregulated** |
| **TG 16:0_34:4** | **-1.41** | **0.019700** | **T2D vs IHD** | **Downregulated** |
| **TG 18:1_36:2** | **-0.91** | **0.020900** | **T2D vs IHD** | **Downregulated** |
| **TG 16:0_28:2** | **-1.15** | **0.020900** | **T2D vs IHD** | **Downregulated** |
| **TG 18:1_26:0** | **-0.98** | **0.023400** | **T2D vs IHD** | **Downregulated** |
| **C18:2** | **-0.99** | **0.023500** | **T2D vs IHD** | **Downregulated** |
| **TG 18:1_30:1** | **-1.32** | **0.023500** | **T2D vs IHD** | **Downregulated** |
| **C4** | **-0.71** | **0.024800** | **T2D vs IHD** | **Downregulated** |
| **TG 14:0_36:3** | **-1.18** | **0.024800** | **T2D vs IHD** | **Downregulated** |
| **TG 16:1_36:1** | **-0.95** | **0.024800** | **T2D vs IHD** | **Downregulated** |
| **TG 22:6_32:1** | **-1.11** | **0.026300** | **T2D vs IHD** | **Downregulated** |
| **TG 18:0_32:2** | **-1.03** | **0.027800** | **T2D vs IHD** | **Downregulated** |
| **TG 18:3_32:1** | **-1.40** | **0.029400** | **T2D vs IHD** | **Downregulated** |
| **TG 14:0_36:2** | **-1.06** | **0.029400** | **T2D vs IHD** | **Downregulated** |
| **TG 16:0_28:1** | **-1.48** | **0.029400** | **T2D vs IHD** | **Downregulated** |
| **TG 16:1_30:1** | **-1.52** | **0.029400** | **T2D vs IHD** | **Downregulated** |
| **TG 17:2_36:3** | **-0.83** | **0.029400** | **T2D vs IHD** | **Downregulated** |
| **TG 18:0_34:3** | **-0.96** | **0.031100** | **T2D vs IHD** | **Downregulated** |
| **PC O-34:3** | **-0.65** | **0.032800** | **T2D vs IHD** | **Downregulated** |
| **TG 18:1_32:1** | **-1.19** | **0.032800** | **T2D vs IHD** | **Downregulated** |
| **TG 18:1_32:2** | **-1.26** | **0.032800** | **T2D vs IHD** | **Downregulated** |
| **CA** | **1.62** | **0.032900** | **T2D vs IHD** | **Upregulated** |
| **TG 17:1_34:3** | **-1.15** | **0.032900** | **T2D vs IHD** | **Downregulated** |
| **TG 16:0_32:3** | **-1.28** | **0.034700** | **T2D vs IHD** | **Downregulated** |
| **TG 16:1_34:1** | **-1.28** | **0.034700** | **T2D vs IHD** | **Downregulated** |
| **TG 14:0_34:3** | **-1.43** | **0.036600** | **T2D vs IHD** | **Downregulated** |
| **TG 16:1_32:2** | **-1.47** | **0.036600** | **T2D vs IHD** | **Downregulated** |
| **TG 20:1_32:1** | **-1.02** | **0.038600** | **T2D vs IHD** | **Downregulated** |
| **TG 18:2_32:1** | **-1.25** | **0.040800** | **T2D vs IHD** | **Downregulated** |
| **TG 20:1_30:1** | **-1.01** | **0.040800** | **T2D vs IHD** | **Downregulated** |
| **TG 17:1_32:1** | **-0.94** | **0.042900** | **T2D vs IHD** | **Downregulated** |
| **TG 16:0_38:7** | **-1.10** | **0.043000** | **T2D vs IHD** | **Downregulated** |
| **TG 16:1_34:2** | **-1.45** | **0.043000** | **T2D vs IHD** | **Downregulated** |
| **TG 16:0_34:3** | **-1.30** | **0.045300** | **T2D vs IHD** | **Downregulated** |
| **TG 16:1_36:4** | **-1.31** | **0.045300** | **T2D vs IHD** | **Downregulated** |
| **TG 16:1_32:0** | **-1.08** | **0.045300** | **T2D vs IHD** | **Downregulated** |
| **TG 18:1_34:3** | **-1.23** | **0.045300** | **T2D vs IHD** | **Downregulated** |
| **TG 16:1_34:3** | **-1.61** | **0.045300** | **T2D vs IHD** | **Downregulated** |
| **TG 18:0_34:2** | **-0.87** | **0.045300** | **T2D vs IHD** | **Downregulated** |
| **TG 16:1_32:1** | **-1.47** | **0.047700** | **T2D vs IHD** | **Downregulated** |
| **TG 16:0_32:2** | **-1.20** | **0.047700** | **T2D vs IHD** | **Downregulated** |
