# Supplementary material for: Targeted metabolomics of postmortem human cardiac tissue using the Biocrates MxP Quant 500 kit
Source: PLoS One. 2026 Aug 19;21(8):e0356269. doi: 10.1371/journal.pone.0356269 (PMC13489478; doi:10.1371/journal.pone.0356269)
Supplement: Supplementary file 7 [file pone.0356269.s008.docx]

**Materials and methods, Supplementary Information**

**Sample Preparation**

Sample preparation followed the manufacturer's protocol. Briefly, 10 μL of all samples, including tissue homogenate samples, QC samples, calibrators, and zero samples, were added onto the filter of the 96-well plate, which for each well contained all internal standards. The samples were dried under a gentle stream of nitrogen for 30 minutes and subsequently derivatized using 50 µL of a 5 % PITC solution at room temperature for 60 minutes. Subsequently, the plate was dried using a gentle stream of nitrogen for 60 minutes. The extraction of the metabolites was then performed by the addition of 300 µL of 5 mM ammonium acetate in methanol to each well and shaking the plate at 450 rpm for 30 minutes. After centrifugation at 500 × g for 2 minutes to collect the extracts through the filter inserts, 150 µL of each extract was transferred to an LC plate for LC-MS/MS analysis, followed by the addition of 150 µL MilliQ® water per well. For FIA-MS/MS analysis, 10 µL of each extract was transferred to a separate LC plate and diluted with 490 µL of FIA solvent. Both plates were sealed and shaken at 600 rpm for 10 minutes.

**Instrumental Analysis**

Analysis was performed on an ACQUITY UPLC system (Waters, Milford, MA, USA) coupled to a Waters Xevo TQ-XS triple quadrupole mass spectrometer (Waters Corporation, Milford, MA, USA). The instrument parameters, source conditions, and acquisition settings were provided by Biocrates.

LC-MS/MS analyses were conducted using the kit-specific column system and two distinct kit-specific gradients, each lasting 5.8 minutes. Both gradients were run at a flow rate of 0.8 mL per minute and a column temperature of 50 °C. Mobile phase A consisted of MilliQ water with 0.2 percent formic acid, while mobile phase B was composed of 95 percent acetonitrile with 0.2 percent formic acid. The injection volume was 5 and 15 microliters, and the autosampler was maintained at 10 °C.

FIA MS/MS analyses were carried out with an injection volume of 20 microliters on two different methods. Samples were introduced directly into the mass spectrometer, bypassing the column, using a solution consisting of methanol and the FIA mobile phase additive. The total run time for each FIA-MS/MS injection was 3 minutes.

Mass spectrometric detection and quantification for FIA- and LC-MS/MS were carried out based on analyte-specific mass transitions in the MRM mode.

The sample list was prepared in advance, with the study samples randomized using WebIDQ to minimize batch effects. Calibrators and quality control (QC) samples were arranged in a predefined order according to the kit specifications.

**System and suitability testing**

Instrument performance was evaluated using system suitability tests (SST), performed with the LC-MS/MS and FIA-MS/MS test samples included in the Biocrates MxP® Quant 500 kit. These tests were conducted prior to the preparation and analysis of samples. The resulting SST raw data files were uploaded to the WebIDQ software for quality approval by Biocrates Life Sciences.

**Data analysis**

Following LC-MS/MS and FIA-MS/MS acquisition, raw data from the MxP® Quant 500 kit were processed in the cloud-based software, WebIDQ®. WebIDQ performs quantification based on internal standards, applies quality control, and flags concentrations as <LOD, <LLOQ, or >ULOQ (30). The export also includes a complete list of quantified features along with links to Human Metabolome Database entries (HMDB: <https://hmdb.ca/>) (31), aiding downstream annotation, although some metabolite features are missing from the database.

WebIDQ also calculates and exports a set of pre-configured metabolite sums and ratios through its MetaboINDICATOR tool (30). These indicators are designed to assist biological interpretation. Although user-defined indicators can also be created, we did not include MetaboINDICATOR values in our analysis, as they were not directly applicable to our study objectives. For further statistical evaluation, we exported both filtered and unfiltered data as .xlsx files.
