# Supplementary material for: Targeted metabolomics of postmortem human cardiac tissue using the Biocrates MxP Quant 500 kit
Source: PLoS One. 2026 Aug 19;21(8):e0356269. doi: 10.1371/journal.pone.0356269 (PMC13489478; doi:10.1371/journal.pone.0356269)
Supplement: Supplementary file 8 [file pone.0356269.s010.docx]

This manuscript presents a proof-of-concept study evaluating the feasibility of applying the Biocrates Life Sciences MxP® Quant 500 kit to postmortem human cardiac tissue obtained during forensic autopsies. The authors aim to determine whether this standardized targeted metabolomics platform, originally developed for biofluids, can be extended to solid tissue analysis and whether it may support forensic investigations through comparison of metabolomic profiles across different groups of sudden cardiac death

While the topic is relevant and of potential interest to forensic metabolomics, the current manuscript has important methodological limitations that prevent a full assessment of the validity of the conclusions. The following major issues should be addressed:

1. The manuscript aims to evaluate the feasibility and analytical performance of the MxP® Quant 500 kit in postmortem cardiac tissue. However, key analytical validation parameters (e.g., precision, recovery, matrix effects, limits of detection, and QC performance) are not reported, and no clear optimization of tissue-specific extraction or processing is described. In the absence of these data, the analytical suitability of the platform for postmortem cardiac tissue cannot be fully assessed. Consequently, the absence of statistically significant metabolite differences between cause-of-death groups cannot be confidently interpreted as reflecting biological variation alone.
2. It would be helpful to clarify the rationale for selecting a biofluid-optimized kit for the analysis of solid tissue samples. The manuscript could be strengthened by including a more detailed description of the tissue-specific sample preparation and extraction procedures, including the homogenization method, extraction workflow, and normalization strategy, to support reproducibility of the analytical approach. In addition, reporting missing data rates and the number of metabolites excluded due to QC criteria would further improve the evaluation of platform performance in this matrix. If no specific optimization was performed, this should be clearly stated, along with a brief discussion of the potential implications for data interpretation.
3. It may be worth clarifying whether signal saturation was observed for any compounds, and if so, whether this influenced considerations regarding optimization for cardiac tissue using this kit. Although the study employs a broad targeted metabolomics/lipidomics platform, it should be noted that key mitochondrial phospholipids such as cardiolipins, which are highly abundant and functionally important in cardiac tissue, are not covered by the MxP® Quant 500 kit. This limitation should be acknowledged when interpreting the biological scope of the lipidomic findings, particularly in relation to mitochondrial function and energy metabolism.
4. Given that the study primarily aims to assess the feasibility and analytical performance of the kit, the interpretation of cohort-based comparisons would benefit from prior or concurrent analytical validation and optimization of the platform in postmortem cardiac tissue. In addition, comparison with an appropriate biofluid control, analyzed using the same platform, could provide useful context regarding how the kit performs in its original application relative to cardiac tissue. Furthermore, a summary table comparing metabolite coverage in this study with that reported in previous studies, if available in the literature, would help contextualize the applicability and analytical scope of the current approach.
5. The manuscript should clearly distinguish between the analytical feasibility of the metabolomics platform in postmortem cardiac tissue and the biological or forensic interpretation of metabolite differences between cause-of-death groups. At present, these objectives appear to be partially conflated. Instead of presenting the study primarily as an analytical performance evaluation, the authors may consider reframing the scope to more clearly reflect a biologically exploratory or proof-of-concept application.

Overall assessment

The manuscript presents an interesting attempt to extend a standardized targeted metabolomics platform to postmortem cardiac tissue. However, it currently lacks essential analytical validation and tissue-specific optimization data. Without these, the biological comparisons between cause-of-death groups cannot be reliably interpreted, and conclusions regarding forensic applicability are not sufficiently supported.

Strengthening the analytical validation section and clearly separating methodological feasibility from biological interpretation would substantially improve the scientific robustness and clarity of the manuscript.
