# Supplementary material for: Targeted metabolomics of postmortem human cardiac tissue using the Biocrates MxP Quant 500 kit
Source: PLoS One. 2026 Aug 19;21(8):e0356269. doi: 10.1371/journal.pone.0356269 (PMC13489478; doi:10.1371/journal.pone.0356269)
Supplement: Supplementary file 9 [file pone.0356269.s011.docx]

**Response to Editor and Reviewers**

Manuscript ID: PONE-D-26-02704
Title: *Targeted metabolomics of postmortem human cardiac tissue using the Biocrates MxP® Quant 500 kit*

Dear Editor and Reviewers,

We would like to thank the editor and reviewers for their thorough and constructive evaluation of our manuscript. We have carefully revised the manuscript in response to all comments, and we believe that these revisions have strengthened the manuscript. In particular, we have clarified the proof-of-concept nature of the study, strengthened the statistical reporting, and expanded the analysis of postmortem interval (PMI).

For an overview of each comment and its changes, please see below. The the changed lines we refer to can be found in the tracked version.

**Reviewer #1**

**Major Comment 1: Study aims and narrative clarity**

**Comment:** *The manuscript appears to address two distinct objectives: (1) evaluating the technical feasibility of a targeted metabolomics platform in postmortem cardiac tissue, and (2) assessing biological differences between IHD, T2D, and control groups. At present, the balance between these aims is unclear, resulting in a fragmented narrative. The authors should clarify the primary focus of the study and align the narrative and interpretation accordingly, as the current presentation risks overextending biological interpretation from what is inherently a methodological proof-of-concept study.*

**Response:**
We agree with the reviewer that the original manuscript did not clearly distinguish between the primary methodological aim and the exploratory biological comparisons. We have therefore revised the manuscript to clearly position the study as a proof-of-concept feasibility investigation of the Biocrates MxP® Quant 500 kit applied to postmortem human cardiac tissue.

Biological comparisons between the study groups are now explicitly framed as exploratory and hypothesis-generating. We have toned down or removed language suggesting clear group discrimination of the metabolites and clarified that observed group differences were nominal findings based on unadjusted p-values and did not remain statistically significant after FDR correction.

These revisions have been made throughout the manuscript, including the Abstract (lines 21–51), Introduction (lines 120–128), Results (lines 313–375), Discussion (lines 530–590), and Conclusions (lines 611–626).

**Major Comment 2**

**Comment:** *The implementation of distance-based classification methods (maximum, centroid, Mahalanobis) alongside PLS-DA is unclear. It is not evident whether these approaches were applied within the PLS-DA latent space or used as independent classifiers. In addition, no model validation metrics (e.g., Q², permutation testing, or cross-validation strategy) are reported. This raises concerns regarding potential overfitting and the robustness of the reported classification performance.*

**Response:**
**Reviewer #1** and **#2** both raised concerns regarding the use of the PLS-DA and how it was presented in the original manuscript, and we agree with these concerns. Based on the reviewers’ input we decided to remove the PLS-DA altogether from the manuscript. The PLS-DA did not provide main findings for the study, and the analysis is not well suited for this type of data.

This includes removal of the description of PLS-DA from the Statistical analysis section of the Materials and Methods (lines 242–245), removal of the PLS-DA results ( lines 329–351), and removal of the interpretation from the Discussion (lines 506–512).

Eliminating it reduces the risk of overextending biological interpretation of the results – thus further addressing other comments from reviewer 1 (Major comment #1) and reviewer 2 (Comment A).

**Major Comment 3**

**Comment:** *The reporting of FDR-adjusted p-values is unclear. The statement that all metabolites have an identical adjusted p-value (p = 0.628) appears implausible, as Benjamini–Hochberg correction typically produces a distribution of adjusted p-values. The authors should clarify how FDR correction was implemented and how these values are being reported.*

**Response:**
We have clarified the implementation of the Benjamini–Hochberg false discovery rate (FDR) correction in the Method section and revised the Result section to improve clarity. FDR correction was applied across all retained metabolites tested in the Kruskal–Wallis analysis, treating them as a single set of simultaneous statistical tests. We now report FDR-adjusted p-values alongside raw p-values in Table 3 and explicitly state that no metabolites remained significant after correction. The wording has been revised to avoid implying that all metabolites had an identical adjusted p-value.

These changes have been made in the Methods section (lines 222-224), Results section (lines 355-360), and Table 3. Further all p-values that were reported as being statistically significant, have now been changed to being reported as nominally significant.

**Major Comment 4**

**Comment:** *The PERMANOVA results indicate no significant differences in overall metabolite composition between groups (R² = 0.051, p = 0.429), which is not consistent with the apparent separation suggested by the PLS-DA analysis. This discrepancy should be addressed. Furthermore, it is not stated whether homogeneity of dispersion was assessed, which is a key assumption underlying PERMANOVA.*

**Response:**
We acknowledge this discrepancy and appreciate the reviewer’s concern. We have addressed this by explicitly reporting the non-significant PERMANOVA results and by adding a test for homogeneity of multivariate dispersion (PERMDISP). As the PLS-DA analysis has now been removed from the revised manuscript, the previous discrepancy between PERMANOVA and PLS-DA no longer applies. We also believe that adding PERMDISP provides additional interpretational value to the dataset. The Results section now clarifies that the observed variation primarily reflects within-group heterogeneity rather than clear group separation.

These revisions have been incorporated into the Methods section (lines 229-231), Results section (lines 370-373), as well as the lines related to PLS-DA, as described in the response to Major Comment 2.

**Major Comment 5**

**Comment:** *Table 3 presents metabolites selected on the basis of unadjusted p-values (< 0.05), despite the application of FDR correction indicating that none remain statistically significant. Presenting these features without adjusted p-values risks overstating their relevance. Adjusted p-values should be included alongside raw p-values for transparency of reporting.*

**Response:**
As described in the response to Major Comment 3, we have revised Table 3 to include both unadjusted and FDR-adjusted p-values. We have also revised the Results section to explicitly describe these findings as nominal (unadjusted) and exploratory, as no metabolites remained statistically significant after FDR correction.

These revisions are included in the Results section (lines 384 for Table 3).

**Major Comment 6**

**Comment:** *The PLS-DA visualization uses convex hulls to outline group boundaries; however, these are sensitive to extreme observations. The broad hulls observed—particularly for the IHD and T2D groups—suggest that the apparent separation may be influenced by a small number of outliers rather than consistent clustering. This raises concerns about overinterpretation, and the influence of outliers on the model should be assessed or discussed appropriately.*

**Response:**
We agree with the reviewer’s concern. The PLS-DA analysis has been removed from the revised manuscript. This removes the risk of overinterpreting apparent group boundaries driven by outliers

These changes are similar to those described in response to Major Comment 2.

**Major Comment 7**

**Comment:** *Although the PMI range (3–8 days) is described as relatively narrow, metabolite levels are known to change over this interval in humans. In the context of a feasibility study, PMI should be considered not only as a confounder but also as a variable of interest. Assessing how metabolite detection or quantification rates vary with PMI would strengthen the study and provide insight into the robustness of the assay under postmortem conditions.*

**Response:**
We thank the reviewer for this important comment and agree that PMI should be considered not only as a potential confounder but also as a variable of interest in a feasibility study. We have therefore expanded the analysis of PMI as follows:

- Performed Spearman correlation analyses between PMI and metabolite class scores based on z-score normalized concentrations.
- Added PCA plots colored by PMI, now included as Fig S1
- Summarized the results in the main text and S2 Table

These analyses showed that PMI contributes to variability in certain metabolite classes. However, the observed associations were generally weak to moderate and did not remain statistically significant after FDR correction. PCA likewise did not reveal clear clustering by PMI. We have also clarified that PMI is an uncertain proxy for postmortem degradation, as degradation is influenced by factors such as temperature, humidity, storage conditions, and environmental exposure. Therefore, visible putrefaction was used as an additional pragmatic exclusion criterion to limit bias from advanced postmortem degradation.

Taken together, these revisions indicate that, within the studied PMI range, the PMI was not a dominant factor influencing the overall metabolite profile.

These revisions have made in the Introduction (lines 84–90), Methods section (lines 155–156, 159-166 and 232–236), Results section (lines 306–311, 318-322), Discussion (lines 490-498).

**Minor Comment 1**

**Comment:** *The proof-of-concept nature of the study is stated in the main text but would benefit from being more clearly indicated in the title and/or* ***abstract*** *to appropriately frame the scope and expectations of the work.*

**Response:** We have clarified the proof-of-concept nature of the study in the Abstract, including the first sentence describing the study aim. We believe the title already reflects the methodological focus of the study, and therefore we did not deem a title change necessary.

This revision has been made in the Abstract (lines 22–24).

**Minor Comment 2**

**Comment:** *There is an inconsistency in the definition of ischemic heart disease (IHD) between the Methods and Results. In the Methods, IHD is defined as ≥75% coronary artery stenosis, whereas in the Results, most IHD cases are described as having ≥50% stenosis. This discrepancy should be clarified and corrected.*

**Response:** We have clarified this discrepancy by revising Table 1 and expanding the reporting of coronary artery stenosis. The other coronary arteries have now been added to Table 1, and stenosis degree has been stratified as mild, moderate, moderately severe, and severe. We also added the group comparison tests to the Methods section and updated the Results text accordingly.

These revisions have been made in the Methods section (lines 174–177 and 219–221), Results section (lines 257–275), and Table 1.

**Minor Comment 3**

**Comment:** *In the Discussion, the authors state that the study groups were broadly matched; however, no statistical comparisons of baseline characteristics are presented to support this claim. Appropriate between-group comparisons should be included, or the statement should be revised.*

**Response:** We thank the reviewer for this comment. We have revised the manuscript to address this point by including statistical comparisons of baseline characteristics between groups, which are now presented in Table 1 with corresponding p‑values calculated using Kruskal–Walli’s test. Based on these analyses, the groups were comparable with respect to age, BMI, PMI, heart weight, and left ventricular wall thickness, while expected differences were observed for coronary artery stenosis. The text has been updated accordingly to ensure that statements regarding group comparability are supported by the reported statistical analyses.

**Minor Comment 4**

**Comment:** *Figure 2 caption appears to retain “tracked changes” from multiple authors, please finalise this caption accordingly.*

**Response:** We thank the reviewer for noticing this. The Fig 2 caption has now been finalized, and all figure legends have been reviewed and inserted into the manuscript.

**Minor Comment 5:**

**Comment:** *The corresponding author does not match between the manuscript submission system and the written manuscript version, please clarify accordingly.*

**Response:**  This has now been corrected so that the corresponding author information is consistent between the manuscript and the submission system. We thank the reviewer for pointing this out.

**Reviewer #2**

**Comment A)**
*The manuscript requires a clear conceptual repositioning. At present, it simultaneously presents itself as a feasibility/methodological study and as a biological comparison between T2D, IHD, and control groups. The data do not support the latter. The absence of FDR-significant metabolites, the non-significant PERMANOVA, and the weak multivariate separation indicate that no robust biological discrimination has been demonstrated. The authors should explicitly reframe the study as a feasibility investigation and revise the title, abstract, and conclusions accordingly. Any biological findings should be clearly downgraded to exploratory observations, and all language suggesting group discrimination should be removed or substantially toned down.*

**Response:** We agree fully with the reviewer’s assessment and have addressed this concern in parallel with Reviewer #1, Major Comment 1, which raised a closely related issue regarding the clarity of the study’s aims and narrative focus. The manuscript has now been comprehensively reframed as a feasibility‑focused, methodological proof‑of‑concept study.

Accordingly, the Abstract, Results, Discussion, and Conclusions have all been revised to clearly reflect this position. Biological comparisons between T2D, IHD, and control groups are now explicitly described as exploratory, and all language suggesting robust group discrimination has been removed or substantially toned down. We also explicitly state that no metabolites remained significant after FDR correction and that PERMANOVA did not show significant group differences. We consider the absence of clear group discrimination to be an important feasibility-related finding, as it highlights the limitations of exploratory biological interpretation in this sample set.

These revisions have been made in the Abstract (lines 21-26, 45-49), Results (lines 319-322, 356-365, 366-373), Discussion (lines 504-538, with removal of lines 539-564), and Conclusions (lines 588–600), as also described in the responses to Reviewer #1, Major Comments 1–5.

**Comment B)** *The impact of postmortem interval (PMI) is a central issue that is currently underdeveloped and must be addressed more rigorously. The reported PMI range (3–8 days) is biologically relevant and likely to influence metabolite stability. Moreover, the apparent imbalance between groups (with longer PMI in controls) introduces a potential systematic bias. The authors should explicitly investigate PMI as a confounding factor, for example by assessing correlations between PMI and major metabolite classes, visualizing multivariate analyses colored by PMI, or discussing the stability of results across the PMI range. This is particularly important given that existing forensic metabolomics literature has consistently identified PMI as a dominant driver of metabolomic variability, often exceeding disease-related effects and requiring explicit standardization strategies.*

**Response:** This was concern was also addressed by reviewer #1 and we agree that it requires more attention. We now made revisions to explicitly evaluate the PMI by performing Spearman correlation analyses between PMI and metabolite class scores based on z‑score–normalized concentrations, and by adding PCA visualizations colored by PMI to assess potential PMI‑related structure in the data. The statistical approach is described in the Methods section, and PMI‑related results are reported in the main text and in S2 Table.

After re‑examining the dataset, the reported PMI range was corrected from 3–8 days to 3–9 days, and all PMI‑related descriptions have been updated accordingly. We have also expanded the Introduction to clarify that PMI is only a proxy measure for postmortem degradation, as degradation is influenced by factors such as temperature, humidity, storage conditions, and environmental exposure. For this reason, visible signs of putrefaction were used as an additional pragmatic exclusion criterion to limit bias from advanced postmortem degradation.

The results show that while PMI contributes to variability in certain metabolite classes, the associations were generally weak to moderate and did not remain statistically significant after FDR correction. PCA likewise did not reveal clear clustering by PMI. Taken together, these findings suggest that, within the studied PMI range of 3–9 days, PMI was not a dominant factor influencing the overall metabolite profile.

These revisions have been made in the Introduction (lines 83–90), Methods section (lines 153–166 and 232–236), Results section (lines 306–311, 318-320), and added Supporting Information descriptions for S1 Fig and S2 Table (lines 744-750, 754–756).

**Comment C)** *The heterogeneity of the study groups significantly limits interpretability and should be explicitly acknowledged and incorporated into the discussion. The T2D group includes individuals with heterogeneous causes of death (including traumatic and ischemic conditions), while the IHD group includes both acute and chronic cases. This creates overlapping biological categories and likely explains the substantial overlap observed in PCA and other analyses. The authors should revise the interpretation to reflect that group comparisons are intrinsically limited by this design and should be considered hypothesis-generating only.*

**Response:** We agree with the reviewer and now explicitly acknowledge group heterogeneity as a key limitation of the study. The Results and Discussion have been revised to clarify that the T2D group included heterogeneous causes of death and that the IHD group included both acute and chronic ischemic cases. We also emphasize that these overlapping biological categories likely contributed to the observed overlap in PCA.

Accordingly, all group comparisons are now interpreted as exploratory and hypothesis-generating.

These revisions have been made in the Results related to PERMANOVA and PERMDISP (lines 366-373) and Discussion section (lines 504–514). This was also previously addressed in the Limitations section (lines 567–570).

**Comment D)** *The choice of analytical platform and its adaptation to postmortem tissue requires a more in-depth and central discussion. The use of a metabolomics kit validated for biofluids introduces well-known issues when applied to solid postmortem tissues, including matrix effects, extraction variability, and dynamic range mismatch. The observed limitations (e.g., saturation of certain metabolite classes and low detectability of others) should be treated as core findings of the feasibility analysis rather than secondary remarks. In this context, it would be useful for the authors to frame their observations within the broader literature, where the choice of biological matrix and its postmortem stability has been shown to critically influence metabolomic outputs and their forensic interpretability.*

**Response:**

We agree that the analytical platform and its adaptation to postmortem cardiac tissue should be treated as central to the feasibility assessment. The introduction addressed this in the lines 108-129. The Discussion already addressed the class-dependent performance of the MxP® Quant 500 kit, including the number of retained metabolites, the use of the vendor-recommended extraction protocol with a fixed 10 μL input volume, low detectability of some metabolite classes, and saturation of abundant classes such as amino acids and fatty acids.

We have now expanded this section to further emphasize these observations as core feasibility findings. Specifically, we added discussion of LOD/2 imputation and its potential to reduce variance and influence downstream statistics in classes with many values below LOD, which we elaborate on in response to **Comment F**. We also expanded the discussion of postmortem-specific effects, as described in response to **Comment B** and Reviewer #1’s **Major Comment 7.**

These revisions clarify that the observed LOD/ULOQ patterns, imputation-related limitations, and postmortem effects are central findings of the feasibility assessment.

**Comment E)** *The statistical analysis, while based on appropriate methods, is not sufficiently rigorous in its interpretation. In particular, the use of PLS-DA in a small sample size/high-dimensional setting carries a substantial risk of overfitting. The authors should explicitly acknowledge this limitation and avoid any implication of discriminatory performance. If retained, PLS-DA results should be clearly presented as exploratory, and their limitations should be emphasized. Additionally, the interpretation of metabolites showing nominal trends but no significance after FDR correction should be explicitly framed as non-conclusive.*

**Response:** As described in response to **Reviewer #1**’s **Major Comment 2** and **6**, the PLS-DA analysis and all associated classification metrics have been removed from the revised manuscript to avoid any implication of discriminatory or predictive performance.

In addition, as described in response to Reviewer #1’s **Major Comments 3** and **5**, metabolites showing nominal differences are now explicitly framed as exploratory and non-conclusive, as no metabolites remained statistically significant after FDR correction.

**Comment F)** The handling of values below the limit of detection should be more critically discussed. The use of imputation strategies such as LOD/2 is common, but in this dataset—where entire metabolite classes are affected—it may influence downstream statistical analyses. The authors should discuss the potential impact of this choice and its implications for the robustness of their findings.

**Response:** We have expanded the Discussion to explicitly address the handling of values below the limit of detection and the implications of LOD/2 imputation. The revised text explains that LOD/2 imputation was used to enable inclusion of all samples in downstream univariate and multivariate analyses, while metabolites consistently below LOD were excluded by the 75% quantification filter.

We also now explicitly acknowledge that, because several metabolite classes contained a high proportion of values below LOD, LOD/2 imputation may reduce variance and influence downstream statistics. Results for these metabolite classes should therefore be interpreted cautiously.

This revision has been made in the Discussion section (lines 467–474).

**Comment G)** The Discussion requires restructuring to better align with the data. The conclusions should focus on what has been convincingly demonstrated, namely the partial analytical feasibility of the approach and the class-dependent performance of the platform. Unsupported biological interpretations should be removed or clearly separated from the main conclusions.

**Response:** We have substantially revised the Discussion to better align with the data and the feasibility-focused scope of the study. The revised Discussion now focuses more clearly on analytical feasibility. Unsupported biological interpretations have been removed or toned down, and metabolites with nominal differences are now discussed as exploratory observations only, as no metabolites remained statistically significant after FDR correction.

Several of these revisions overlap with changes described in previous responses to the reviewers. For example, the Discussion now emphasizes LOD/ULOQ patterns, sample input limitations, and postmortem-specific effects as core feasibility findings. In addition, the section “Metabolic differences among T2D, IHD, and controls” has been markedly reduced and revised to avoid suggesting robust differences between groups.

These revisions have been made throughout the Discussion (lines 467-474, 503–581), as well as in the Conclusion (lines 588–592).

**Conclusion**

We believe these revisions have significantly strengthened the manuscript and addressed all concerns raised by the reviewers. We thank the reviewers again for their constructive feedback.

**Journal Requirements**

**1. Formatting requirements**
We have revised the manuscript to comply with PLOS ONE formatting guidelines, including manuscript structure, figure preparation, file naming, and Supporting Information formatting.

**2-4. Funding information consistency**
Funding-related text has been removed from the manuscript body and is now included only in the designated Funding Statement/submission system fields. We have also included the required role-of-funders statement in the cover letter: “*The funders had no role in study design, data collection and analysis, decision to publish, or preparation of the manuscript*.”

If further changes are required in the online submission system, we would be grateful for guidance from the editorial office, as we were not able to modify all funding-related fields during resubmission.

**5. Data availability statement**
We have revised the Data Availability Statement to clarify which data are publicly available and which data are restricted due to legal and ethical limitations. The processed metabolomics data and statistical scripts are publicly available via GitHub, whereas the anonymized individual-level autopsy metadata cannot be made publicly available, despite being anonymized, due to restrictions governing the use of forensic autopsy material under Danish law.

We would like the Data Availability Statement to include the following clarification:
“*Processed metabolomics data and statistical scripts are publicly available via GitHub. Anonymized individual-level autopsy metadata, including age, body mass index, postmortem interval, heart weight, left ventricular wall thickness, and coronary stenosis, are not publicly available due to legal and ethical restrictions governing the use of forensic autopsy material under Danish law. These metadata represent the complete set of individual-level case information used in the study and are held within a secure institutional database. Requests for data access should be directed to the Data Access Committee at the Department of Forensic Medicine, University of Copenhagen:* [*RI-EDMC@sund.ku.dk*](mailto:RI-EDMC@sund.ku.dk) *and will be considered in accordance with applicable legal, ethical, and institutional requirements.”*

If this statement cannot be updated directly in the online submission system, we would be grateful for editorial assistance.

**6. ORCID**
An ORCiD has now been linked with the current corresponding author. We would also wish to change the corresponding from marie.mardal@uit.no to Oscar.puntervold@sund.ku.dk, if this is possible at this stage.

**7. “Data not shown”**
All instances of “data not shown” have been removed, and the section in where we referred to this have been removed.

**8–10. Figures and Supporting Information**

Figure captions have been revised and inserted separately in the manuscript. All figures have been updated to meet resolution and formatting requirements. Supporting Information captions and in-text references have also been reviewed and updated.

**Protocols.io**
The laboratory workflow followed the manufacturer’s protocol for the Biocrates MxP® Quant 500 kit. We supplied the additional sample preparation details provided in the S1 File. We have therefore not deposited a separate protocol in protocols.io, but we have ensured that the relevant methodological details are included in the manuscript and S1 File.
