# Supplementary material for: Targeted metabolomics of postmortem human cardiac tissue using the Biocrates MxP Quant 500 kit
Source: PLoS One. 2026 Aug 19;21(8):e0356269. doi: 10.1371/journal.pone.0356269 (PMC13489478; doi:10.1371/journal.pone.0356269)
Supplement: Supplementary file 10 [file pone.0356269.s012.docx]

## Response to reviewers round 2

We thank the Editor and the Reviewers for their careful evaluation of our manuscript and for their constructive comments and suggestions. We have revised the manuscript accordingly and provide a detailed, point-by-point response below. Quotes in the rebuttal from modified text are highlighted in orange. Unmodified parts of the quotes are black.

**Reviewer #1**

The authors have addressed my previous major concerns satisfactorily. The manuscript is substantially improved, and the conclusions are now appropriately aligned with the data.

I have only one minor suggestion remaining, that would help the narrative flow: The Introduction could be restructured to better reflect the methodological focus by introducing the feasibility rationale first, with the biological background second serving as motivation rather than the primary narrative.

**Authors Response**: We thank the reviewer for the acknowledgement of our correction. We restructured the introduction (lines: 54-115 - not quoted here due to length) with the feasibility rationale first, which better aligns with the narrative flow of the manuscript.

**Reviewer #2**

Thank you for your careful and constructive revision. The manuscript has improved substantially and addresses the major concerns raised during the previous review rounds. In particular, the study has been appropriately reframed as a proof-of-concept feasibility investigation, the statistical interpretation has been strengthened, and the conclusions are now more closely aligned with the data presented. I have no further substantive comments and consider the manuscript suitable for publication.

**Reviewer #3**

(No Response)

**Reviewer #4**

The authors responded satisfactory to all issues raised. No further experiments or evaluations are required to support message of the manuscript.

**Reviewer #5** (new)

This manuscript presents a proof-of-concept study evaluating the feasibility of applying the Biocrates Life Sciences MxP® Quant 500 kit to postmortem human cardiac tissue obtained during forensic autopsies. The authors aim to determine whether this standardized targeted metabolomics platform, originally developed for biofluids, can be extended to solid tissue analysis and whether it may support forensic investigations through comparison of metabolomic profiles across different groups of sudden cardiac death

While the topic is relevant and of potential interest to forensic metabolomics, the current manuscript has important methodological limitations that prevent a full assessment of the validity of the conclusions. The following major issues should be addressed:

1. The manuscript aims to evaluate the feasibility and analytical performance of the MxP® Quant 500 kit in postmortem cardiac tissue. However, key analytical validation parameters (e.g., precision, recovery, matrix effects, limits of detection, and QC performance) are not reported, and no clear optimization of tissue-specific extraction or processing is described. In the absence of these data, the analytical suitability of the platform for postmortem cardiac tissue cannot be fully assessed. Consequently, the absence of statistically significant metabolite differences between cause-of-death groups cannot be confidently interpreted as reflecting biological variation alone.

**Authors Response**: We thank the reviewer for this suggestion. To strengthening the analytical validation section, we have added a new Supplementary Information table (SI1 Table - shifting the number of the other SI Tables) reporting key analytical performance metrics for all measured metabolites, including many of the variables requested: limits of detection, lower and upper limits of quantitation, QC performance at each concentration level, accuracy assessment, together with precision. These are indeed very important metrics to address analytical suitability, and we should have added it to begin with.

The methodological detail regarding the tissue preparation was not sufficiently clear. We described the sample extraction method in more detail and clarified, with references, why this was selected. The requested optimization of tissue-specific extraction or processing has already, in large, been performed by the referenced paper by Andresen et al. in other postmortem matrices. In the updated method section, we specified what optimization parameters were already covered for postmortem matrices by this paper. An important source of information missing from this Andresen paper is median measured values, and analytical performance - we have this in our manuscript in SI1 and SI2 Tables.

**Modifications**: “The samples were placed in cryotubes and stored at −70°C immediately after the autopsy and later thawed and homogenized at a 1:3 ratio with isopropanol. This single extraction with 100% isopropanol was selected based on the procedure reported by Andresen et al. [10] who evaluated different extraction media and tissue-to-solvent ratios for human tissue analyzed with the same kit. The tissue was homogenized using a gentleMACS Octo Dissociator (Miltenyi Biotec, Bergisch Gladbach, Germany) with a 50–second program applying up to 4000 rpm. The instrumentation is routinely used for postmortem muscle and brain tissue homogenization prior to quantitative analysis in forensic case work with the same tissue-to-solvent ratio, although different extraction media optimized for a different group of analytes [10,34,35]. Approximately 500 mg of tissue was processed per case, and homogenate supernatants were stored at −70°C until further analysis.” (lines: 163-170)

2. It would be helpful to clarify the rationale for selecting a biofluid-optimized kit for the analysis of solid tissue samples. The manuscript could be strengthened by including a more detailed description of the tissue-specific sample preparation and extraction procedures, including the homogenization method, extraction workflow, and normalization strategy, to support reproducibility of the analytical approach. In addition, reporting missing data rates and the number of metabolites excluded due to QC criteria would further improve the evaluation of platform performance in this matrix. If no specific optimization was performed, this should be clearly stated, along with a brief discussion of the potential implications for data interpretation.

**Authors Response**: We thank the reviewer for this valuable suggestion that builds on some of the same points as mentioned in issue #1. The new information we provided in SI1 Table complements the methodological description and provides readers with additional insight into assay performance across the measured metabolite panel. We further included a summary for analytical performance of the metabolites in the results section. There is no commercially available metabolomics kit, to our knowledge, for cardiac tissue, but (as mentioned in the introduction, lines 83-85) this kit has already been used for several alternative matrices. The methodological detail regarding the tissue preparation was not sufficiently clear. We described the sample extraction method in more detail and clarified, with references, why this was selected (se modification under issue #1).

**Modifications**: We here show a snip of SI1 Table to display the variables presented. The new table was referenced at lines 257, 307, 320, and 377. In addition, we added asterisk to Table 3 for metabolites that had at least one QC failed on either coefficient of variation or accuracy.

(lines 305-310): “If we look at the analytical performance of the metabolites with unadjusted p < 0.05 in the Kruskal-Wallis test (Table 3), the triglycerides and PC O-30:1 had at least one failed QC (S1 Table), either for the coefficient of variation or accuracy, measured at three levels with at least three QCs at each level. At least one failed QC for one parameter is indicated with an asterisk in Table 3. The remaining ten metabolites passed all set parameters, with the medium-level QC coefficient of variation below 12% (N = 4).”

3. It may be worth clarifying whether signal saturation was observed for any compounds, and if so, whether this influenced considerations regarding optimization for cardiac tissue using this kit. Although the study employs a broad targeted metabolomics/lipidomics platform, it should be noted that key mitochondrial phospholipids such as cardiolipins, which are highly abundant and functionally important in cardiac tissue, are not covered by the MxP® Quant 500 kit. This limitation should be acknowledged when interpreting the biological scope of the lipidomic findings, particularly in relation to mitochondrial function and energy metabolism.

**Authors Response**: To address concerns related to the analytical measurement range, we have included a Supplementary Information SI1 Table reporting the lower and upper limits of quantitation for all measured metabolites, allowing readers to assess whether measurements approached the validated analytical range. This additional information provides greater transparency regarding the quantitative performance of the assay across the metabolite panel. SI2 Table already has some of the information requested: Here the proportion of values <LOD, <LLOQ, and >ULOQ are presented for the metabolites we measure in our postmortem cardiac tissue. This is particularly pronounced for fatty acids, since these are measured with FIA- and not LC-MS/MS - this additional point was added.

**Modifications**: In the discussion section added a note on cardiolipins (lines 396-398): “A notable group of metabolites not covered by this kit is cardiolipins (important for cardiac mitochondrial function) whose altered metabolism has been associated with cardiac and metabolic disorders [41].” Where 41 is “Bautista JS, Falabella M, Flannery PJ, Hanna MG, Heales SJR, Pope SAS, et al. Advances in methods to analyse cardiolipin and their clinical applications. TrAC - Trends in Analytical Chemistry. Elsevier B.V.; 2022. doi:10.1016/j.trac.2022.116808”

Lines 375-379: “Several classes, such as acylcarnitines and biogenic amines, showed a high proportion of values below the LOD, suggesting that these analytes may have required a greater sample volume to increase the analyte concentration. Conversely, amino acids and fatty acids displayed saturation effects, with > 30% of values exceeding the ULOQ in some cases, indicating that these abundant analytes may benefit from sample dilution. Most fatty acids in this kit are analyzed by FIA-MS/MS, a platform with different analytical characteristics than LC-MS/MS, including dynamic range. This is reflected in a higher number of high- and low-level QCs with CV error in this group (S1 Table); if this group of analytes is of particular interest, a lipidomics method or, alternatively, the MxP Quant 500 XL kit would be a better choice.”

4. Given that the study primarily aims to assess the feasibility and analytical performance of the kit, the interpretation of cohort-based comparisons would benefit from prior or concurrent analytical validation and optimization of the platform in postmortem cardiac tissue. In addition, comparison with an appropriate biofluid control, analyzed using the same platform, could provide useful context regarding how the kit performs in its original application relative to cardiac tissue. Furthermore, a summary table comparing metabolite coverage in this study with that reported in previous studies, if available in the literature, would help contextualize the applicability and analytical scope of the current approach.

**Authors Response**: The metabolomics kit used came with several biofluid control samples (as presented in SI). Unfortunately, NIST does not produce SRM for metabolites in postmortem cardiac tissue. We used extraction-solvent-matched blank samples, and quality control samples at three concentration levels for each metabolite were analyzed. Additionally, some metabolites were quantified with seven-point calibration curves. These were included in our analytical run. With the additional information we provided in SI1 Table, it is now possible to see analytical performance for all metabolites.

We evaluated the suggestion on the additional table summarizing number of metabolites analyzed in other studies. There are excellent hypothesis-driven studies that only quantify a handful of preselected targets, and exploratory untargeted metabolomics studies using metabolites annotated without cerebral oversight reporting metabolites in the thousands. The extensive discussion needed to contextualize this would be better suited for a review-style paper.

5. The manuscript should clearly distinguish between the analytical feasibility of the metabolomics platform in postmortem cardiac tissue and the biological or forensic interpretation of metabolite differences between cause-of-death groups. At present, these objectives appear to be partially conflated. Instead of presenting the study primarily as an analytical performance evaluation, the authors may consider reframing the scope to more clearly reflect a biologically exploratory or proof-of-concept application.

**Authors Response**: The manuscript has been evaluated by a total of five reviewers. To address comments from the first round of reviewers, the scope was more clearly focused on the analytical performance as a proof-of-concept study, and we toned down the biologically exploratory aspects. Reshaping this back would conflict with the approved modifications after first round of review.

**Modifications**: The updated introduction (in response to reviewer #1, lines 54-115) should improve the narrative flow of the manuscript.

Overall assessment

The manuscript presents an interesting attempt to extend a standardized targeted metabolomics platform to postmortem cardiac tissue. However, it currently lacks essential analytical validation and tissue-specific optimization data. Without these, the biological comparisons between cause-of-death groups cannot be reliably interpreted, and conclusions regarding forensic applicability are not sufficiently supported.

Strengthening the analytical validation section and clearly separating methodological feasibility from biological interpretation would substantially improve the scientific robustness and clarity of the manuscript.

**Authors Response**: We thank the reviewer for the valuable comments. We strengthened the section with analytical validation data/run performance data (notably with SI1 Table), clarified what postmortem tissue-specific optimization was performed in a referenced study, in and more clearly separated the methodological feasibility from biological interpretation in the introduction - to align with the order in the manuscript.
