# Supplementary material for: Targeted metabolomics of postmortem human cardiac tissue using the Biocrates MxP Quant 500 kit
Source: PLoS One. 2026 Aug 19;21(8):e0356269. doi: 10.1371/journal.pone.0356269 (PMC13489478; doi:10.1371/journal.pone.0356269)
Supplement: Peer review history

# Peer review: Decision Letter 0

**DOI:** 10.1371/journal.pone.0356269.r001

**Authors:** Xin Sun

4 Apr 2026

Dear Dr. Mardal,

Thank you for submitting your manuscript to PLOS ONE. After careful consideration, we feel that it has merit but does not fully meet PLOS ONE’s publication criteria as it currently stands. Therefore, we invite you to submit a revised version of the manuscript that addresses the points raised during the review process.

Please submit your revised manuscript by May 19 2026 11:59PM. If you will need more time than this to complete your revisions, please reply to this message or contact the journal office at plosone@plos.org. When you're ready to submit your revision, log on to https://www.editorialmanager.com/pone/ and select the 'Submissions Needing Revision' folder to locate your manuscript file.

- A letter that responds to each point raised by the academic editor and reviewer(s). You should upload this letter as a separate file labeled 'Response to Reviewers'.
- A marked-up copy of your manuscript that highlights changes made to the original version. You should upload this as a separate file labeled 'Revised Manuscript with Track Changes'.
- An unmarked version of your revised paper without tracked changes. You should upload this as a separate file labeled 'Manuscript'.

If you would like to make changes to your financial disclosure, please include your updated statement in your cover letter. Guidelines for resubmitting your figure files are available below the reviewer comments at the end of this letter.

If applicable, we recommend that you deposit your laboratory protocols in protocols.io to enhance the reproducibility of your results. Protocols.io assigns your protocol its own identifier (DOI) so that it can be cited independently in the future. For instructions see: https://journals.plos.org/plosone/s/submission-guidelines#loc-laboratory-protocols. Additionally, PLOS ONE offers an option for publishing peer-reviewed Lab Protocol articles, which describe protocols hosted on protocols.io. Read more information on sharing protocols at https://plos.org/protocols?utm_medium=editorial-email&utm_source=authorletters&utm_campaign=protocols.

As the corresponding author, your ORCID iD is verified in the submission system and will appear in the published article. PLOS supports the use of ORCID, and we encourage all coauthors to register for an ORCID iD and use it as well. Please encourage your coauthors to verify their ORCID iD within the submission system before final acceptance, as unverified ORCID iDs will not appear in the published article. *Only*  the individual author can complete the verification step; PLOS staff *cannot*  verify ORCID iDs on behalf of authors.

We look forward to receiving your revised manuscript.

Kind regards,

Xin Sun, PhD

Staff Editor

PLOS One

**Journal Requirements:**

When submitting your revision, we need you to address these additional requirements.

1. Please ensure that your manuscript meets PLOS ONE's style requirements, including those for file naming. The PLOS ONE style templates can be found at

https://journals.plos.org/plosone/s/file?id=wjVg/PLOSOne_formatting_sample_main_body.pdf and

https://journals.plos.org/plosone/s/file?id=ba62/PLOSOne_formatting_sample_title_authors_affiliations.pdf

2. We note that the grant information you provided in the ‘Funding Information’ and ‘Financial Disclosure’ sections do not match.

When you resubmit, please ensure that you provide the correct grant numbers for the awards you received for your study in the ‘Funding Information’ section.

3. Thank you for stating the following financial disclosure:

“This work was supported by the Department of Forensic Medicine, University of Copenhagen, Denmark, by internal funding. Marie Mardal received funding support from the Norwegian Research Council [grant number: 312276].”

Please state what role the funders took in the study.  If the funders had no role, please state: "The funders had no role in study design, data collection and analysis, decision to publish, or preparation of the manuscript."

If this statement is not correct you must amend it as needed.

Please include this amended Role of Funder statement in your cover letter; we will change the online submission form on your behalf.

4. Please note that funding information should not appear in any section or other areas of your manuscript. We will only publish funding information present in the Funding Statement section of the online submission form. Please remove any funding-related text from the manuscript.

5. In the online submission form, you indicated that your data is available only on request from a third party. Please note that your Data Availability Statement is currently missing contact details for the third party, such as an email address or a link to where data requests can be made. Please update your statement with the missing information.

6. PLOS requires an ORCID iD for the corresponding author in Editorial Manager on papers submitted after December 6th, 2016. Please ensure that you have an ORCID iD and that it is validated in Editorial Manager. To do this, go to ‘Update my Information’ (in the upper left-hand corner of the main menu), and click on the Fetch/Validate link next to the ORCID field. This will take you to the ORCID site and allow you to create a new iD or authenticate a pre-existing iD in Editorial Manager.

7. We note that you have included the phrase “data not shown” in your manuscript. Unfortunately, this does not meet our data sharing requirements. PLOS does not permit references to inaccessible data. We require that authors provide all relevant data within the paper, Supporting Information files, or in an acceptable, public repository. Please add a citation to support this phrase or upload the data that corresponds with these findings to a stable repository (such as Figshare or Dryad) and provide and URLs, DOIs, or accession numbers that may be used to access these data. Or, if the data are not a core part of the research being presented in your study, we ask that you remove the phrase that refers to these data.

8. Please include a separate caption for each figure in your manuscript.

9. Please upload a new copy of Figures 1, 2, and 3 as the detail is not clear. Please follow the link for more information:  https://journals.plos.org/plosone/s/figures

10. Please include captions for your Supporting Information files at the end of your manuscript, and update any in-text citations to match accordingly. Please see our Supporting Information guidelines for more information: http://journals.plos.org/plosone/s/supporting-information.

11. If the reviewer comments include a recommendation to cite specific previously published works, please review and evaluate these publications to determine whether they are relevant and should be cited. There is no requirement to cite these works unless the editor has indicated otherwise.

[Note: HTML markup is below. Please do not edit.]

Reviewers' comments:

Reviewer's Responses to Questions

**Comments to the Author**

1. Is the manuscript technically sound, and do the data support the conclusions?

Reviewer #1: Partly

Reviewer #2: Partly

**********

2. Has the statistical analysis been performed appropriately and rigorously?

Reviewer #1: No

Reviewer #2: No

**********

3. Have the authors made all data underlying the findings in their manuscript fully available?

The PLOS Data policy

Reviewer #1: Yes

Reviewer #2: Yes

**********

4. Is the manuscript presented in an intelligible fashion and written in standard English?

Reviewer #1: Yes

Reviewer #2: Yes

**********

Reviewer #1: Overall Assessment

This study presents a proof-of-concept application of targeted metabolomics to postmortem cardiac tissue, addressing a technically challenging and underexplored area. The proof-of-concept framing is appropriate given the sample size and exploratory nature of the analyses, and the manuscript provides a useful initial evaluation of the performance of a targeted platform in this context. The work has potential relevance for forensic research; however, further clarification and refinement of the analysis and presentation are required.

Major Comments

The manuscript appears to address two distinct objectives: (1) evaluating the technical feasibility of a targeted metabolomics platform in postmortem cardiac tissue, and (2) assessing biological differences between IHD, T2D, and control groups. At present, the balance between these aims is unclear, resulting in a fragmented narrative. The authors should clarify the primary focus of the study and align the narrative and interpretation accordingly, as the current presentation risks overextending biological interpretation from what is inherently a methodological proof-of-concept study.

The implementation of distance-based classification methods (maximum, centroid, Mahalanobis) alongside PLS-DA is unclear. It is not evident whether these approaches were applied within the PLS-DA latent space or used as independent classifiers. In addition, no model validation metrics (e.g., Q², permutation testing, or cross-validation strategy) are reported. This raises concerns regarding potential overfitting and the robustness of the reported classification performance.

The reporting of FDR-adjusted p-values is unclear. The statement that all metabolites have an identical adjusted p-value (p = 0.628) appears implausible, as Benjamini–Hochberg correction typically produces a distribution of adjusted p-values. The authors should clarify how FDR correction was implemented and how these values are being reported.

The PERMANOVA results indicate no significant differences in overall metabolite composition between groups (R² = 0.051, p = 0.429), which is not consistent with the apparent separation suggested by the PLS-DA analysis. This discrepancy should be addressed. Furthermore, it is not stated whether homogeneity of dispersion was assessed, which is a key assumption underlying PERMANOVA.

Table 3 presents metabolites selected on the basis of unadjusted p-values (< 0.05), despite the application of FDR correction indicating that none remain statistically significant. Presenting these features without adjusted p-values risks overstating their relevance. Adjusted p-values should be included alongside raw p-values for transparency of reporting.

The PLS-DA visualisation uses convex hulls to outline group boundaries; however, these are sensitive to extreme observations. The broad hulls observed—particularly for the IHD and T2D groups—suggest that the apparent separation may be influenced by a small number of outliers rather than consistent clustering. This raises concerns about overinterpretation, and the influence of outliers on the model should be assessed or discussed appropriately.

Although the PMI range (3–8 days) is described as relatively narrow, metabolite levels are known to change over this interval in humans. In the context of a feasibility study, PMI should be considered not only as a confounder but also as a variable of interest. Assessing how metabolite detection or quantification rates vary with PMI would strengthen the study and provide insight into the robustness of the assay under postmortem conditions.

Minor Comments

The proof-of-concept nature of the study is stated in the main text but would benefit from being more clearly indicated in the title and/or abstract to appropriately frame the scope and expectations of the work.

There is an inconsistency in the definition of ischemic heart disease (IHD) between the Methods and Results. In the Methods, IHD is defined as ≥75% coronary artery stenosis, whereas in the Results, most IHD cases are described as having ≥50% stenosis. This discrepancy should be clarified and corrected.

In the Discussion, the authors state that the study groups were broadly matched; however, no statistical comparisons of baseline characteristics are presented to support this claim. Appropriate between-group comparisons should be included, or the statement should be revised.

Figure 2 caption appears to retain “tracked changes” from multiple authors, please finalise this caption accordingly.

The corresponding author does not match between the manuscript submission system and the written manuscript version, please clarify accordingly.

Reviewer #2: The manuscript addresses a relevant and timely topic, namely the application of a standardized targeted metabolomics platform to postmortem human cardiac tissue. This represents a meaningful methodological effort, and the authors should be commended for their transparency, particularly in clearly reporting that no metabolites remained significant after correction for multiple testing. However, in its current form, the manuscript presents a mismatch between its stated objectives and what the data are able to support, and therefore requires substantial revision before it can be considered for publication.

A) The manuscript requires a clear conceptual repositioning. At present, it simultaneously presents itself as a feasibility/methodological study and as a biological comparison between T2D, IHD, and control groups. The data do not support the latter. The absence of FDR-significant metabolites, the non-significant PERMANOVA, and the weak multivariate separation indicate that no robust biological discrimination has been demonstrated. The authors should explicitly reframe the study as a feasibility investigation and revise the title, abstract, and conclusions accordingly. Any biological findings should be clearly downgraded to exploratory observations, and all language suggesting group discrimination should be removed or substantially toned down.

B) The impact of postmortem interval (PMI) is a central issue that is currently underdeveloped and must be addressed more rigorously. The reported PMI range (3–8 days) is biologically relevant and likely to influence metabolite stability. Moreover, the apparent imbalance between groups (with longer PMI in controls) introduces a potential systematic bias. The authors should explicitly investigate PMI as a confounding factor, for example by assessing correlations between PMI and major metabolite classes, visualizing multivariate analyses colored by PMI, or discussing the stability of results across the PMI range. This is particularly important given that existing forensic metabolomics literature has consistently identified PMI as a dominant driver of metabolomic variability, often exceeding disease-related effects and requiring explicit standardization strategies.

C) The heterogeneity of the study groups significantly limits interpretability and should be explicitly acknowledged and incorporated into the discussion. The T2D group includes individuals with heterogeneous causes of death (including traumatic and ischemic conditions), while the IHD group includes both acute and chronic cases. This creates overlapping biological categories and likely explains the substantial overlap observed in PCA and other analyses. The authors should revise the interpretation to reflect that group comparisons are intrinsically limited by this design and should be considered hypothesis-generating only.

D) The choice of analytical platform and its adaptation to postmortem tissue requires a more in-depth and central discussion. The use of a metabolomics kit validated for biofluids introduces well-known issues when applied to solid postmortem tissues, including matrix effects, extraction variability, and dynamic range mismatch. The observed limitations (e.g., saturation of certain metabolite classes and low detectability of others) should be treated as core findings of the feasibility analysis rather than secondary remarks. In this context, it would be useful for the authors to frame their observations within the broader literature, where the choice of biological matrix and its postmortem stability has been shown to critically influence metabolomic outputs and their forensic interpretability.

E) The statistical analysis, while based on appropriate methods, is not sufficiently rigorous in its interpretation. In particular, the use of PLS-DA in a small sample size/high-dimensional setting carries a substantial risk of overfitting. The authors should explicitly acknowledge this limitation and avoid any implication of discriminatory performance. If retained, PLS-DA results should be clearly presented as exploratory, and their limitations should be emphasized. Additionally, the interpretation of metabolites showing nominal trends but no significance after FDR correction should be explicitly framed as non-conclusive.

F) The handling of values below the limit of detection should be more critically discussed. The use of imputation strategies such as LOD/2 is common, but in this dataset—where entire metabolite classes are affected—it may influence downstream statistical analyses. The authors should discuss the potential impact of this choice and its implications for the robustness of their findings.

G) The Discussion requires restructuring to better align with the data. The conclusions should focus on what has been convincingly demonstrated, namely the partial analytical feasibility of the approach and the class-dependent performance of the platform. Unsupported biological interpretations should be removed or clearly separated from the main conclusions.

With substantial revision along these lines, the manuscript could make a useful methodological contribution to the field of postmortem metabolomics. In its current form, however, the interpretation exceeds the strength of the data and requires significant adjustment.

**********

what does this mean?). If published, this will include your full peer review and any attached files.

If you choose “no”, your identity will remain anonymous but your review may still be made public.

**Do you want your identity to be public for this peer review?**  For information about this choice, including consent withdrawal, please see our Privacy Policy

Reviewer #1: No

Reviewer #2: No

**********

[NOTE: If reviewer comments were submitted as an attachment file, they will be attached to this email and accessible via the submission site. Please log into your account, locate the manuscript record, and check for the action link "View Attachments". If this link does not appear, there are no attachment files.]

To ensure your figures meet our technical requirements, please review our figure guidelines: https://journals.plos.org/plosone/s/figures

You may also use PLOS’s free figure tool, NAAS, to help you prepare publication quality figures: https://journals.plos.org/plosone/s/figures#loc-tools-for-figure-preparation.

NAAS will assess whether your figures meet our technical requirements by comparing each figure against our figure specifications.

# Author comment: Author response to Decision Letter 1

**DOI:** 10.1371/journal.pone.0356269.r002

2 Jun 2026

Response to Reviewers and Editor

Manuscript ID: PONE-D-26-02704

Title: Targeted metabolomics of postmortem human cardiac tissue using the Biocrates MxP® Quant 500 kit

Dear Editor and Reviewers,

We would like to thank the editor and reviewers for their thorough and constructive evaluation of our manuscript. We have carefully revised the manuscript in response to all comments, and we believe that these revisions have strengthened the manuscript. In particular, we have clarified the proof-of-concept nature of the study, strengthened the statistical reporting, and expanded the analysis of postmortem interval (PMI).

For an overview of each comment and its changes, please see below. The the changed lines we refer to can be found in the tracked version.

Reviewer #1

Major Comment 1: Study aims and narrative clarity

Comment: The manuscript appears to address two distinct objectives: (1) evaluating the technical feasibility of a targeted metabolomics platform in postmortem cardiac tissue, and (2) assessing biological differences between IHD, T2D, and control groups. At present, the balance between these aims is unclear, resulting in a fragmented narrative. The authors should clarify the primary focus of the study and align the narrative and interpretation accordingly, as the current presentation risks overextending biological interpretation from what is inherently a methodological proof-of-concept study.

Response:

We agree with the reviewer that the original manuscript did not clearly distinguish between the primary methodological aim and the exploratory biological comparisons. We have therefore revised the manuscript to clearly position the study as a proof-of-concept feasibility investigation of the Biocrates MxP® Quant 500 kit applied to postmortem human cardiac tissue.

Biological comparisons between the study groups are now explicitly framed as exploratory and hypothesis-generating. We have toned down or removed language suggesting clear group discrimination of the metabolites and clarified that observed group differences were nominal findings based on unadjusted p-values and did not remain statistically significant after FDR correction.

These revisions have been made throughout the manuscript, including the Abstract (lines 21–51), Introduction (lines 120–128), Results (lines 313–375), Discussion (lines 530–590), and Conclusions (lines 611–626).

Major Comment 2

Comment: The implementation of distance-based classification methods (maximum, centroid, Mahalanobis) alongside PLS-DA is unclear. It is not evident whether these approaches were applied within the PLS-DA latent space or used as independent classifiers. In addition, no model validation metrics (e.g., Q², permutation testing, or cross-validation strategy) are reported. This raises concerns regarding potential overfitting and the robustness of the reported classification performance.

Response:

Reviewer #1 and #2 both raised concerns regarding the use of the PLS-DA and how it was presented in the original manuscript, and we agree with these concerns. Based on the reviewers’ input we decided to remove the PLS-DA altogether from the manuscript. The PLS-DA did not provide main findings for the study, and the analysis is not well suited for this type of data.

This includes removal of the description of PLS-DA from the Statistical analysis section of the Materials and Methods (lines 242–245), removal of the PLS-DA results ( lines 329–351), and removal of the interpretation from the Discussion (lines 506–512).

Eliminating it reduces the risk of overextending biological interpretation of the results – thus further addressing other comments from reviewer 1 (Major comment #1) and reviewer 2 (Comment A).

Major Comment 3

Comment: The reporting of FDR-adjusted p-values is unclear. The statement that all metabolites have an identical adjusted p-value (p = 0.628) appears implausible, as Benjamini–Hochberg correction typically produces a distribution of adjusted p-values. The authors should clarify how FDR correction was implemented and how these values are being reported.

Response:

We have clarified the implementation of the Benjamini–Hochberg false discovery rate (FDR) correction in the Method section and revised the Result section to improve clarity. FDR correction was applied across all retained metabolites tested in the Kruskal–Wallis analysis, treating them as a single set of simultaneous statistical tests. We now report FDR-adjusted p-values alongside raw p-values in Table 3 and explicitly state that no metabolites remained significant after correction. The wording has been revised to avoid implying that all metabolites had an identical adjusted p-value.

These changes have been made in the Methods section (lines 222-224), Results section (lines 355-360), and Table 3. Further all p-values that were reported as being statistically significant, have now been changed to being reported as nominally significant.

Major Comment 4

Comment: The PERMANOVA results indicate no significant differences in overall metabolite composition between groups (R² = 0.051, p = 0.429), which is not consistent with the apparent separation suggested by the PLS-DA analysis. This discrepancy should be addressed. Furthermore, it is not stated whether homogeneity of dispersion was assessed, which is a key assumption underlying PERMANOVA.

Response:

We acknowledge this discrepancy and appreciate the reviewer’s concern. We have addressed this by explicitly reporting the non-significant PERMANOVA results and by adding a test for homogeneity of multivariate dispersion (PERMDISP). As the PLS-DA analysis has now been removed from the revised manuscript, the previous discrepancy between PERMANOVA and PLS-DA no longer applies. We also believe that adding PERMDISP provides additional interpretational value to the dataset. The Results section now clarifies that the observed variation primarily reflects within-group heterogeneity rather than clear group separation.

These revisions have been incorporated into the Methods section (lines 229-231), Results section (lines 370-373), as well as the lines related to PLS-DA, as described in the response to Major Comment 2.

Major Comment 5

Comment: Table 3 presents metabolites selected on the basis of unadjusted p-values (< 0.05), despite the application of FDR correction indicating that none remain statistically significant. Presenting these features without adjusted p-values risks overstating their relevance. Adjusted p-values should be included alongside raw p-values for transparency of reporting.

Response:

As described in the response to Major Comment 3, we have revised Table 3 to include both unadjusted and FDR-adjusted p-values. We have also revised the Results section to explicitly describe these findings as nominal (unadjusted) and exploratory, as no metabolites remained statistically significant after FDR correction.

These revisions are included in the Results section (lines 384 for Table 3).

Major Comment 6

Comment: The PLS-DA visualization uses convex hulls to outline group boundaries; however, these are sensitive to extreme observations. The broad hulls observed—particularly for the IHD and T2D groups—suggest that the apparent separation may be influenced by a small number of outliers rather than consistent clustering. This raises concerns about overinterpretation, and the influence of outliers on the model should be assessed or discussed appropriately.

Response:

We agree with the reviewer’s concern. The PLS-DA analysis has been removed from the revised manuscript. This removes the risk of overinterpreting apparent group boundaries driven by outliers

These changes are similar to those described in response to Major Comment 2.

Major Comment 7

Comment: Although the PMI range (3–8 days) is described as relatively narrow, metabolite levels are known to change over this interval in humans. In the context of a feasibility study, PMI should be considered not only as a confounder but also as a variable of interest. Assessing how metabolite detection or quantification rates vary with PMI would strengthen the study and provide insight into the robustness of the assay under postmortem conditions.

Response:

We thank the reviewer for this important comment and agree that PMI should be considered not only as a potential confounder but also as a variable of interest in a feasibility study. We have therefore expanded the analysis of PMI as follows:

• Performed Spearman correlation analyses between PMI and metabolite class scores based on z-score normalized concentrations.

• Added PCA plots colored by PMI, now included as Fig S1

• Summarized the results in the main text and S2 Table

These analyses showed that PMI contributes to variability in certain metabolite classes. However, the observed associations were generally weak to moderate and did not remain statistically significant after FDR correction. PCA likewise did not reveal clear clustering by PMI. We have also clarified that PMI is an uncertain proxy for postmortem degradation, as degradation is influenced by factors such as temperature, humidity, storage conditions, and environmental exposure. Therefore, visible putrefaction was used as an additional pragmatic exclusion criterion to limit bias from advanced postmortem degradation.

Taken together, these revisions indicate that, within the studied PMI range, the PMI was not a dominant factor influencing the overall metabolite profile.

These revisions have made in the Introduction (lines 84–90), Methods section (lines 155–156, 159-166 and 232–236), Results section (lines 306–311, 318-322), Discussion (lines 490-498).

Minor Comment 1

Comment: The proof-of-concept nature of the study is stated in the main text but would benefit from being more clearly indicated in the title and/or abstract to appropriately frame the scope and expectations of the work.

Response: We have clarified the proof-of-concept nature of the study in the Abstract, including the first sentence describing the study aim. We believe the title already reflects the methodological focus of the study, and therefore we did not deem a title change necessary.

This revision has been made in the Abstract (lines 22–24).

Minor Comment 2

Comment: There is an inconsistency in the definition of ischemic heart disease (IHD) between the Methods and Results. In the Methods, IHD is defined as ≥75% coronary artery stenosis, whereas in the Results, most IHD cases are described as having ≥50% stenosis. This discrepancy should be clarified and corrected.

Response: We have clarified this discrepancy by revising Table 1 and expanding the reporting of coronary artery stenosis. The other coronary arteries have now been added to Table 1, and stenosis degree has been stratified as mild, moderate, moderately severe, and severe. We also added the group comparison tests to the Methods section and updated the Results text accordingly.

These revisions have been made in the Methods section (lines 174–177 and 219–221), Results section (lines 257–275), and Table 1.

Minor Comment 3

Comment: In the Discussion, the authors state that the study groups were broadly matched; however, no statistical comparisons of baseline characteristics are presented to support this claim. Appropriate between-group comparisons should be included, or the statement should be revised.

Response: We thank the reviewer for this comment. We have revised the manuscript to address this point by including statistical comparisons of baseline characteristics between groups, which are now presented in Table 1 with corresponding p‑values calculated using Kruskal–Walli’s test. Based on these analyses, the groups were comparable with respect to age, BMI, PMI, heart weight, and left ventricular wall thickness, while expected differences were observed for coronary artery stenosis. The text has been updated accordingly to ensure that statements regarding group comparability are supported by the reported statistical analyses.

Minor Comment 4

Comment: Figure 2 caption appears to retain “tracked changes” from multiple authors, please finalise this caption accordingly.

Response: We thank the reviewer for noticing this. The Fig 2 caption has now been finalized, and all figure legends have been reviewed and inserted into the manuscript.

Minor Comment 5:

Comment: The corresponding author does not match between the manuscript submission system and the written manuscript version, please clarify accordingly.

Response: This has now been corrected so that the corresponding author information is consistent between the manuscript and the submission system. We thank the reviewer for pointing this out.

Reviewer #2

Comment A)

The manuscript requires a clear conceptual repositioning. At present, it simultaneously presents itself as a feasibility/methodological study and as a biological comparison between T2D, IHD, and control groups. The data do not support the latter. The absence of FDR-significant metabolites, the non-significant PERMANOVA, and the weak multivariate separation indicate that no robust biological discrimination has been demonstrated. The authors should explicitly reframe the study as a feasibility investigation and revise the title, abstract, and conclusions accordingly. Any biological findings should be clearly downgraded to exploratory observations, and all language suggesting group discrimination should be removed or substantially toned down.

Response: We agree fully with the reviewer’s assessment and have addressed this concern in parallel with Reviewer #1, Major Comment 1, which raised a closely related issue regarding the clarity of the study’s aims and narrative focus. The manuscript has now been comprehensively reframed as a feasibility‑focused, methodological proof‑of‑concept study.

Accordingly, the Abstract, Results, Discussion, and Conclusions have all been revised to clearly reflect this position. Biological comparisons between T2D, IHD, and control groups are now explicitly described as exploratory, and all language suggesting robust group discrimination has been removed or substantially toned down. We also explicitly state that no metabolites remained significant after FDR correction and that PERMANOVA did not show significant group differences. We consider the absence of clear group discrimination to be an important feasibility-related finding, as it highlights the limitations of exploratory biological interpretation in this sample set.

These revisions have been made in the Abstract (lines 21-26, 45-49), Results (lines 319-322, 356-365, 366-373), Discussion (lines 504-538, with removal of lines 539-564), and Conclusions (lines 588–600), as also described in the responses to Reviewer #1, Major Comments 1–5.

Comment B) The impact of postmortem interval (PMI) is a central issue that is currently underdeveloped and must be addressed more rigorously. The reported PMI range (3–8 days) is biologically relevant and likely to influence metabolite stability. Moreover, the apparent imbalance between groups (with longer PMI in controls) introduces a potential systematic bias. The authors should explicitly investigate PMI as a confounding factor, for example by assessing correlations between PMI and major metabolite classes, visualizing multivariate analyses colored by PMI, or discussing the stability of results across the PMI range. This is particularly important given that existing forensic metabolomics literature has consistently identified PMI as a dominant driver of metabolomic variability, often exceeding disease-related effects and requiring explicit standardization strategies.

Response: This was concern was also addressed by reviewer #1 and we agree that it requires more attention. We now made revisions to explicitly evaluate the PMI by performing Spearman correlation analyses between PMI and metabolite class scores based on z‑score–normalized concentrations, and by adding PCA visualizations colored by PMI to assess potential PMI‑related structure in the data. The statistical approach is described in the Methods section, and PMI‑related results are reported in the main text and in S2 Table.

After re‑examining the dataset, the reported PMI range was corrected from 3–8 days to 3–9 days, and all PMI‑related descriptions have been updated ac

Attachment Submitted filename: Response to editor and reviewer.18.5.26.docx

# Peer review: Decision Letter 1

**DOI:** 10.1371/journal.pone.0356269.r003

**Authors:** Nguyen Phuoc Long

30 Jun 2026

Dear Dr. Mardal,

Thank you for submitting your manuscript to PLOS ONE. After careful consideration, we feel that it has merit but does not fully meet PLOS ONE’s publication criteria as it currently stands. Therefore, we invite you to submit a revised version of the manuscript that addresses the points raised during the review process.

Please submit your revised manuscript by Aug 14 2026 11:59PM. If you will need more time than this to complete your revisions, please reply to this message or contact the journal office at plosone@plos.org. When you're ready to submit your revision, log on to https://www.editorialmanager.com/pone/ and select the 'Submissions Needing Revision' folder to locate your manuscript file.

- A letter that responds to each point raised by the academic editor and reviewer(s). You should upload this letter as a separate file labeled 'Response to Reviewers'.
- A marked-up copy of your manuscript that highlights changes made to the original version. You should upload this as a separate file labeled 'Revised Manuscript with Track Changes'.
- An unmarked version of your revised paper without tracked changes. You should upload this as a separate file labeled 'Manuscript'.

If you would like to make changes to your financial disclosure, please include your updated statement in your cover letter. Guidelines for resubmitting your figure files are available below the reviewer comments at the end of this letter.

If applicable, we recommend that you deposit your laboratory protocols in protocols.io to enhance the reproducibility of your results. Protocols.io assigns your protocol its own identifier (DOI) so that it can be cited independently in the future. For instructions see: https://journals.plos.org/plosone/s/submission-guidelines#loc-laboratory-protocols. Additionally, PLOS ONE offers an option for publishing peer-reviewed Lab Protocol articles, which describe protocols hosted on protocols.io. Read more information on sharing protocols at https://plos.org/protocols?utm_medium=editorial-email&utm_source=authorletters&utm_campaign=protocols.

As the corresponding author, your ORCID iD is verified in the submission system and will appear in the published article. PLOS supports the use of ORCID, and we encourage all coauthors to register for an ORCID iD and use it as well. Please encourage your coauthors to verify their ORCID iD within the submission system before final acceptance, as unverified ORCID iDs will not appear in the published article. *Only*  the individual author can complete the verification step; PLOS staff *cannot*  verify ORCID iDs on behalf of authors.

We look forward to receiving your revised manuscript.

Kind regards,

Nguyen Phuoc Long, M.D., Ph.D.

Academic Editor

PLOS One

Journal Requirements:

If the reviewer comments include a recommendation to cite specific previously published works, please review and evaluate these publications to determine whether they are relevant and should be cited. There is no requirement to cite these works unless the editor has indicated otherwise.

Please review your reference list to ensure that it is complete and correct. If you have cited papers that have been retracted, please include the rationale for doing so in the manuscript text, or remove these references and replace them with relevant current references. Any changes to the reference list should be mentioned in the rebuttal letter that accompanies your revised manuscript. If you need to cite a retracted article, indicate the article’s retracted status in the References list and also include a citation and full reference for the retraction notice.

**Additional Editor Comments:**

Dear Authors,

Following a further evaluation of your manuscript by new reviewers, we have determined that additional revisions are necessary before it can meet the standards required for publication. Please see the detailed comments below to guide your revisions.

[Note: HTML markup is below. Please do not edit.]

Reviewers' comments:

Reviewer's Responses to Questions

**Comments to the Author**

Reviewer #1: (No Response)

Reviewer #2: All comments have been addressed

Reviewer #3: (No Response)

Reviewer #4: All comments have been addressed

**********

2. Is the manuscript technically sound, and do the data support the conclusions?

Reviewer #1: Yes

Reviewer #2: Yes

Reviewer #3: Partly

Reviewer #4: Yes

**********

3. Has the statistical analysis been performed appropriately and rigorously?

Reviewer #1: Yes

Reviewer #2: Yes

Reviewer #3: Yes

Reviewer #4: Yes

**********

4. Have the authors made all data underlying the findings in their manuscript fully available?

The PLOS Data policy

Reviewer #1: Yes

Reviewer #2: Yes

Reviewer #3: No

Reviewer #4: Yes

**********

5. Is the manuscript presented in an intelligible fashion and written in standard English?

Reviewer #1: Yes

Reviewer #2: (No Response)

Reviewer #3: Yes

Reviewer #4: Yes

**********

Reviewer #1: The authors have addressed my previous major concerns satisfactorily. The manuscript is substantially improved, and the conclusions are now appropriately aligned with the data.

I have only one minor suggestion remaining, that would help the narrative flow: The Introduction could be restructured to better reflect the methodological focus by introducing the feasibility rationale first, with the biological background second serving as motivation rather than the primary narrative.

Reviewer #2: Thank you for your careful and constructive revision. The manuscript has improved substantially and addresses the major concerns raised during the previous review rounds. In particular, the study has been appropriately reframed as a proof-of-concept feasibility investigation, the statistical interpretation has been strengthened, and the conclusions are now more closely aligned with the data presented. I have no further substantive comments and consider the manuscript suitable for publication.

Reviewer #3: (No Response)

Reviewer #4: The authors responded satisfactory to all issues raised. No further experiments or evaluations are required to support message of the manuscript.

**********

what does this mean?). If published, this will include your full peer review and any attached files.

If you choose “no”, your identity will remain anonymous but your review may still be made public.

**Do you want your identity to be public for this peer review?**  For information about this choice, including consent withdrawal, please see our Privacy Policy

Reviewer #1: No

Reviewer #2: **Yes:** Matteo Nioi

Reviewer #3: No

Reviewer #4: **Yes:** Jerzy Adamski

**********

[NOTE: If reviewer comments were submitted as an attachment file, they will be attached to this email and accessible via the submission site. Please log into your account, locate the manuscript record, and check for the action link "View Attachments". If this link does not appear, there are no attachment files.]

To ensure your figures meet our technical requirements, please review our figure guidelines: https://journals.plos.org/plosone/s/figures

You may also use PLOS’s free figure tool, NAAS, to help you prepare publication quality figures: https://journals.plos.org/plosone/s/figures#loc-tools-for-figure-preparation.

NAAS will assess whether your figures meet our technical requirements by comparing each figure against our figure specifications.

Attachment Submitted filename: PONE-D-26-02704R1-Comments.docx

# Author comment: Author response to Decision Letter 2

**DOI:** 10.1371/journal.pone.0356269.r004

29 Jul 2026

We paste the respond to reviewers here as requested, but it is easier for you to read in the attached word document.

Response to reviewers round 2

We thank the Editor and the Reviewers for their careful evaluation of our manuscript and for their constructive comments and suggestions. We have revised the manuscript accordingly and provide a detailed, point-by-point response below. Quotes in the rebuttal from modified text are highlighted in orange. Unmodified parts of the quotes are black.

Reviewer #1

The authors have addressed my previous major concerns satisfactorily. The manuscript is substantially improved, and the conclusions are now appropriately aligned with the data.

I have only one minor suggestion remaining, that would help the narrative flow: The Introduction could be restructured to better reflect the methodological focus by introducing the feasibility rationale first, with the biological background second serving as motivation rather than the primary narrative.

Authors Response: We thank the reviewer for the acknowledgement of our correction. We restructured the introduction (lines: 54-115 - not quoted here due to length) with the feasibility rationale first, which better aligns with the narrative flow of the manuscript.

Reviewer #2

Thank you for your careful and constructive revision. The manuscript has improved substantially and addresses the major concerns raised during the previous review rounds. In particular, the study has been appropriately reframed as a proof-of-concept feasibility investigation, the statistical interpretation has been strengthened, and the conclusions are now more closely aligned with the data presented. I have no further substantive comments and consider the manuscript suitable for publication.

Reviewer #3

(No Response)

Reviewer #4

The authors responded satisfactory to all issues raised. No further experiments or evaluations are required to support message of the manuscript.

Reviewer #5 (new)

This manuscript presents a proof-of-concept study evaluating the feasibility of applying the Biocrates Life Sciences MxP® Quant 500 kit to postmortem human cardiac tissue obtained during forensic autopsies. The authors aim to determine whether this standardized targeted metabolomics platform, originally developed for biofluids, can be extended to solid tissue analysis and whether it may support forensic investigations through comparison of metabolomic profiles across different groups of sudden cardiac death

While the topic is relevant and of potential interest to forensic metabolomics, the current manuscript has important methodological limitations that prevent a full assessment of the validity of the conclusions. The following major issues should be addressed:

1. The manuscript aims to evaluate the feasibility and analytical performance of the MxP® Quant 500 kit in postmortem cardiac tissue. However, key analytical validation parameters (e.g., precision, recovery, matrix effects, limits of detection, and QC performance) are not reported, and no clear optimization of tissue-specific extraction or processing is described. In the absence of these data, the analytical suitability of the platform for postmortem cardiac tissue cannot be fully assessed. Consequently, the absence of statistically significant metabolite differences between cause-of-death groups cannot be confidently interpreted as reflecting biological variation alone.

Authors Response: We thank the reviewer for this suggestion. To strengthening the analytical validation section, we have added a new Supplementary Information table (SI1 Table - shifting the number of the other SI Tables) reporting key analytical performance metrics for all measured metabolites, including many of the variables requested: limits of detection, lower and upper limits of quantitation, QC performance at each concentration level, accuracy assessment, together with precision. These are indeed very important metrics to address analytical suitability, and we should have added it to begin with.

The methodological detail regarding the tissue preparation was not sufficiently clear. We described the sample extraction method in more detail and clarified, with references, why this was selected. The requested optimization of tissue-specific extraction or processing has already, in large, been performed by the referenced paper by Andresen et al. in other postmortem matrices. In the updated method section, we specified what optimization parameters were already covered for postmortem matrices by this paper. An important source of information missing from this Andresen paper is median measured values, and analytical performance - we have this in our manuscript in SI1 and SI2 Tables.

Modifications: “The samples were placed in cryotubes and stored at −70°C immediately after the autopsy and later thawed and homogenized at a 1:3 ratio with isopropanol. This single extraction with 100% isopropanol was selected based on the procedure reported by Andresen et al. [10] who evaluated different extraction media and tissue-to-solvent ratios for human tissue analyzed with the same kit. The tissue was homogenized using a gentleMACS Octo Dissociator (Miltenyi Biotec, Bergisch Gladbach, Germany) with a 50–second program applying up to 4000 rpm. The instrumentation is routinely used for postmortem muscle and brain tissue homogenization prior to quantitative analysis in forensic case work with the same tissue-to-solvent ratio, although different extraction media optimized for a different group of analytes [10,34,35]. Approximately 500 mg of tissue was processed per case, and homogenate supernatants were stored at −70°C until further analysis.” (lines: 163-170)

2. It would be helpful to clarify the rationale for selecting a biofluid-optimized kit for the analysis of solid tissue samples. The manuscript could be strengthened by including a more detailed description of the tissue-specific sample preparation and extraction procedures, including the homogenization method, extraction workflow, and normalization strategy, to support reproducibility of the analytical approach. In addition, reporting missing data rates and the number of metabolites excluded due to QC criteria would further improve the evaluation of platform performance in this matrix. If no specific optimization was performed, this should be clearly stated, along with a brief discussion of the potential implications for data interpretation.

Authors Response: We thank the reviewer for this valuable suggestion that builds on some of the same points as mentioned in issue #1. The new information we provided in SI1 Table complements the methodological description and provides readers with additional insight into assay performance across the measured metabolite panel. We further included a summary for analytical performance of the metabolites in the results section. There is no commercially available metabolomics kit, to our knowledge, for cardiac tissue, but (as mentioned in the introduction, lines 83-85) this kit has already been used for several alternative matrices. The methodological detail regarding the tissue preparation was not sufficiently clear. We described the sample extraction method in more detail and clarified, with references, why this was selected (se modification under issue #1).

Modifications: We here show a snip of SI1 Table to display the variables presented. The new table was referenced at lines 257, 307, 320, and 377. In addition, we added asterisk to Table 3 for metabolites that had at least one QC failed on either coefficient of variation or accuracy.

(lines 305-310): “If we look at the analytical performance of the metabolites with unadjusted p < 0.05 in the Kruskal-Wallis test (Table 3), the triglycerides and PC O-30:1 had at least one failed QC (S1 Table), either for the coefficient of variation or accuracy, measured at three levels with at least three QCs at each level. At least one failed QC for one parameter is indicated with an asterisk in Table 3. The remaining ten metabolites passed all set parameters, with the medium-level QC coefficient of variation below 12% (N = 4).”

3. It may be worth clarifying whether signal saturation was observed for any compounds, and if so, whether this influenced considerations regarding optimization for cardiac tissue using this kit. Although the study employs a broad targeted metabolomics/lipidomics platform, it should be noted that key mitochondrial phospholipids such as cardiolipins, which are highly abundant and functionally important in cardiac tissue, are not covered by the MxP® Quant 500 kit. This limitation should be acknowledged when interpreting the biological scope of the lipidomic findings, particularly in relation to mitochondrial function and energy metabolism.

Authors Response: To address concerns related to the analytical measurement range, we have included a Supplementary Information SI1 Table reporting the lower and upper limits of quantitation for all measured metabolites, allowing readers to assess whether measurements approached the validated analytical range. This additional information provides greater transparency regarding the quantitative performance of the assay across the metabolite panel. SI2 Table already has some of the information requested: Here the proportion of values <LOD, <LLOQ, and >ULOQ are presented for the metabolites we measure in our postmortem cardiac tissue. This is particularly pronounced for fatty acids, since these are measured with FIA- and not LC-MS/MS - this additional point was added.

Modifications: In the discussion section added a note on cardiolipins (lines 396-398): “A notable group of metabolites not covered by this kit is cardiolipins (important for cardiac mitochondrial function) whose altered metabolism has been associated with cardiac and metabolic disorders [41].” Where 41 is “Bautista JS, Falabella M, Flannery PJ, Hanna MG, Heales SJR, Pope SAS, et al. Advances in methods to analyse cardiolipin and their clinical applications. TrAC - Trends in Analytical Chemistry. Elsevier B.V.; 2022. doi:10.1016/j.trac.2022.116808”

Lines 375-379: “Several classes, such as acylcarnitines and biogenic amines, showed a high proportion of values below the LOD, suggesting that these analytes may have required a greater sample volume to increase the analyte concentration. Conversely, amino acids and fatty acids displayed saturation effects, with > 30% of values exceeding the ULOQ in some cases, indicating that these abundant analytes may benefit from sample dilution. Most fatty acids in this kit are analyzed by FIA-MS/MS, a platform with different analytical characteristics than LC-MS/MS, including dynamic range. This is reflected in a higher number of high- and low-level QCs with CV error in this group (S1 Table); if this group of analytes is of particular interest, a lipidomics method or, alternatively, the MxP Quant 500 XL kit would be a better choice.”

4. Given that the study primarily aims to assess the feasibility and analytical performance of the kit, the interpretation of cohort-based comparisons would benefit from prior or concurrent analytical validation and optimization of the platform in postmortem cardiac tissue. In addition, comparison with an appropriate biofluid control, analyzed using the same platform, could provide useful context regarding how the kit performs in its original application relative to cardiac tissue. Furthermore, a summary table comparing metabolite coverage in this study with that reported in previous studies, if available in the literature, would help contextualize the applicability and analytical scope of the current approach.

Authors Response: The metabolomics kit used came with several biofluid control samples (as presented in SI). Unfortunately, NIST does not produce SRM for metabolites in postmortem cardiac tissue. We used extraction-solvent-matched blank samples, and quality control samples at three concentration levels for each metabolite were analyzed. Additionally, some metabolites were quantified with seven-point calibration curves. These were included in our analytical run. With the additional information we provided in SI1 Table, it is now possible to see analytical performance for all metabolites.

We evaluated the suggestion on the additional table summarizing number of metabolites analyzed in other studies. There are excellent hypothesis-driven studies that only quantify a handful of preselected targets, and exploratory untargeted metabolomics studies using metabolites annotated without cerebral oversight reporting metabolites in the thousands. The extensive discussion needed to contextualize this would be better suited for a review-style paper.

5. The manuscript should clearly distinguish between the analytical feasibility of the metabolomics platform in postmortem cardiac tissue and the biological or forensic interpretation of metabolite differences between cause-of-death groups. At present, these objectives appear to be partially conflated. Instead of presenting the study primarily as an analytical performance evaluation, the authors may consider reframing the scope to more clearly reflect a biologically exploratory or proof-of-concept application.

Authors Response: The manuscript has been evaluated by a total of five reviewers. To address comments from the first round of reviewers, the scope was more clearly focused on the analytical performance as a proof-of-concept study, and we toned down the biologically exploratory aspects. Reshaping this back would conflict with the approved modifications after first round of review.

Modifications: The updated introduction (in response to reviewer #1, lines 54-115) should improve the narrative flow of the manuscript.

Overall assessment

The manuscript presents an interesting attempt to extend a standardized targeted metabolomics platform to postmortem cardiac tissue. However, it currently lacks essential analytical validation and tissue-specific optimization data. Without these, the biological comparisons between cause-of-death groups cannot be reliably interpreted, and conclusions regarding forensic applicability are not sufficiently supported.

Strengthening the analytical validation section and clearly separating methodological feasibility from biological interpretation would substantially improve the scientific robustness and clarity of the manuscript.

Authors Response: We thank the reviewer for the valuable comments. We strengthened the section with analytical validation data/run performance data (notably with SI1 Table), clarified what postmortem tissue-specific optimization was performed in a referenced study, in and more clearly separated the methodological feasibility from biological interpretation in the introduction - to align with the order in the manuscript.

Attachment Submitted filename: OEP_Plos1_ResptoRev_2.docx

# Editor report: Decision Letter 2

**DOI:** 10.1371/journal.pone.0356269.r005

**Authors:** Nguyen Phuoc Long

2 Aug 2026

Targeted metabolomics of postmortem human cardiac tissue using the Biocrates MxP® Quant 500 kit

PONE-D-26-02704R2

Dear Dr. Mardal,

We’re pleased to inform you that your manuscript has been judged scientifically suitable for publication and will be formally accepted for publication once it meets all outstanding technical requirements.

Within one week, you’ll receive an e-mail detailing the required amendments. When these have been addressed, you’ll receive a formal acceptance letter and your manuscript will be scheduled for publication.

An invoice will be generated when your article is formally accepted. Please note, if your institution has a publishing partnership with PLOS and your article meets the relevant criteria, all or part of your publication costs will be covered. Please make sure your user information is up-to-date by logging into Editorial Manager at Editorial Manager® and clicking the ‘Update My Information' link at the top of the page. For questions related to billing, please contact billing support.

If your institution or institutions have a press office, please notify them about your upcoming paper to help maximize its impact. If they’ll be preparing press materials, please inform our press team as soon as possible -- no later than 48 hours after receiving the formal acceptance. Your manuscript will remain under strict press embargo until 2 pm Eastern Time on the date of publication. For more information, please contact onepress@plos.org.

Kind regards,

Nguyen Phuoc Long, M.D., Ph.D.

Academic Editor

PLOS One

Additional Editor Comments (optional):

Reviewers' comments:

# Editor report: Acceptance letter

**DOI:** 10.1371/journal.pone.0356269.r006

**Authors:** Nguyen Phuoc Long

PONE-D-26-02704R2

PLOS One

Dear Dr. Mardal,

I'm pleased to inform you that your manuscript has been deemed suitable for publication in PLOS One. Congratulations! Your manuscript is now being handed over to our production team.

At this stage, our production department will prepare your paper for publication. This includes ensuring the following:

* All references, tables, and figures are properly cited

* All relevant supporting information is included in the manuscript submission,

* There are no issues that prevent the paper from being properly typeset

You will receive further instructions from the production team, including instructions on how to review your proof when it is ready. Please keep in mind that we are working through a large volume of accepted articles, so please give us a few days to review your paper and let you know the next and final steps.

Lastly, if your institution or institutions have a press office, please let them know about your upcoming paper now to help maximize its impact. If they'll be preparing press materials, please inform our press team within the next 48 hours. Your manuscript will remain under strict press embargo until 2 pm Eastern Time on the date of publication. For more information, please contact onepress@plos.org.

You will receive an invoice from PLOS for your publication fee after your manuscript has reached the completed accept phase. If you receive an email requesting payment before acceptance or for any other service, this may be a phishing scheme. Learn how to identify phishing emails and protect your accounts at https://explore.plos.org/phishing.

If we can help with anything else, please email us at customercare@plos.org.

Thank you for submitting your work to PLOS ONE and supporting open access.

Kind regards,

PLOS ONE Editorial Office Staff

on behalf of

Dr. Nguyen Phuoc Long

Academic Editor

PLOS One
